# Supplementary material for: Inhibition of ATP synthase reverse activity restores energy homeostasis in mitochondrial pathologies
Source: EMBO J. 2023 Mar 13;42(10):e111699. doi: 10.15252/embj.2022111699 (PMC10183817; doi:10.15252/embj.2022111699)
Supplement: Supplementary file 1 — Appendix [file EMBJ-42-e111699-s003.pdf]

TABLE OF CONTENTS

Appendix Figures and Figure legends

Appendix Figure S1: Effect of Proteinase K in previously frozen samples on maximal respiration and maximal ATP hydrolytic capacity ……………………………………….....2

Appendix Figure S2: Epicatechin does not affect proton leak …..………………………..…3

Appendix Figure S3: Catechin does not inhibit CV ATP hydrolysis ………………………4

Appendix Figure S4: ATP hydrolysis inhibition by epicatechin in an oligomeric CV purified fraction from bovine mitochondria ……………………………………………………….5

Appendix Figure S5: Bioenergetics in mitochondrial deficient cells ………………………6

Appendix Figure S6: Mitochondrial content in gastrocnemius from *mdx* mice …………..7

Appendix Figures and Figure legends

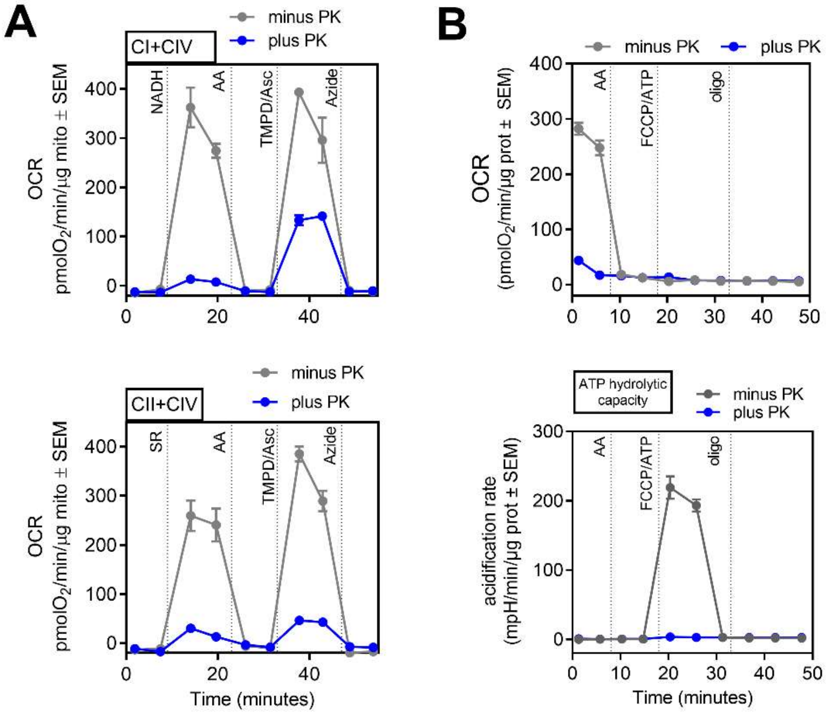

Appendix Figure S1: Effect of Proteinase K in previously frozen samples on maximal respiration and maximal ATP hydrolytic capacity

(**A**) Representative profile of RIFS respirometry in untreated or PK treated frozen mitochondria. CI and CIV (top panel) and CII and CIV (bottom panel). (**B**) Representative profiles of HyFS in untreated or PK treated frozen mitochondria. OCR (top panel) and acidification rate (bottom panel).

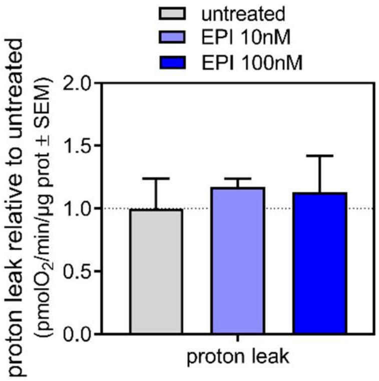

Appendix Figure S2: Epicatechin does not affect proton leak

Proton leak in isolated mitochondria from mouse heart. EPI was added in the respiration media at the indicated concentrations (n≥3). Note that EPI does not affect proton leak. For each biological replicate, technical replicates were averaged. Data represent average ± SEM.

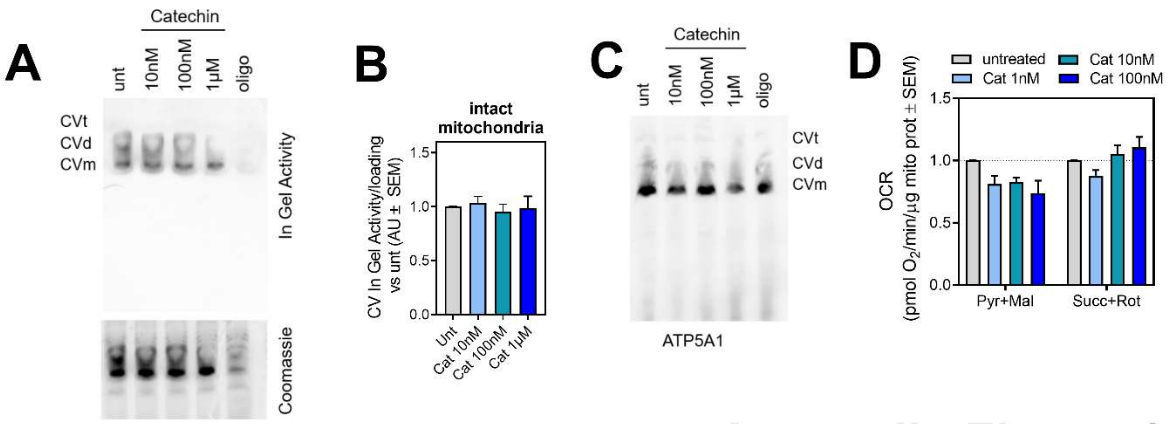

Appendix Figure S3: Catechin does not inhibit CV ATP hydrolysis.

(**A**) In gel ATP hydrolytic activity in mouse heart mitochondria incubated in the presence of the indicated concentrations of Catechin prior to freezing the mitochondrial preparation and lysing the mitochondria with digitonin. In gel activity is shown after 3hrs (top). Coomassie staining was used as loading control (bottom). Oligomycin (oligo) was used as control for ATP synthesis and hydrolysis inhibition. (**B**) Quantification of in gel ATP hydrolytic under the indicated Catechin concentrations (n=4). (**C)** Representative blot showing CV assembly by BNGE in mouse heart mitochondria incubated in the presence of the indicated concentrations of Catechin. (**D**) State 3 respiration driven by Pyr+Mal (left) and Succ+Rot (right) in isolated mitochondria from mouse heart. Catechin was added in the respiration media at the indicated concentrations (n=3). For each biological replicate, technical replicates were averaged. Data represent average ± SEM.

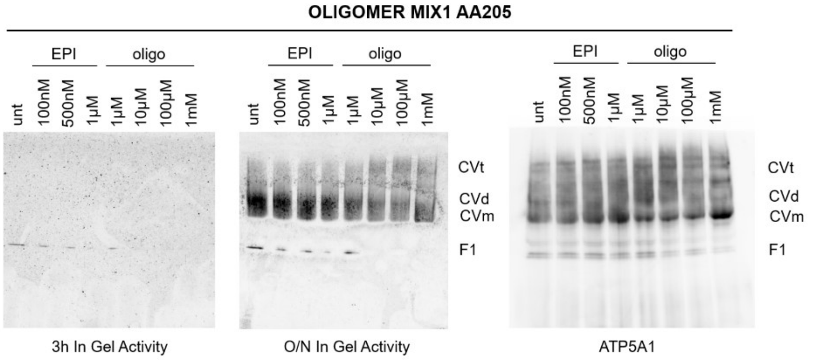

Appendix Figure S4: ATP hydrolysis inhibition by epicatechin in an oligomeric CV purified fraction from bovine mitochondria.

In gel ATP hydrolysis in oligomer mix 1 AA205 purified bovine CV preparation under the indicated EPI and oligo concentrations. In gel activity is shown after either 3hrs or O/N incubation (left) and after stopping the activity with 50% methanol (middle). Western blot for ATP5A1 was used as loading control (right).

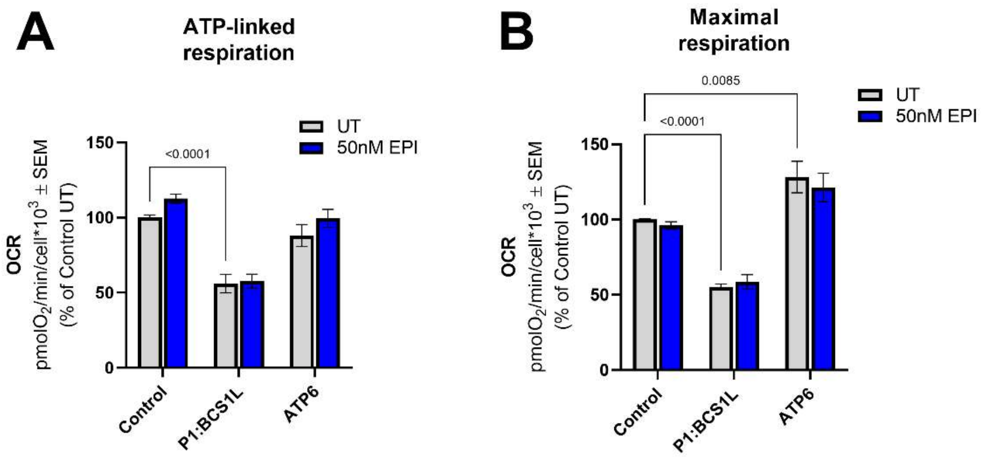

Appendix Figure S5: Bioenergetics in mitochondrial deficient cells

Bioenergetic analysis carried out in intact cells show that: **(A)** Compared to control cells, CIII-deficient fibroblasts (P1:BCS1L) have lower ATP-linked respiration, whereas CV-deficient fibroblast (ATP6) have similar levels. EPI does not influence ATP-linked respiration in any of the cell lines. **(B)** Compared to control cells, CIII-deficient cells exhibit a decrease in maximal respiration while CV-deficient cells reveal an increase in this parameter. EPI does not affect maximal respiration in any of the cell lines. Data represent average ± SEM of 8 independent biological replicates.

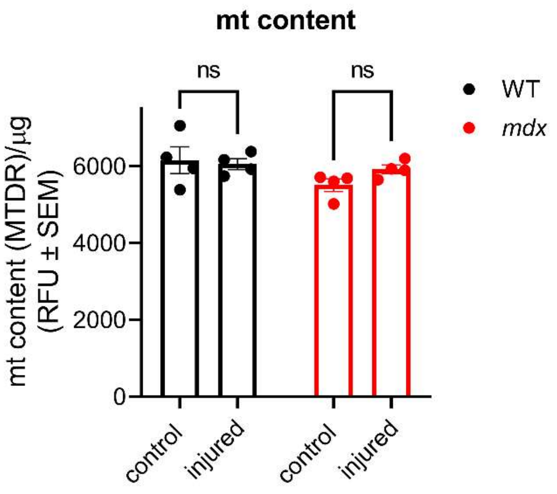

Appendix Figure S6: Mitochondrial content in gastrocnemius from *mdx* mice

Mitochondrial content is unchanged in *mdx* mice either in control or injured gastrocnemius.

Appendix S1: Expanded Experimental Information

2a. Rationale of using acidification as a read out for ATP hydrolysis measurement

Both the ATP synthase and hydrolase activities mitochondrial F_1F_O-ATP synthase can be indirectly measured in reductionist systems by monitoring pH changes in the experimental medium (Divakaruni *et al*, 2018; Sarkar & Chattopadhyay, 2017). Phosphorylation of ADP alkalinizes the experimental medium as it consumes H^+, and similarly, hydrolysis of ATP generates H^+ and acidifies the experimental medium. Specific involvement of the mitochondrial ATP synthase, distinct from other ATP hydrolyzing enzymes, is measured by acute sensitivity to low concentrations of oligomycin. The rough equation for ATP hydrolysis is given below, with the assumption that one of the two protons liberated during hydrolysis of two molecules of ATP will bind to inorganic phosphate (pKa=6.8).

$$2\ \text{Mg-ATP}^{2-} + 2\ H_2O \rightarrow 2\ \text{Mg-ADP}^{1-} + HPO_4^{2-} + H_2PO4^{1-} + H^+$$

When using the Seahorse XF Analyzer, quantitative values of ATP hydrolyzed by the ATP synthase can be obtained by calibrating the ECAR signal with known quantities of H^+ to obtain a H+ production rate (Desousa B.R. *et al*, 2022). Any reaction that results in the net change in free H^+ in the experimental medium can be detected with the pH channel of the XF Analyzer, be it the conversion of uncharged glucose to anionic lactate during glycolysis (glucose $\rightarrow$ 2 lactate^{-1} + 2H$^+$) or ATP hydrolysis in reductionist systems as detailed above (Divakaruni *et al.*, 2014). Use of the XF Analyzer to measure mitochondrial ATP hydrolysis was first conducted by McQuaker et al. (McQuaker *et al*, 2013).

2b. Studying ATP hydrolysis in previously frozen mitochondria

Frozen mitochondria (HyFS)

The term *previously frozen mitochondria* refer to mitochondria that have been frozen and thawed. In this process membrane sealing is breached to allow free diffusion. This procedure is used for respiratory analysis as we have previously shown (Acin-Perez *et al.*, 2020a). These mitochondria are completely uncoupled and do not maintain membrane potential. Previously frozen mitochondria cannot respire unless provided with direct electron donors, such as NADH or succinate to the electron transport chain complexes (Acin-Perez *et al.*, 2020a).

The analysis of ATP hydrolysis in previously frozen mitochondria is described in detail in (Fernandez-del-Rio L. *et al.*, in press)

2c. Studying ATP hydrolysis in intact mitochondria

Intact mitochondria are mitochondria isolated from cells or tissue, where the membrane integrity it preserved well enough to support respiration. Unless subjected to specific treatments, intact mitochondria pertain to mitochondria that are coupled, in which oxygen consumption is coupled to ATP synthesis. The term "coupled mitochondria" also means that the level of proton leak is maintained at physiological range for these cells or tissue.

The analysis of CV ATP hydrolysis in intact mitochondria presented additional challenges:

A. How do we assure we are looking at CV ATP hydrolysis and not at ATP hydrolysis by other hydrolyzes? Mitochondria contain a variety of ATP hydrolyses that are not CV.

   To determine the acidification rate produced by other hydrolyses, we inhibit CV by oligomycin. Oligomycin resulted in over 95% inhibition of the rate of acidification. This is in agreement with high abundancy of CV is in the mitochondria as compared to any other protein, including other hydrolyzes.

B. How we assure that acidification is not contributed by carbon dioxide product of respiration or by lactate produced in glycolysis?

Coupled mitochondria can respire and produce CO_2 which forms acid. To assure CO_2 is not a significant source of protons we reduced respiration by shifting ATP/ADP ratio towards higher levels of ATP, thereby producing a stable state 4, in which respiration is inhibited by the product of ATP synthase, ATP. Normally, state 4 pertains to a sample depleted of ADP. Here we created a state 4 ATP, in which ATP concentrations are high enough to slow down respiration.

C. How we assure that acidification is not contributed by lactate produced in glycolysis?

Lactate production through glycolysis requires supply of glucose and glycolytic enzymes, including lactate dehydrogenase. Isolated mitochondria preps do not have a supply of glucose and the cytosolic enzyme lactate dehydrogenase is removed during the purification of the mitochondria.

D. How we address the contribution of proton leak to hydrolysis?

Proton leak is not sufficient to induce ATP hydrolysis. It is the proton motive force that has to reverse itself due to depolarization. Depolarization will stimulate respiration. Our results show that the addition of ATP did not result in an increase in respiration. To the contrary, it resulted in further inhibition of respiration.

REFERENCES

Acin-Perez R, Benador IY, Petcherski A, Veliova M, Benavides GA, Lagarrigue S, Caudal A, Vergnes L, Murphy AN, Karamanlidis G, Tian R, Reue K, Wanagat J, Sacks H, Amati F, Darley-Usmar VM, Liesa M, Divakaruni AS, Stiles L, Shirihai OS (2020) A novel approach to measure mitochondrial respiration in frozen biological samples. EMBO J 39: e104073

Desousa B.R., Kim K.K.O., Hsieh W.Y., Jones A.E., Swain P., Morrow D.H., Ferrick D.A., Shirihai O.S., Neilson A., Nathanson D.A., Rogers G.W., Dranka B.P., Murphy A.N., Affourtit C, Bensinger S.J., L. S, Romero N., A.S. D (2022) Calculating ATP production rates from oxidative phosphorylation and glycolysis during cell activation.

Divakaruni AS, Hsieh WY, Minarrieta L, Duong TN, Kim KKO, Desousa BR, Andreyev AY, Bowman CE, Caradonna K, Dranka BP, Ferrick DA, Liesa M, Stiles L, Rogers GW, Braas D, Ciaraldi TP, Wolfgang MJ, Sparwasser T, Berod L, Bensinger SJ et al. (2018) Etomoxir Inhibits Macrophage Polarization by Disrupting CoA Homeostasis. Cell Metab 28: 490-503 e7

Divakaruni AS, Paradyse A, Ferrick DA, Murphy AN, Jastroch M (2014) Analysis and interpretation of microplate-based oxygen consumption and pH data. Methods Enzymol 547: 309-54

Fernandez-del-Rio L., Benincá C., Villalobos F., Shu C., Stiles L., Liesa M., Divakaruni A.S., Acin-Perez R., O.S. S (in press) A Novel Approach to Measure Complex V ATP Hydrolysis in Frozen Cell Lysates and Tissue Homogenates. Life Science Alliance

McQuaker SJ, Quinlan CL, Caldwell ST, Brand MD, Hartley RC (2013) A prototypical small-molecule modulator uncouples mitochondria in response to endogenous hydrogen peroxide production. Chembiochem 14: 993-1000

Sarkar P, Chattopadhyay A (2017) Solubilization of the serotonin(1A) receptor monitored utilizing membrane dipole potential. Chem Phys Lipids 209: 54-60
