# Supplementary material for: Inhibition of ATP synthase reverse activity restores energy homeostasis in mitochondrial pathologies
Source: EMBO J. 2023 Mar 13;42(10):e111699. doi: 10.15252/embj.2022111699 (PMC10183817; doi:10.15252/embj.2022111699)
Supplement: Supplementary file 2 — Expanded View Figures PDF [file EMBJ-42-e111699-s008.pdf]

Expanded View Figures

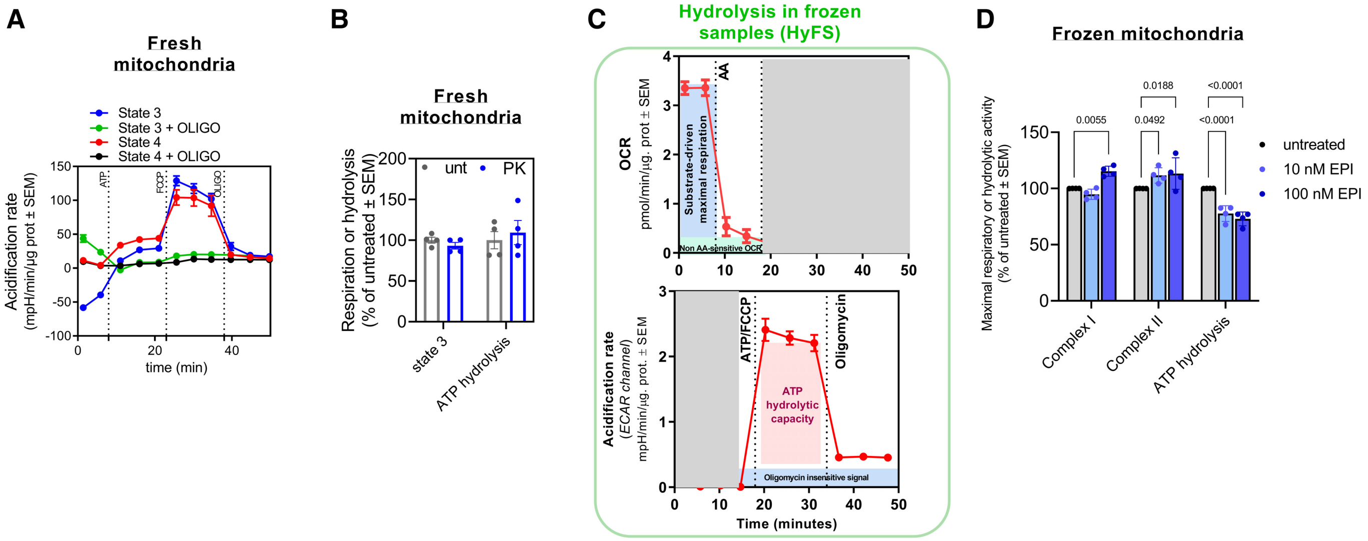

Figure EV1. ATP hydrolysis can be measured in fresh and frozen mitochondria (linked to main Fig 1). Also, see Appendix Supplementary Methods.

A Representative acidification rate traces of heart mitochondria respiring with the indicated substrates either in State 4 (no ADP) or State 3 (plus ADP) fueled by Pyruvate + Malate.

B State 3 and ATP hydrolytic activity in fresh heart mitochondria untreated or treated with proteinase K (PK) (n = 4).

C Overview of the ATP hydrolysis assay (HyFS). Representative trace of OCR (top) of a sample measured with the substrates of interest. Representative acidification rate (ECAR channel) trace from the same assay (bottom).

D Effects of increasing concentrations of EPI in maximal CI, CII and ATP hydrolytic activity measured in frozen mouse heart mitochondria (n = 4).

Data information: Each point represents a biological sample replicate. For each biological replicate, technical replicates were averaged. Data represent average ± SEM. Two-way ANOVA followed by Šídák's multiple comparisons test shows statistical differences depicted by P-value.

 © 2023 The Authors

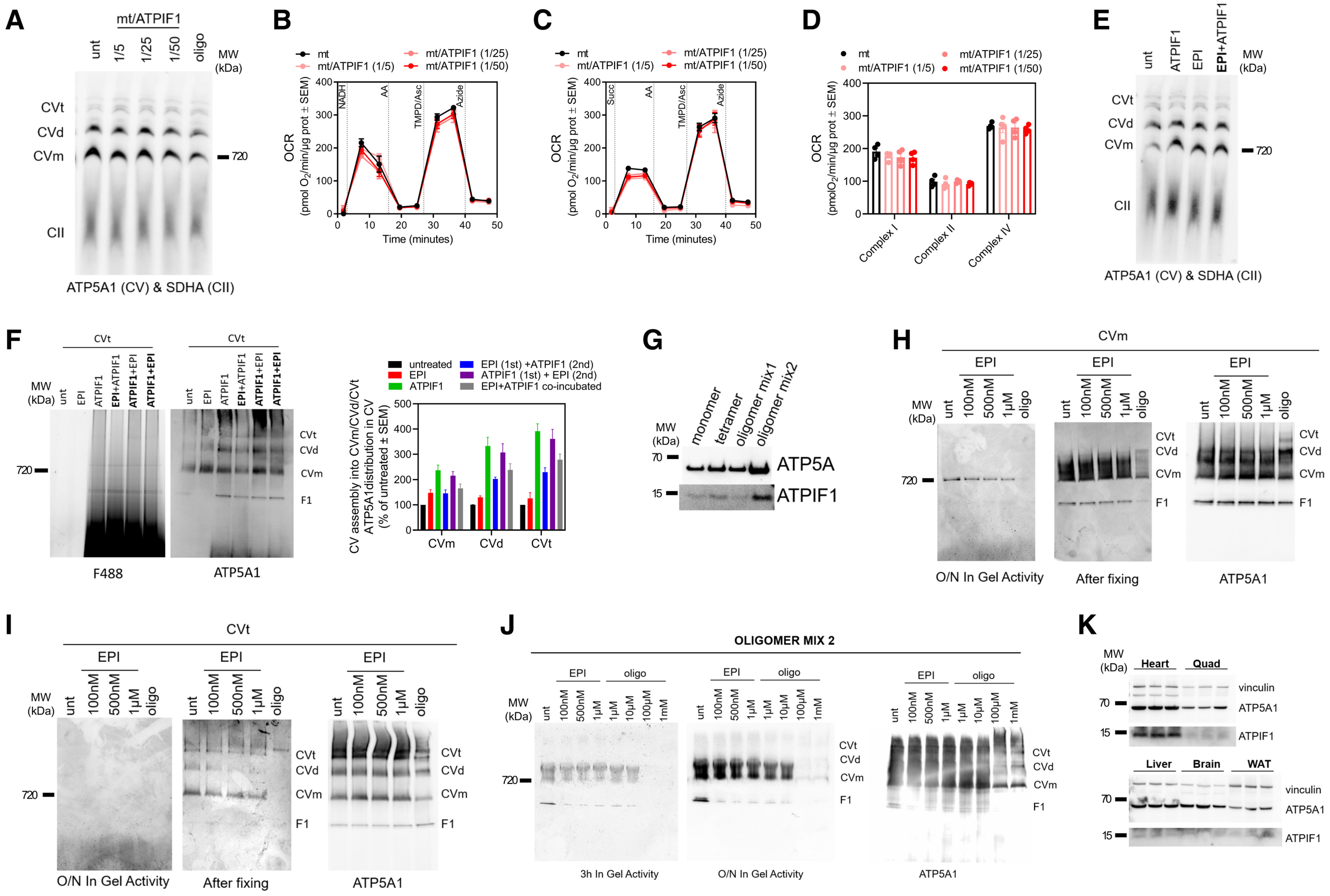

Figure EV2. EPI binds to ATPIF1 pocket in CV (linked to main Fig 3).

A CV assembly in heart mitochondria incubated in the presence of different concentrations of ATPIF1-GFP. CII (SDHA) was used as loading control.

B–D Representative Seahorse traces showing the effects of increasing concentrations of ATPIF1-GFP added to mitochondria on maximal CI, CII and CIV (B, C) activity measured in frozen mouse heart mitochondria and its quantification (D) ($n = 4$).

E CV assembly in heart mitochondria incubated with ATPIF1, EPI or both. CII (SDHA) was used as loading control.

F Representative blots showing the competition assay between ATPIF1-GFP and EPI for binding to CV tetramer (CVt) (left) and their quantification (right).

G Expression levels of ATP5A1 and ATPIF1 in the indicated bovine CV preparations.

H–J CV in gel ATP hydrolytic activity from purified bovine CV preparations under the indicated EPI and oligo concentrations; CV monomer (H) CV tetramer (I), and oligomer mix 2 (J). CVm: CV monomer; CVt: CV tetramer. CV in gel activity is measured after O/N incubation or 3h (J) (left blot) and after stopping the activity with 50% methanol (middle blot). Western blot for ATP5A1 was used as loading control (right blot).

K Expression levels of ATP5A1 and ATPIF1 in mouse tissue lysates. Vinculin is used as loading control.

Data information: For each biological replicate, technical replicates were averaged. Data represent average ± SEM.

© 2023 The Authors

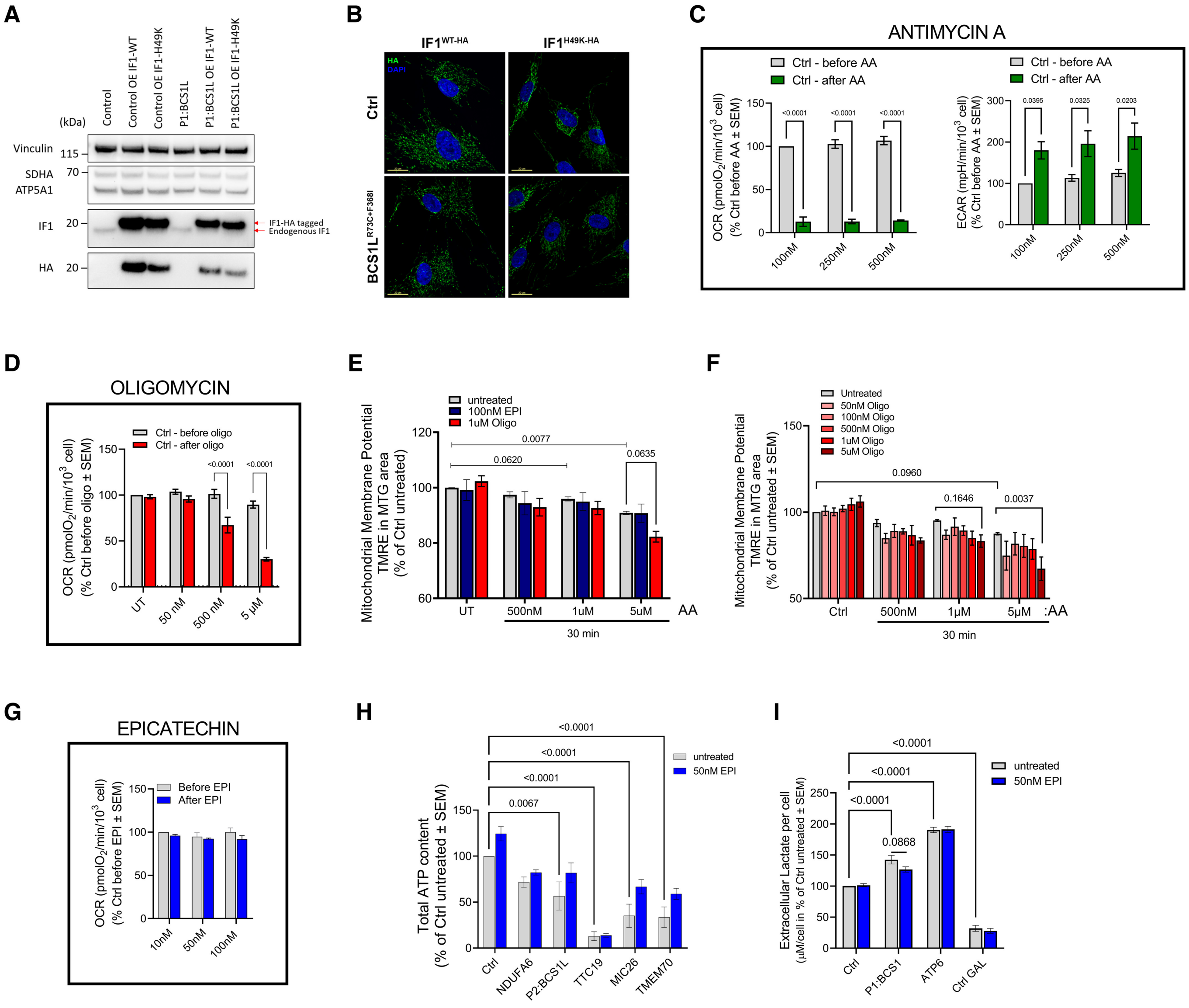

Figure EV3. Screening of EPI effect and inhibition of ATP hydrolysis in mitochondrial disease models (linked to main Fig 4).

A Representative SDS-PAGE blots for ATPIF1, HA, SDHB, ATP5A, and Vinculin showing the overexpression of ATPIF1-WT and H49K in Control (Ctrl) and CIII-deficient cells (P1:BCS1L).

B Representative confocal images showing mitochondrial localization of overexpressed ATPIF1-WT and H49K in Ctrl and CIII-deficient fibroblasts, labeled with anti-HA (green) antibody and DAPI (nuclei – blue). Maximum intensity projection is shown. Scale bars: 20 μm.

C Basal respiration (left) and acidification rates (right) in control fibroblasts before and after the direct injection of the indicated concentrations of Antimycin A (AA) (n = 4).

D Basal respiration in control fibroblasts before and after the direct injection of the indicated concentrations of oligomycin.

E, F Membrane potential measured using TMRE (average intensity) in Mitotracker Green (mitochondria area) normalized per % of untreated control: (E) control fibroblasts incubated for 30 min with AA plus DMSO, 100 nM EPI or 1 μM Oligo (n = 3); and (F) control fibroblasts incubated for 30 min with AA plus increased concentrations of Oligomycin (Oligo) (n = 3).

G Basal respiration (OCR) in control fibroblasts before and after the direct injection of the indicated concentrations of EPI (n = 4).

H Total ATP content measured in fibroblasts from patients with different mutations in mitochondrial proteins by luciferase assay and normalized by % of luminescence from control cells (n = 3).

I Extracellular lactate levels measured as % of the untreated control from Control (Ctrl), CIII-(P1:BCS1L) and CV-deficient (ATP6) fibroblasts treated with and without 50 nM EPI (n = 3). Control cells grown in galactose media (GAL) were included as a positive control for low levels of lactate.

Data information: In all cases, data represent average ± SEM. Two-way ANOVA followed by Šídák's multiple comparisons test shows statistical differences depicted by P-value.

 © 2023 The Authors

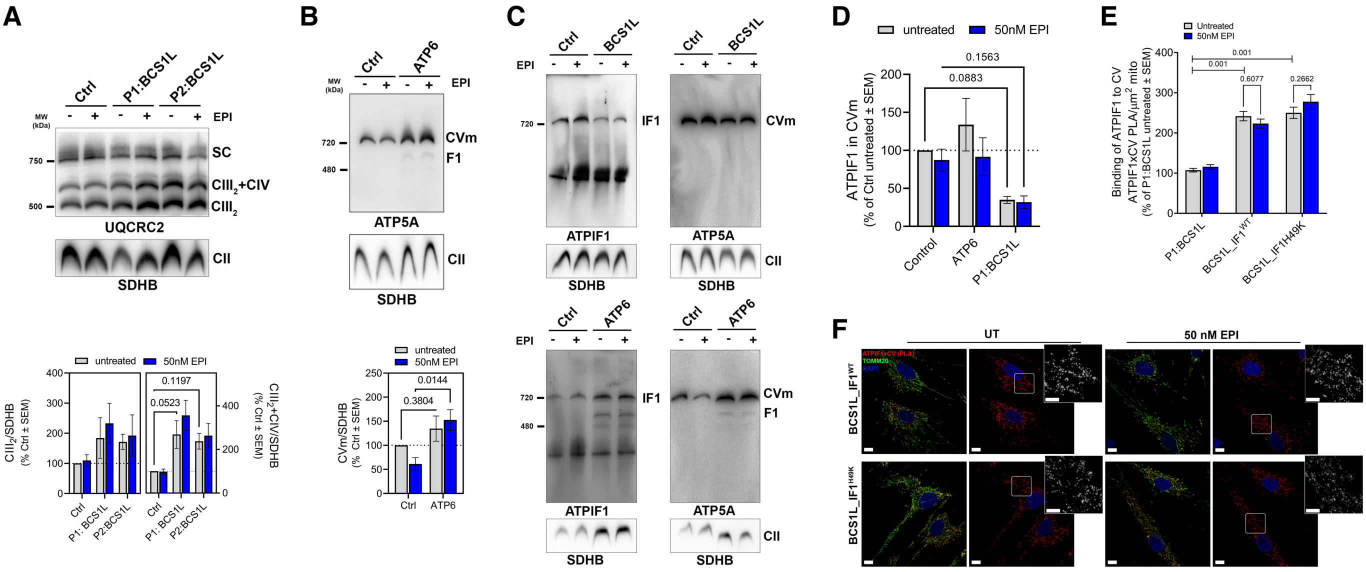

Figure EV4. EPI replaces ATPIF1 to block ATP hydrolysis in mitochondrial disease models (linked to main Fig 5).

A Representative BNGE blots (top) and quantification (bottom) of $CIII_2$, $CIII_2$ + CIV showing the distribution of CIII complexes and supercomplexes in control and CIII-deficient cells treated with and without 50 nM EPI for 24 h. Samples were immunoblotted with UQCRC2 antibody. SDHB was used as a loading control.

B Representative BNGE blots (top) and quantification (bottom) of CV monomer (CVm) supercomplex in control and CV-deficient cells treated with and without 50 nM EPI for 24 h. Samples were immunoblotted with ATP5A antibody. SDHB was used as a loading control.

C, D (C) Representative BNGE blots and quantification (D) of ATPIF1 relative intensity normalized per amount of CV and represented as % of control untreated cells in fibroblasts (n = 3). SDHB was used as a loading control.

E Chart shows PLA dots/μm^3 of mitochondria in fibroblasts normalized as % of Ctrl values (n = 2).

F Representative confocal images showing fibroblasts treated with EPI for 24 h, labeled with anti-ATPIF1 and anti-ATP5A1 (PLA in red) and anti-TOMM20 (green) antibodies. Maximum intensity projection is shown. Scale bars: 20 and 5 µm.

Data information: In all cases, data represent average ± SEM. Two-way ANOVA followed by Šídák's multiple comparisons test shows statistical differences depicted by P-value.

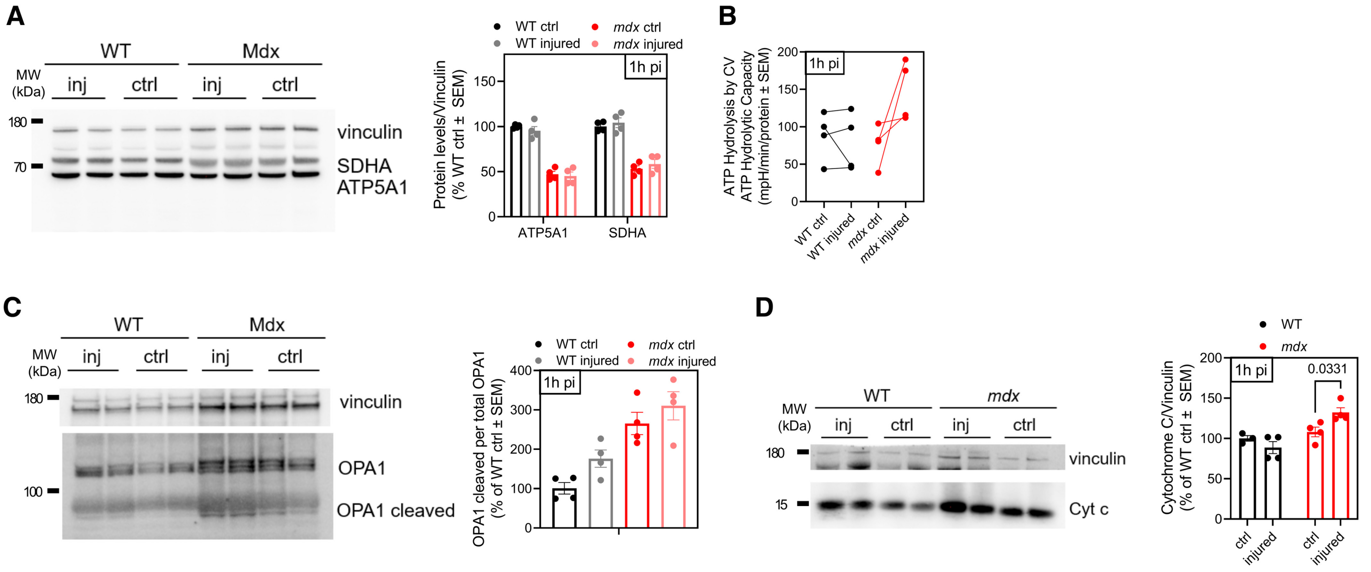

Figure EV5. ATP hydrolysis is increased in *mdx* eccentric injury model 1 h postinjury (linked to main Fig 6).

A Representative western blot (right) and quantification (left) showing CV (ATP5A1) and CII (SDHA) levels in gastrocnemius homogenate in WT and *mdx* mice 1 h after eccentric injury. Vinculin was used as loading control ($n = 4$).

B Maximal ATP hydrolysis capacity per total CV measured in frozen gastrocnemius homogenate in WT and *mdx* mice after 1 h of eccentric injury.

C Analysis of OPA1 levels and isoforms (right) and quantification (left) in gastrocnemius homogenate in WT and *mdx* mice after 1 h of eccentric injury.

D Cytochrome c release in gastrocnemius supernatants of WT and *mdx* 1 h postinjury measured by western blot. From (A–D), $n = 4$.

Data information: For each biological replicate, technical replicates were averaged. Data represent average ± SEM. Two-way ANOVA followed by Šídák's multiple comparisons test shows statistical differences depicted by *P*-value.

 © 2023 The Authors

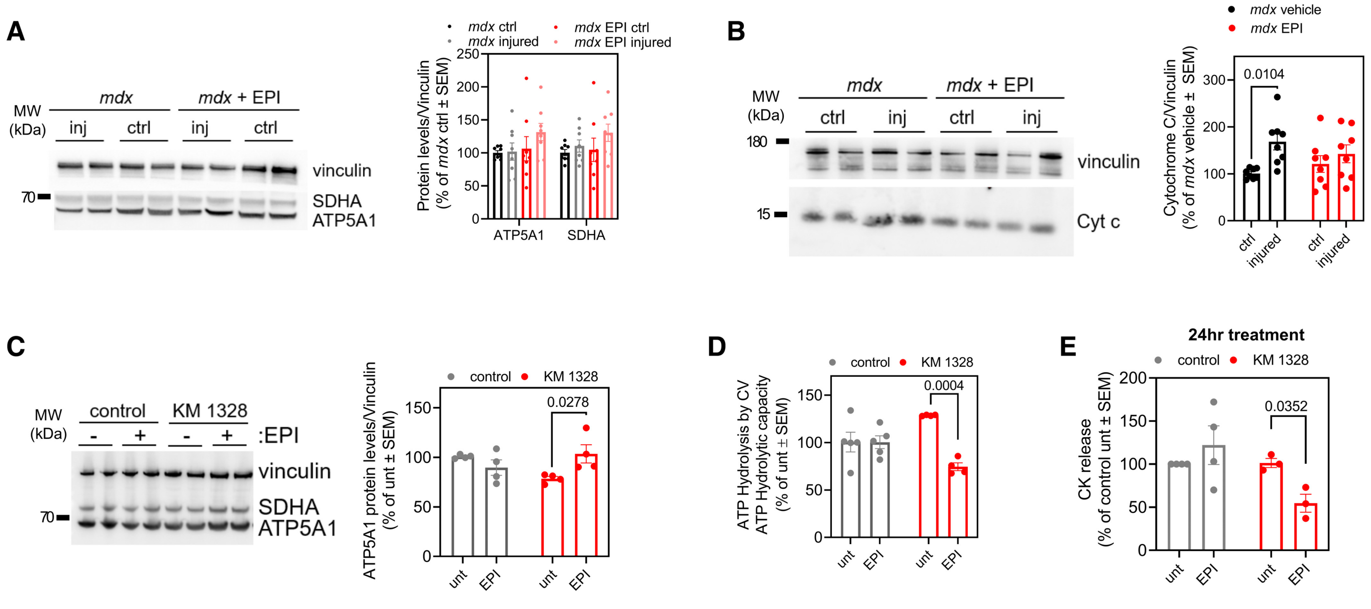

Figure EV6. *In vivo* and *in vitro* ATP hydrolysis inhibition by EPI in *mdx* mice and DMD cell lines (linked to main Fig 7).

A Representative western blot showing CV (ATP5A1) and CII (SDHA) levels in gastrocnemius homogenate in *mdx* vehicle or EPI treated 24 h after eccentric injury (left). Quantification of the protein levels (right). Vinculin was used as loading control (n = 8).

B Cytochrome c release in gastrocnemius supernatants of *mdx* vehicle or EPI treated 24 h after eccentric injury measured by western blot (n = 8). Quantification of the protein levels (right).Vinculin was used as loading control (n = 8).

C Representative western blot showing CV (ATP5A1) and CII (SDHA) levels in DMD cell lines after 24 h treatment with 50 nM EPI (left). Quantification of the protein levels versus vinculin versus control untreated (right). Vinculin was used as loading control ($n \geq 3$).

D Maximal ATP hydrolytic capacity in myotubes normalized by CV levels after 24 h of treatment with vehicle or 50 nM EPI ($n \geq 3$).

E Cell membrane stability as measured by creatine kinase (CK) release in myotubes treated 24 h with vehicle or 50 nM EPI ($n \geq 3$).

Data information: Each point represents a biological replicate. For each biological replicate, technical replicates were averaged. Data represent average ± SEM. Two-way ANOVA followed by Šídák's multiple comparisons test shows statistical differences depicted by *P*-value.

© 2023 The Authors
