# Supplementary material for: Inhibition of ATP synthase reverse activity restores energy homeostasis in mitochondrial pathologies
Source: EMBO J. 2023 Mar 13;42(10):e111699. doi: 10.15252/embj.2022111699 (PMC10183817; doi:10.15252/embj.2022111699)
Supplement: Supplementary file 4 — PDF+ [file EMBJ-42-e111699-s001.pdf]

Article

SOURCE DATA TRANSPARENT PROCESS OPEN ACCESS

THE EMBO JOURNAL

Inhibition of ATP synthase reverse activity restores energy homeostasis in mitochondrial pathologies

Rebeca Acin-Perez[1,2,†], Cristiane Benincá[1,2,†], Lucia Fernandez del Rio[1,2], Cynthia Shu[1,2], Siyouneh Baghdasarian[1,2], Vanessa Zanette[3], Christoph Gerle[4,5], Chimari Jiko[6], Ramzi Khairallah[7], Shaharyar Khan[8], David Rincon Fernandez Pacheco[9], Byourak Shabane[1,2], Karel Erion[10], Ruchi Masand[10], Sundeep Dugar[10], Cristina Ghenoiu[10], George Schreiner[10], Linsey Stiles[1,2,11], Marc Liesa[1,2,11,12,13,*] & Orian S Shirihai[1,2,11,12,**]

Abstract

The maintenance of cellular function relies on the close regulation of adenosine triphosphate (ATP) synthesis and hydrolysis. ATP hydrolysis by mitochondrial ATP Synthase (CV) is induced by loss of proton motive force and inhibited by the mitochondrial protein ATPase inhibitor (ATPIF1). The extent of CV hydrolytic activity and its impact on cellular energetics remains unknown due to the lack of selective hydrolysis inhibitors of CV. We find that CV hydrolytic activity takes place in coupled intact mitochondria and is increased by respiratory chain defects. We identified (+)-Epicatechin as a selective inhibitor of ATP hydrolysis that binds CV while preventing the binding of ATPIF1. In cells with Complex-III deficiency, we show that inhibition of CV hydrolytic activity by (+)-Epichatechin is sufficient to restore ATP content without restoring respiratory function. Inhibition of CV–ATP hydrolysis in a mouse model of Duchenne Muscular Dystrophy is sufficient to improve muscle force without any increase in mitochondrial content. We conclude that the impact of compromised mitochondrial respiration can be lessened using hydrolysis-selective inhibitors of CV.

Keywords ATP hydrolysis; ATPase Inhibitor (ATPIF1); Complex V; epicatechin; muscular dystrophy

Subject Categories Metabolism; Molecular Biology of Disease

DOI 10.15252/embj.2022111699 | Received 19 May 2022 | Revised 24 January 2023 | Accepted 25 January 2023 | Published online 13 March 2023

The EMBO Journal (2023) 42: e111699

See also: **GE Valdebenito *et al*** (May 2023)

Introduction

Mitochondrial ATP synthase (F_1F_0ATPase, Complex V or CV) switches from being an ATP (adenosine triphosphate) producer to an ATP consumer. ATP hydrolysis by CV (CV ATP hydrolysis) is prevented by electrochemical as well as structural elements. The electrochemical element is the proton motive force (PMF), while the structural element is a mitochondrial protein, ATPase Inhibitor (ATPIF1, ATP5IF1, or IF1). The PMF, comprised of the membrane potential ($\Delta\Psi$m) and the pH gradient, drives the clockwise rotation of CV, resulting in the concurrent re-entry of protons and ATP synthesis. A fall in the electrochemical proton gradient and membrane potential depolarization reverses the direction of CV rotation, resulting in ATP hydrolysis. CV reverse rotation results in the extrusion of protons into the intermembrane space, which contributes to the restoration of the proton gradient and the membrane potential across the inner membrane, while at the same time resulting in a net loss of ATP (Chinopoulos & Adam-Vizi, 2010; Rieger *et al*, 2021). Previous studies demonstrated the occurrence of ATP hydrolysis during depolarization due to the arrest of respiratory chain activity or robust uncoupling. Under such

1 Department of Medicine, Endocrinology, David Geffen School of Medicine, University of California, Los Angeles, CA, USA
2 Metabolism Theme, David Geffen School of Medicine, University of California, Los Angeles, CA, USA
3 Department of Bioinformatics, University Federal of Parana, Curitiba, Brazil
4 Institute for Protein Research, Osaka University, Suita, Japan
5 RIKEN SPring-8 Center, Sayo-gun, Japan
6 Institute for Integrated Radiation and Nuclear Science, Kyoto University, Kyoto, Japan
7 Myologica LLC., New Market, MD, USA
8 Gencia Biotech, Charlottesville, VA, USA
9 Board of Governors Regenerative Medicine Institute, Cedars-Sinai Medical Center, Los Angeles, CA, USA
10 Epirium Bio Inc., San Diego, CA, USA
11 Department of Molecular and Medical Pharmacology, University of California, Los Angeles, CA, USA
12 Molecular Cellular Integrative Physiology, University of California, Los Angeles, CA, USA
13 Institut de Biologia Molecular de Barcelona, IBMB, CSIC, Barcelona, Catalonia, Spain
*Corresponding author. Tel: +34 93 4020504; E-mail: mlrbmc@ibmb.csic.es
**Corresponding author. Tel: +1 310 825 5160; E-mail: oshirihai@mednet.ucla.edu.
†These authors contributed equally to this work

© 2023 The Authors. Published under the terms of the CC BY 4.0 license

extreme conditions, evidence of reverse activity of CV could be gathered using oligomycin (Oligo), which blocks CV hydrolysis and synthesis functions. If the respiratory chain can keep the membrane potential intact, oligomycin treatment will hyperpolarize the inner membrane. However, if membrane potential is maintained by the reverse action of ATP synthase, oligomycin will induce depolarization, a phenomenon termed oligomycin null-point (Connolly *et al*, 2018). Oligomycin null-point has been demonstrated in extreme conditions such as hypoxia and complete blockade of respiratory chain activity with antimycin A (AA) (Rego *et al*, 2001; Vesce *et al*, 2005; Nicholls *et al*, 2013; Chen *et al*, 2014). However, whether ATP hydrolysis occurs in coupled respiring mitochondria has not been demonstrated.

A recent study demonstrates that the membrane potential along the inner membrane is heterogeneous. Some cristae have more depolarized membrane potential compared with others, while the inner boundary membrane, in between the cristae, has lower membrane potential (Wolf *et al*, 2019). Other studies show that CV can be found leaving the cristae and moving to the inner boundary membrane (Weissert *et al*, 2021). These two observations together confirm that the electrochemical conditions required for ATP hydrolysis are met more frequently than appreciated before and may take place in normal or mildly compromised mitochondria, such as those occurring in humans with mitochondrial genetic diseases and myopathies.

As the structural element preventing ATP hydrolysis, ATPIF1 binds to the F1 head of the ATP synthase, between the α and β subunits' α-helices. The ATPIF1 N-terminal region reaches the γ subunit of the central stalk, thereby preventing the complete rotation of ATP synthase (Gledhill *et al*, 2007). The blockage of rotation is consistent with data supporting that ATPIF1 blocks both CV–ATP synthesis and hydrolysis (Garcia-Bermudez & Cuezva, 2016). While ATPIF1 is an efficient inhibitor of CV–ATP hydrolysis, its expression and binding vary dramatically across tissues and in response to disease, and thus, the functional level of inhibition is not possible to predict.

Given that the electrochemical and structural elements that permit CV–ATP hydrolysis are too complex to derive a prediction of the rate of ATP hydrolysis in cells and tissues, the impact of CV–ATP hydrolysis on cellular energetics has not been determined. Moreover, in conditions of impaired respiratory function, the relative impact of impaired ATP synthesis and increased ATP hydrolysis on the depletion of cellular ATP and on the pathology has not been determined.

Here we provide the first measurements of CV–ATP hydrolysis in coupled mitochondria to demonstrate that ATP hydrolysis occurs in healthy mitochondria, supporting that both the electrochemical and structural elements required for hydrolysis to occur exist in coupled healthy mitochondria. We identified (+)-Epicatechin (EPI) as an inhibitor of CV–ATP hydrolysis that binds CV, with a predicted binding site inside the ATPIF1 binding groove of CV.

We show that EPI selectively decreases CV–ATP hydrolysis activity without affecting ATP synthesis. Using EPI, we find that inhibition of CV–ATP hydrolysis can increase cellular ATP content without repairing respiratory function. In a muscle dystrophy model, we show that the level of decline in muscle force directly correlates to the level of CV hydrolysis and that inhibition of hydrolysis can improve muscle force.

Results

A novel assay quantifies proportion of ATP synthase working in forward and in reverse in healthy, coupled mitochondria

ATP hydrolysis by CV is thought to only occur when mitochondria depolarize. However, given the differences in membrane potential between individual cristae and between cristae and inner boundary membrane within each mitochondrion (Wolf *et al*, 2019), the question of whether hydrolysis can occur concurrently with synthesis arises. To develop an assay for the measurement of ATP hydrolysis, we took advantage of the proton generated as part of the chemical reaction of ATP hydrolysis (see Appendix Supplementary Methods). The proton produced during this reaction can be measured as the acidification rate of the sample. If membranes are intact, as in fresh coupled mitochondria, protons will leave the mitochondria as part of the reverse activity of CV. When ATP synthase works in reverse, one proton is net transported per 2.67 molecules of ATP hydrolyzed (Hochachka & Mommsen, 1983; Robergs *et al*, 2004; Ferguson, 2010). To concurrently measure ATP synthesis and hydrolysis in the same sample, we used the capacity of the Seahorse (SH) XF96 Analyzer to measure oxygen consumption and pH changes (acidification rates) in different channels. ATP synthesis is determined by measuring oxygen consumption induced by saturating concentrations of adenosine diphosphate (ADP) (State 3 respiration), while ATP hydrolysis is quantified by measuring acidification via the protons released by CV when hydrolyzing ATP (Fig 1A and B). Since we are using isolated mitochondria, the changes in pH cannot be attributed to glycolysis (Divakaruni *et al*, 2014), but to proton release by CV hydrolysis.

We isolated intact mitochondria from mouse heart and measured oxygen consumption and proton release in fresh preparations. Measurements were carried out either in the presence of substrates and ADP (respiration linked to ATP synthesis or State 3) or in the presence of substrates without ADP (respiration linked to proton leak or State 4).

The substrates fueling respiration were pyruvate plus malate (Pyr + Mal, Fig 1B and C). The procedure starts with the addition of ADP, stimulating State 3 respiration. A control group, that remains in State 4 without the addition of ADP shows low oxygen consumption rates (OCR), confirming that the mitochondria are coupled. State 3 respiration slowly depletes ADP, leading to State 4 where OCR rates are lower. At this point, we added ATP generating the "State 4 ATP" condition. Remarkably, the addition of ATP resulted in a sharp increase in proton release that was oligomycin sensitive, confirming its origin from ATP hydrolysis and indicating that ATP hydrolysis occurs in coupled mitochondria (Figs 1B and D, and EV1A).

To verify that acidification was a result of CV reverse activity, we depolarized the same mitochondria with carbonyl cyanide p-(trifluoromethoxy)phenyl-hydrazone (FCCP), which reverses all CV molecules, and observed an additional increase in acidification (Fig 1B and D, Also, see Appendix Supplementary Methods). Furthermore, oligomycin, a specific inhibitor of forward and reverse CV activities, decreased acidification (Fig 1B and D), which effectively demonstrated that indeed CV activity was the major determinant of proton release in FCCP-treated mitochondria (Divakaruni *et al*, 2014). This acidification induced by ATP was not a result of the presence of damaged mitochondria, as the mitochondrial

 © 2023 The Authors

preparations were highly coupled, demonstrated by their high State 3/State 4 OCR ratio. Additionally, we further investigated whether an unexpected presence of broken mitochondria or extramitochondrial ATPases contributed to the acidification detected. Since exposed ATPases and mitochondria with broken membranes are sensitive to proteinase K (PK) treatment, we measured State 3 OCR and acidification rates after ATP injection in the presence of PK. Addition of PK did not change respiration nor acidification, confirming that protons were released from CV of intact mitochondria (Fig EV1B and Appendix Fig S1).

The injection of ATP increased acidification rates of mitochondria respiring under State 3 or State 4 (Figs 1D and EV1A). The observation of a similar acidification response to ATP injection under either State 3 or State 4 indicated that ATP hydrolysis can be detected in the presence of high-ADP/ATP exchange activity, as the latter can only occur in State 3 mitochondria (State 3 mitochondria have ADP in the matrix prior to ATP injection).

Coupled mitochondria keep a PMF that is expected to prevent ATP hydrolysis by CV. However, the recent discovery that microdomains within the mitochondrial inner membrane have reduced PMF, provided a scenario for hydrolysis to still occur in coupled mitochondria. The assay described here provides a direct measurement of this phenomenon.

Based on these results, we conclude that a population of CV molecules in coupled mitochondria can hydrolyze ATP, while others can produce ATP. The rate of ATP hydrolysis in coupled mitochondria was approximately 20% of the maximal hydrolytic capacity measured in mitochondria uncoupled with FCCP. This percentage suggests that the PMF is responsible for preventing 80% of CV hydrolytic capacity in coupled mitochondria.

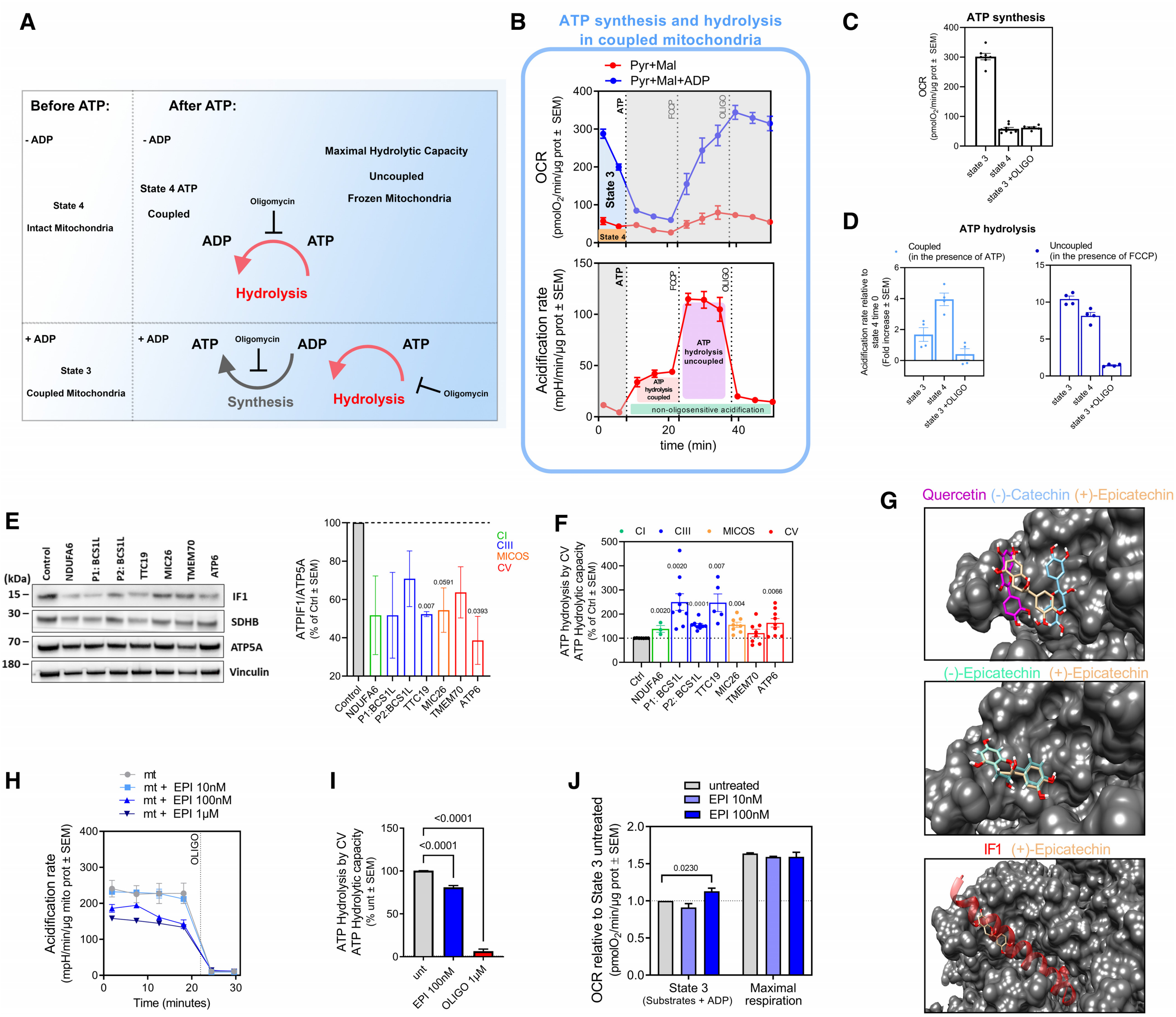

Figure 1.

Figure 1. ATP synthesis and hydrolysis coexist in the same mitochondria and can be modulated independently (see also Fig EV1 and Dataset EV1).

A Scheme representing the forward (synthesis) and reverse (hydrolysis) mode of ATP synthase and the analysis that can be performed in fresh (intact) or previously frozen samples.

B Sequential measurements of respiration and ATP hydrolysis in freshly isolated heart mitochondria. The top (OCR) and bottom (Acidification) charts were recorded simultaneously from the same sample. ATP hydrolysis is measured using the acidification channel (bottom). Oxygen consumption rate (OCR) is measured first in the presence of ADP and fueled by pyruvate (Pyr) and malate (Mal) (Blue; State 3 respiration), ADP is phosphorylated, and oxygen is consumed. A control group received pyruvate and malate alone (red; State 4), showing OCR that were lower than State 3, demonstrating that mitochondria are coupled. Following the addition of ATP, OCR remains low, but hydrolysis measured by acidification is increased. Hydrolysis is further activated by FCCP-induced depolarization and inhibited by oligomycin. The oligomycin-insensitive portion of acidification is attributed to carbon dioxide-induced respiration.

C OCR of heart mitochondria respiring using Pyr + Mal in State 4 (no ADP), State 3 (presence of ADP) or State 3 plus oligomycin ($n \geq 6$).

D ATP hydrolysis measured as acidification rates in heart mitochondria respiring in State 4 (no ADP), State 3 (presence of ADP) or State 3 plus oligomycin using Pyr + Mal under conditions of coupled (in the presence of ATP) or uncoupled (in the presence of FCCP) ($n = 4$). Note that ATP hydrolysis in coupled mitochondria is at steady state at 20% of the maximal ATP hydrolytic capacity measured in uncoupled mitochondria.

(E–F) Screening for mutations that result in increased ATP hydrolysis by CV. (E) Representative SDS-PAGE blots of ATPIF1, SDHB, ATP5A1, and Vinculin in control and mutant patient-derived fibroblasts. Right panel shows mean of ATPIF1 bands relative intensity normalized per ATP5A1 and represented as % of control cells ($n = 2$). The colored histogram illustrates the mitochondrial element impacted by each mutation. Note that ATPIF1 expression in patient cells is lower than in cells from control subjects. (F) ATP hydrolytic capacity measured in control and mutant patient derived fibroblasts ($n \geq 3$). Data are normalized by the maximal CII activity (SR respiration) of the samples. Colored histogram illustrates the mitochondrial element impacted by each mutation. Note that ATP hydrolytic capacities in cells from specific patients are higher than those found in control subjects.

G Identification of binding sites in the ATPIF1 binding groove on the surface of F1-ATP synthase (1ohh, gray) using PELE. Image shows binding sites for Quercetin and the enantiomers, (-)-Catechin and (+)-Epicatechin (top); (-)-Epicatechin versus (+)-Epicatechin (middle) and (+)-Epicatechin and ATPIF1 (bottom).

H Representative Seahorse profiles measuring ATP hydrolytic activity (millipH units, mpH) in isolated heart mitochondria in the presence of the indicated concentrations of EPI, using oligomycin (OLIGO) as an inhibitory control.

I EPI reduces maximal hydrolytic capacity of CV. Maximal ATP hydrolysis capacity measured in frozen heart mitochondria in the presence of EPI (100 nM) and using oligomycin (1 μM) as control ($n \geq 7$).

J State 3 and maximal respiration driven by pyruvate + malate in isolated mitochondria from mouse heart. EPI was added to the respiration media at the indicated concentrations ($n \geq 3$). Note that EPI does not affect maximal respiration but has a small effect on ATP synthesis-dependent OCR.

Data information: For each biological replicate, technical replicates were averaged. Data represent average ± SEM. Unpaired *t*-test versus Ctrl or untreated conditions shows statistical differences depicted by *P*-value.
Source data are available online for this figure.

ATPIF1 downregulation and increased CV–ATP hydrolysis is commonly observed in mitochondrial disease models

ATPase inhibitor (ATPIF1) binds CV and regulates ATP hydrolysis and synthesis (Nesci *et al*, 2015). Considering that ATPIF1 expression levels vary by tissue and environmental conditions (Campanella *et al*, 2008, 2009), we hypothesized that decreased ATPIF1 expression levels could play a role in the pathophysiology of mitochondrial disease models. We quantified ATPIF1 levels in skinfibroblasts derived from patients with a variety of mitochondrial diseases. Fibroblasts with mutations causing dysfunction in Complex I (CI; NDUFA6), Complex III (CIII; BCS1L and TTC19), ATP synthase (CV; TMEM and ATP6), and MICOS (MIC26) were analyzed by western blot. We observed a decrease in total ATPIF1 protein levels in all mutants tested (Fig 1E), suggesting that increased levels of ATP hydrolysis could be a shared pathogenic process among patients with mitochondrial diseases. If a reduction in ATPIF1 translates to increased ATP hydrolysis, we would expect that the maximal hydrolytic capacity per CV content would increase.

To quantify ATP hydrolytic capacity in cells, we developed an approach to measure maximal ATP hydrolysis by CV in samples that were previously frozen (Fig EV1C) named Hydrolysis in Frozen Samples or HyFS (Fernandez-del-Rio *et al*, 2023). An increase in maximal CV–ATP hydrolytic capacity was observed in CIII mutants (BCS1L and TTC19), MICOS-deficient patients (MIC26), and a CV mutant (ATP6) (Fig 1F).

These data show that ATPIF1 downregulation together with increased ATP hydrolytic capacity is a mitochondrial phenotype shared among human fibroblasts from patients with mutations in mitochondrial proteins.

In silico identification of CV–ATP hydrolysis inhibitors

Our results in mitochondrial disease cell models highlight the need for pharmacological interventions to modulate CV hydrolytic activity. The identification of compounds selectively decreasing CV hydrolytic activity would allow us to establish whether the increase in ATP hydrolysis observed in fibroblasts from patients with mutations in mitochondrial proteins is an adaptive or maladaptive event. To find possible molecules that selectively regulate CV hydrolytic activity, we performed an *in-silico* screen (Dataset EV1) of 7,441 natural or synthetic derivative compounds from the COlleCtion of Open Natural ProdUcTs database (CoconutDB) (Sorokina *et al*, 2021). Compounds were categorized for predicted desired properties including free radical scavenging activity (antioxidant) and creatine kinase (CK) inhibition, as well as for docking to ATP synthase. From these, 6,827 ligands were filtered out for predicted undesired activities. Since ATPIF1 binds to the F_1 head of ATP synthase, we narrowed the search by analyzing the ability of the remaining 614 ligands to directly dock with the F_1 head. Within the resulting 445 molecules that were found to have at least −8.5 Kcal/Mol affinity to the selected region, we found a group of 28 compounds belonging to the family of catechins that had a particularly high affinity to the F_1 head.

Binding site exploration simulations were performed with and without ATPIF1 using the Protein Energy Landscape Exploration (PELE) webserver (Fig 1G). We ran this analysis using the ligands identified in the CoconutDB study and included (+)-Epicatechin, a catechin derivative shown to protect from myocardial ischemia/reperfusion injury (Yamazaki *et al*, 2008, 2010) by preserving mitochondrial function (Yamazaki *et al*, 2014). PELE identified a low-energy binding site for Quercetin, (-)-Catechin, and (+)-Epicatechin in the vicinity of the ATPIF1 binding groove on the surface of the F_1

 © 2023 The Authors

head. The three compounds bound with similar affinities: AutoDock Vina binding score: −5.3 for (+)-Epicatechin, −5.7 for Quercetin and −5.5 for (-)-Catechin (Fig 1G, top panel).

When comparing the different epicatechin isomers, the resulting pose of (+)-Epicatechin indicated that the B-ring catechol hydroxyls form hydrogen bonds with the side chains of E454 and Q456, and the A-ring resorcinol hydroxyl forms hydrogen bonds with the side chain of H451. The remaining interactions were hydrophobic in nature, with the dihydropyran heterocycle and B-ring benzyl near L452 and P453 that form the ATPIF1 binding groove. The 3-OH of (+)-Epicatechin is positioned away from the surface unlike the same group in (-)-Epicatechin, which sterically hinders the interaction (Fig 1G, middle panel). Finally, our data indicated that ATPIF1 and (+)-Epicatechin align to the same position and orientation in the ATPIF1 binding groove (Fig 1G, bottom panel).

Our results suggest that epicatechin could mimic ATPIF1 and decrease CV-mediated ATP hydrolysis (Lang & Racker, 1974; Gledhill *et al*, 2007).

(+)-Epicatechin (EPI) decreases the ATP hydrolytic capacity of CV, without affecting ATP synthesis capacity or respiration

Based on the predicted high-affinity binding, we chose to further explore the effect of EPI on CV hydrolysis. HyFS analysis in isolated heart mitochondria confirmed that EPI inhibits CV–ATP hydrolysis at a rate of 30% of the maximal ATP hydrolytic capacity. As a positive control, we used oligomycin, which inhibits ATP hydrolysis completely (100%) (Fig 1H and I). To explore EPI specificity, we tested the effect of EPI on electron transport chain (ETC) complexes. EPI did not inhibit respiration on either Complex I (CI) or Complex II (CII) substrates (Fig EV1D). This is in contrast to Quercetin, which has been reported to promote changes in oxidative respiration and redox status (de Oliveira *et al*, 2016). We next tested whether EPI could modulate respiration driven by CV–ATP synthetic activity (ATP-linked respiration). EPI at 100 nM promoted a slight increase in State 3 respiration driven by Pyr + Mal without changing maximal respiration rates (MRRs) (Fig 1J). An increase in State 3 respiration that is not accompanied by an increase in MRR or proton leak (Appendix Fig S2), supports our interpretation that EPI specifically acts on ATP synthase.

(+)-Epicatechin directly binds to CV and decreases its ATP hydrolytic capacity

To determine whether the effects of EPI on ATP hydrolysis were mediated by a direct action on the ATP synthase as the docking data supported, we employed different approaches where EPI was added either to intact mitochondria, broken mitochondria or directly to the gel loaded with mitochondria (Fig 2). First, we treated intact mitochondria with increasing concentrations of EPI. These intact mitochondria treated with EPI were then analyzed by blue native gel electrophoresis (BNGE), to determine whether EPI modulated ATP synthase assembly and in-gel hydrolytic activity (Acin-Perez *et al*, 2020a). EPI decreased in gel ATP hydrolytic activity in a dose-dependent manner (Fig 2A and B), without altering the assembly of CV (Fig 2C). These results show that EPI inhibition of ATP hydrolysis does not involve disruption of CV assembly. In gel activity allows us to specifically determine that the ATP hydrolytic activity measured is a product of fully assembled CV.

To assess if EPI needs to access the mitochondrial matrix in order to inhibit ATP hydrolysis or whether EPI can bind directly to CV moieties in the intermembrane space, we added EPI to mitochondria permeabilized with digitonin, prior to protein separation by BNGE. EPI inhibited hydrolysis in permeabilized mitochondria with the same efficacy as observed in intact mitochondria (Fig 2D and E). Thus, we can conclude that the effect of EPI is not limited by the rate of its diffusion into the mitochondrial matrix.

To assess whether the actions of EPI on CV were caused by direct interaction of EPI with CV, we added EPI in the ATP hydrolysis reaction buffer in which the blue native gel was incubated. In this way, EPI is incubated directly with the separated CV located in the gel. We found that in-gel treatment by EPI decreased ATP hydrolysis (Fig 2F and G). Note that higher concentrations of EPI were needed to penetrate the gel and block hydrolysis. These data suggest that EPI directly binds CV to decrease ATP hydrolytic activity, rather than by modulating an endogenous CV regulator.

(+)-Epicatechin prevents the binding of ATPIF1 to CV

Given the ability of EPI to mimic ATPIF1 function of blocking CV–ATP hydrolysis and given the predicted binding site of EPI in the ATPIF1-binding pocket, we hypothesized that EPI could be preventing ATPIF1 from binding CV. To test this hypothesis, we measured the binding of a recombinant purified ATPIF1-green fluorescent protein (GFP) protein to CV in the presence and absence of EPI. We permeabilized mitochondria by freeze–thaw cycles to enable the direct access of purified ATPIF1-GFP to CV. We then incubated these permeabilized mitochondria with increasing concentrations of ATPIF1-GFP, allowing ATPIF1-GFP to bind CV. The free and CV-bound ATPIF1-GFP were then separated by BNGE. Gels were then excited at 488 nm and binding was then quantified as the levels of green fluorescence emitted by ATPIF1-GFP from the CV band in the BNGE. Quantification shows that ATPIF1-GFP binds CV in a dose-dependent manner (Fig 3A, top image). To confirm that ATPIF1 binding to CV was decreasing ATP hydrolysis as expected, we subjected the same gel to an ATP hydrolytic in-gel activity assay (Fig 3A, middle image). ATPIF1-GFP inhibited in-gel hydrolysis in a dose-dependent manner, confirming the functionality of ATPIF1-GFP in this procedure. Incubation with ATPIF1-GFP had no effects on the levels or integrity of CV (Fig EV2A). To further validate that inhibition of hydrolysis by recombinant ATPIF1 can be achieved when CV is within mitochondrial membranes, we measured CV hydrolytic activity using HyFS in frozen and thawed mitochondria. Increasing concentrations of ATPIF1 resulted in a dose-dependent inhibition of hydrolysis measured by HyFS as well (Fig 3C and D), without affecting ETC complex activities (Fig EV2B–D).

We next addressed the hypothesis that by acting as a competitor of CV, EPI could prevent ATPIF1 binding to CV using either heart mitochondria or bovine-purified CV tetramers (Jiko *et al*, 2015; Urbani *et al*, 2019). In both preparations, we performed binding competition assays between EPI and ATPIF1-GFP (Figs 3E–G and EV2E and F). In both purified tetramers and isolated mitochondria, EPI prevented the binding of ATPIF1-GFP (F488) to CV (Fig 3E, top panel, Figs 3F and EV2F). While EPI prevented ATPIF1 binding to CV (Fig 3E, top panel, and Fig 3F), EPI mimicked the ability of ATPIF1 to inhibit in-gel hydrolysis activity (Fig 3E, middle panel, and Fig 3G) without changing the levels of CV (Fig EV2E),

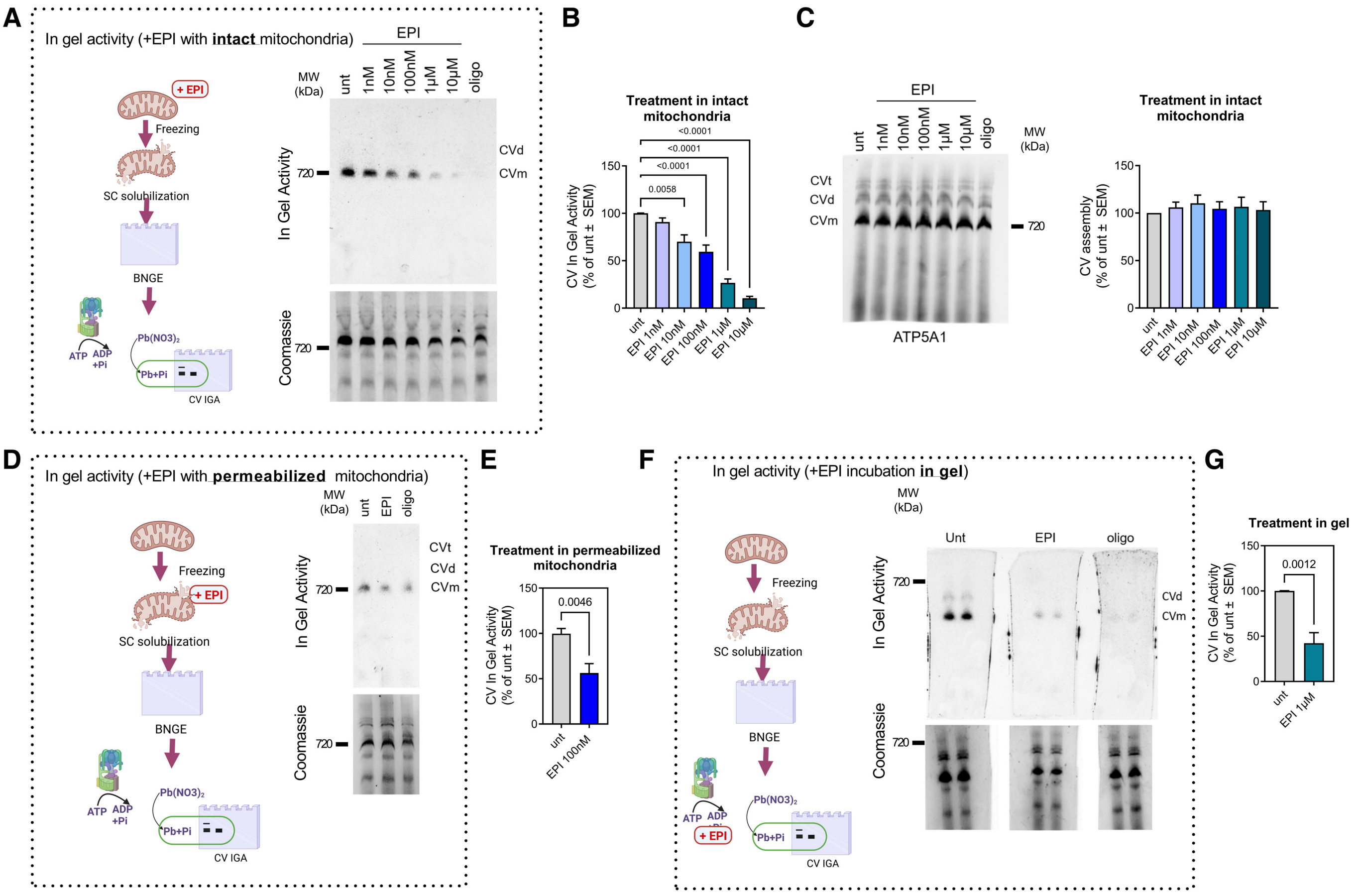

Figure 2. EPI inhibits ATP hydrolysis by direct binding to CV.

A CV in gel ATP hydrolytic activity in intact mitochondria incubated in the presence of the indicated concentrations of EPI. EPI was added to samples of isolated mouse heart mitochondria that were then lysed by freeze/thaw and digitonin. In gel ATP hydrolysis was allowed to proceed for 3 h (top). Coomassie staining is shown as loading control (bottom). Oligomycin (oligo) was used as a control for ATP synthesis and ATP hydrolysis.

B Quantification of in gel ATP hydrolytic activity of panel A under the indicated EPI concentrations ($n \geq 5$).

C Representative blot (left) and quantification (right) showing CV assembly by BNGE in mouse heart mitochondria treated with EPI as in (A) ($n \geq 3$).

D CV in gel ATP hydrolytic activity in permeabilized mouse heart mitochondria treated with EPI. EPI and oligo were added after mitochondria were permeabilized. In gel activity is shown after 3 h incubation (top). Coomassie staining was used as loading control (bottom).

E Quantification of CV in gel hydrolytic activity of panel (D) ($n = 6$).

F CV in gel ATP hydrolytic activity in mouse heart mitochondria where EPI or oligo were added in the assay buffer after mitochondrial complexes and supercomplexes were separated by BNGE. CV in gel ATP hydrolytic activity is shown after 3 h incubation (top). Coomassie staining was used as loading control (bottom).

G CV in gel ATP hydrolytic activity quantification of panel (F) ($n = 5$).

Data information: For each biological replicate, technical replicates were averaged. Data represent average ± SEM. (B) Two-way ANOVA followed by Dunnett's multiple comparisons test and (E, G) unpaired *t*-test show statistical differences depicted by *P*-value.
Source data are available online for this figure.

supporting that EPI functions as an ATPIF1 competitive mimetic for CV binding.

Finally, to investigate whether the capacity of EPI to decrease CV-mediated ATP hydrolysis is dependent on ATPIF1 expression, we studied the in-gel CV hydrolytic activity in diverse preparations of purified bovine CV (Jiko *et al*, 2015; Urbani *et al*, 2019) that contain different levels of ATPIF1, as well as oligomeric, tetrameric and monomeric forms of CV (Fig EV2G). We found that EPI inhibited ATP hydrolysis independently of the amount of ATPIF1 (Fig EV2H–J and Appendix Fig S4). Interestingly, both EPI and oligomycin inhibited ATP hydrolysis, not only in the fully assembled CV and its oligomers, but also in the F_1 subcomplex (Fig EV2H and J and Appendix Fig S4). To further confirm that EPI inhibition of ATP hydrolysis does not require ATPIF1, we analyzed the efficacy of EPI treatment in tissues expressing different amounts of ATPIF1 using HyFS (Fig EV2K). EPI had the same efficacy in decreasing ATP hydrolysis in tissues with low-ATPIF1 expression (Fig 3H). Consequently, and in agreement with the data in purified CV, we conclude that the inhibitory action of EPI on ATP hydrolytic activity does not require the presence of ATPIF1.

(+)-Epicatechin mimics ATPIF1 and blocks hydrolysis in live cells

To determine whether ATPIF1 binds to CV in intact cells, we utilized the Proximity Ligation Assay (PLA) to quantify the binding of the endogenous ATPIF1 with the alpha subunit of CV F_1 head (Fig

 © 2023 The Authors

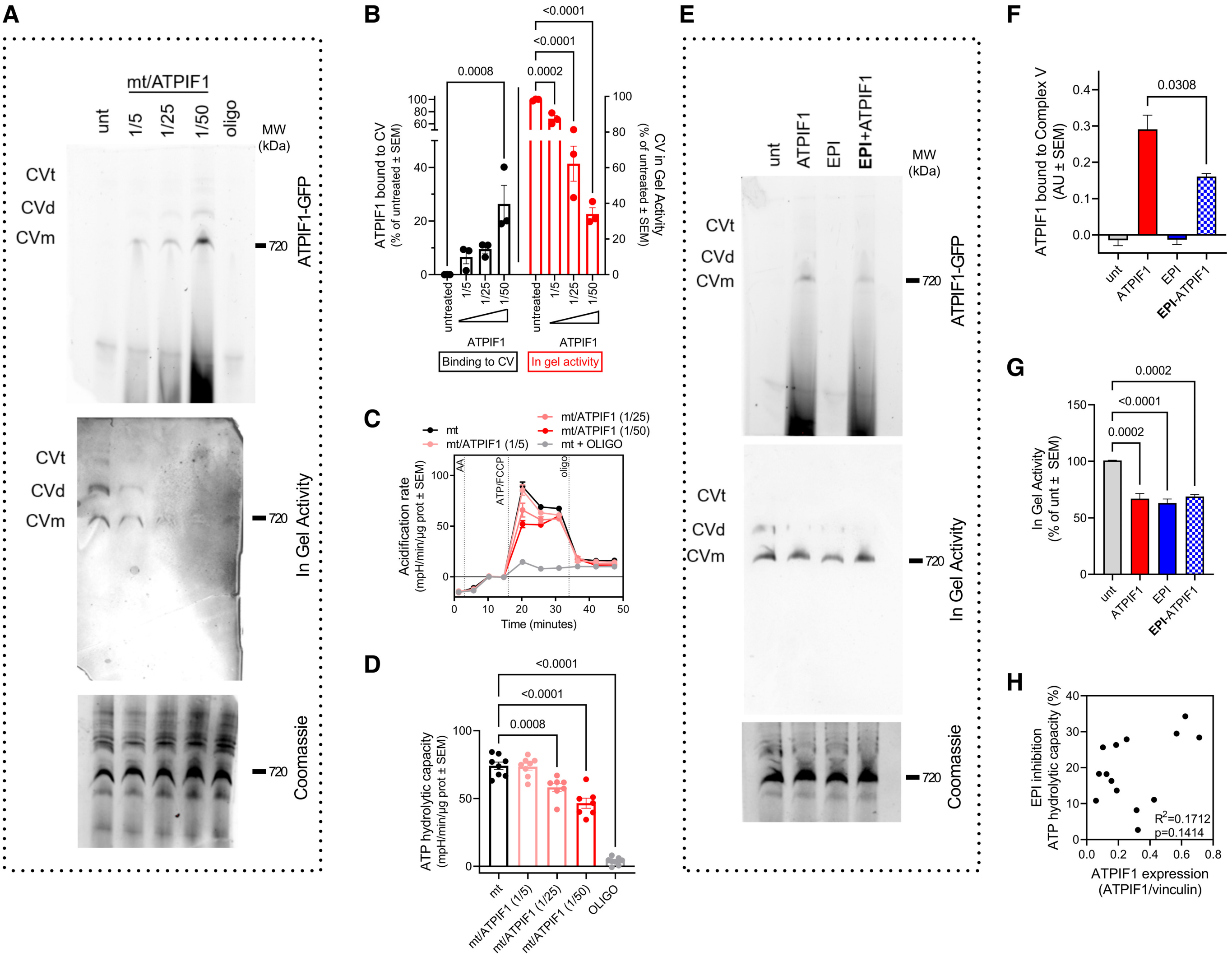

Figure 3. EPI competes with ATPIF1 on binding to CV (see also Fig EV2).

A–G Binding of purified recombinant ATPIF1-GFP to CV. Mitochondrial membranes were disrupted by freeze–thaw and exposed to ATPIF1-GFP at different ratios of mitochondrial (mt) protein/ATPIF1 purified protein. Binding was assessed in BNGE by quantifying ATPIF1-GFP fluorescence. (A) BNGE assessed for ATPIF1-GFP binding (top); CV hydrolytic activity (middle) and protein loading (bottom). (B) Quantification of in gel CV–ATP hydrolytic activity shown in panel (A). On the left-hand side are the levels of CV–bound ATPIF1 and on the right-hand side is CV–ATP hydrolytic activity normalized to the levels of fully assembled CV ($n = 3$). (C, D) Representative acidification rate profile showing the effects on CV–ATP hydrolytic activity (C) and its quantification (D) ($n = 4$). (E–G) Binding competition assay between ATPIF1-GFP and EPI to CV. (E) BNGE assessed for ATPIF1-GFP binding (top); CV hydrolytic activity (middle) and protein loading (bottom). (F, G) Quantification of gels shown in panel (E). (F) Quantification of ATPIF1 bound to monomeric CV under the indicated treatments ($n = 3$). (G) Quantification of in gel ATP hydrolytic activity under the indicated treatments ($n = 3$). Note that EPI reduces ATPIF1 binding while maintaining the same inhibitory effect on ATP hydrolysis.

H Plot illustrating the lack of correlation of ATPIF1 expression levels with EPI effect in inhibiting maximal CV–ATP hydrolytic activity. Each point represents a biological sample replica.

Data information: For each biological replicate, technical replicates were averaged. Data represent average ± SEM. (B, D, G) Two-way ANOVA followed by Šídák's multiple comparisons test shows statistical differences depicted by *P*-value. (F) Unpaired *t*-test show statistical differences depicted by *P*-value. (H) Simple linear regression including R^2 and *P*-value.
Source data are available online for this figure.

4A). PLA detects the events of proximity between two antibodies. The two antibodies carry complementary PCR primers that allow for a PCR reaction to occur when the two antibodies bind two targets that are located within 10 nm or less of each other. The PCR reaction produces a fluorescent signal, and the localization and frequency of proximity events can be quantified. To develop the PLA assay between CV and ATPIF1, we used an anti-ATPIF1 antibody and an anti-ATP5A1 antibody. PLA analysis in control human fibroblasts treated with EPI for 24 h shows that EPI treatment decreases ATPIF1 binding to CV in control cells (Fig 4B) for all the concentrations tested, further supporting that in intact living cells EPI can either prevent or displace ATPIF1 binding to CV. BNGE analysis of

© 2023 The Authors

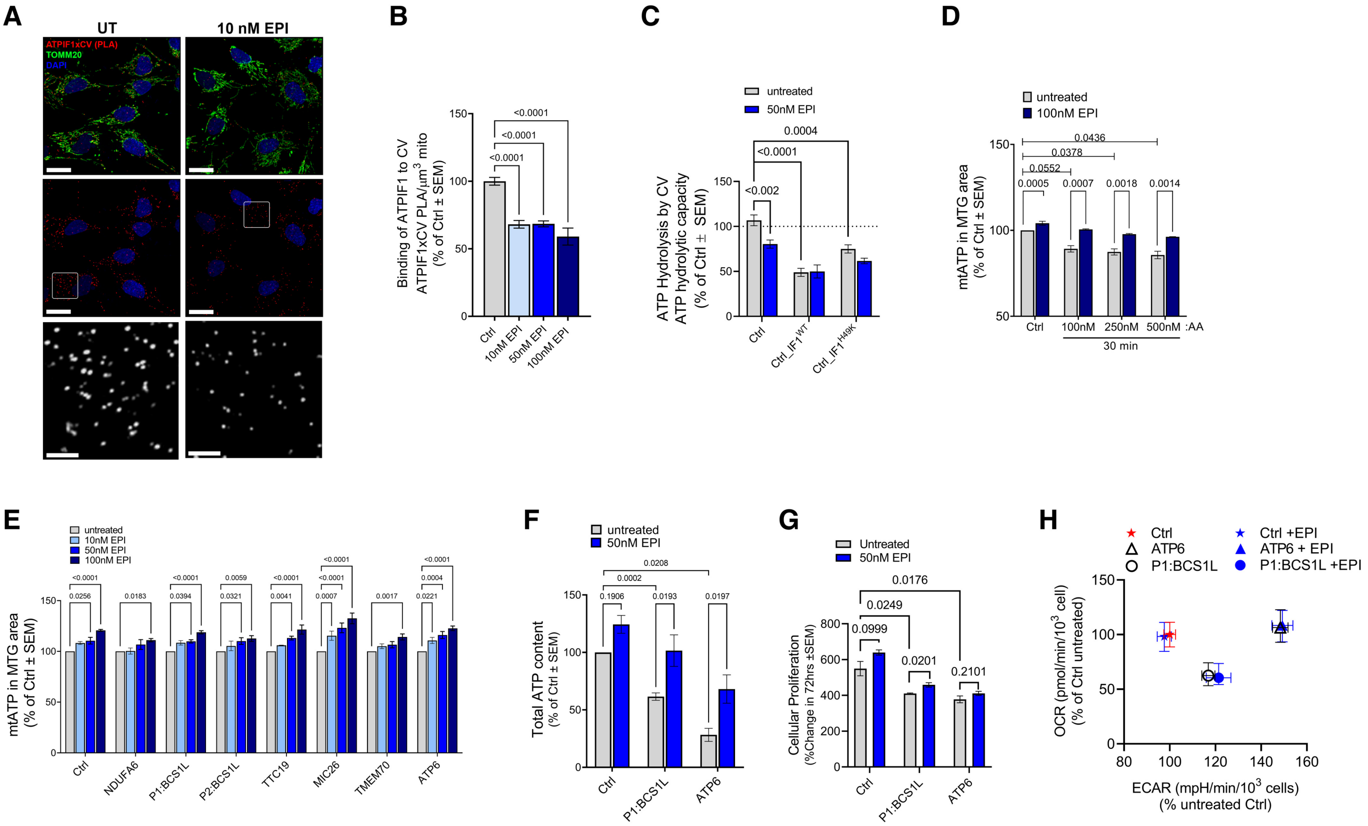

Figure 4. In mitochondrial disease models, EPI inhibits ATP hydrolysis, increasing cellular ATP content without affecting respiration (see also Fig EV3).

A, B PLA of CV and ATPIF1 produces a red fluorescence signal where anti-ATPIF1 and anti-ATP5A1 antibodies are localized at a distance < 10 nm of each other. Anti-TOMM20 (green) was used to reveal mitochondrial architecture. (A) Representative high-resolution Airyscan confocal images showing control fibroblasts treated with EPI for 24 h. Maximum intensity projection is shown. Scale bars: 20 and 5 μm in zoomed image. Note that PLA reveals the spatial distribution of ATPIF1 binding along the mitochondrial length. (B) Analysis of ATPIF1-CV complexes occurrence quantified as PLA dots per μm^3 of mitochondria. Effect of EPI is shown relative to untreated (n = 2 from > 50 replicates per n).

C Effect of EPI on CV ATP hydrolytic capacity in cells overexpressing either $ATPIF1^{WT}$ or its continuously active form, $ATPIF1^{H49K}$. Hydrolytic capacity is measured by the acidification rates and is normalized to maximal oxygen consumption on succinate + rotenone (Max OCR on SR). Effect of EPI is shown as % of control cells, untreated with EPI. Cells were treated for 24 h with 50 nM EPI (n = 4). Note that in cells overexpressing ATPIF1, EPI treatment does not result in further inhibition of ATP hydrolysis.

D Effect of EPI on the mitochondrial matrix ATP pool (mtATP) under conditions where mitochondrial respiration is inhibited by Antimycin A (AA). mtATP content measured as fluorescence intensity per mitochondria area and shown as % of control untreated (n = 3). Control fibroblasts were incubated for 30 min with DMSO or EPI in different concentrations of AA. Mitochondrial area was quantified using MTG staining.

E mtATP content measured in human fibroblasts from control and mitochondrial disease patients, after 30 min treatments with increasing concentrations of EPI as indicated. Analysis shows the mean intensity of mtATP probe in MTG area normalized per % of correspondent untreated cells (n = 3).

F–H Effects of EPI treatment on fibroblasts with a CIII assembly defect (P1:BCS1L) and fibroblast with a CV ATP synthesis defect (ATP6). (F) Total cellular ATP content shown as % of control untreated cells. Cells were treated for 24 h (n = 3). (G) Cell proliferation rates shown as % change from 0 h (untreated) to 72 h after treatment (n = 3). (H) Basal OCR vs ECAR in Control, CIII- and CV-deficient patient fibroblasts treated with and without 50 nM EPI (n = 3).

Data information: (A–H) In all cases, for each biological replicate, technical replicates were averaged. Data represent average ± SEM. Two-way ANOVA followed by Šídák's multiple comparisons test shows statistical differences depicted by P-value. (G) Unpaired t-test versus untreated conditions shows statistical differences depicted by P-value.
Source data are available online for this figure.

control cells cultured for 24 h demonstrated that EPI treatment results in a decrease in the levels of ATPIF1 bound to assembled CVI, although the observed trend did not reach statistical significance (Fig EV4C and D).

To distinguish if EPI prevents ATPIF1 binding to CV or if EPI can displace the already bound ATPIF1 from CV, we ran displacement and competition assays side by side. To test for displacement, we incubated purified CV with ATPIF1-GFP for 5 min to allow for binding to take place. EPI was then added to the mix for an additional incubation of 10 min before the sample was analyzed by BNGE, to determine the amount of ATPIF1-GFP bound to CV (Fig EV2F). In a parallel competition assay, we incubated purified CV tetramers with ATPIF1-GFP in the presence of EPI. We found that EPI treatment did not displace ATPIF1-GFP that was already bound to CV. However, if EPI and ATPIF1-GFP were incubated concurrently, ATPIF1 binding to CV was decreased. These results suggest that EPI can prevent ATPIF1 binding to CV, but cannot displace an already bound ATPIF1 from CV.

In a living cell, EPI can both prevent ATPIF1 binding and inhibit ATP hydrolysis, however the net effect on inhibition of ATP

 © 2023 The Authors

hydrolysis under conditions in which both mechanisms are in competition has yet to be explored. To address this question, we overexpressed ATPIF1 (Fig EV3A and B) in the presence or absence of EPI and measured maximal ATP hydrolytic capacity (Fig 4C). We also tested EPI competition with the most active form of ATPIF1, a constitutively active mutant of ATPIF1, H49K, whose binding is insensitive to changes in pH (Schnizer *et al*, 1996; Garcia-Aguilar & Cuezva, 2018).

Both ATPIF1-WT and ATPIF1-H49K overexpression decreased maximal ATP hydrolytic capacity in control fibroblasts (Fig 4C). Addition of EPI to cells overexpressing either ATPIF1-WT or ATPIF1-H49K, did not result in any further inhibition beyond the effect induced by the overexpression of the two forms of ATPIF1. These data suggest that by competing with ATPIF1 on CV binding, EPI does not produce the same inhibition of hydrolysis that can be produced by ATPIF1. These results suggest that EPI is more likely to be efficacious in conditions where ATPIF1 levels are reduced.

Under complete inhibition of respiration, selective hydrolysis inhibition of CV preserves mtATP, without inducing depolarization

Previous studies have demonstrated that ATP hydrolysis by CV is required to preserve mitochondrial membrane potential under conditions of impaired respiratory function when PMF is lost (Connolly *et al*, 2018). To test the effect of EPI on mitochondrial membrane potential, we adapted the previously well-described Antimycin A (AA) model to induce CV–ATP hydrolysis. This model takes advantage of the opposite effect of oligomycin in ATP-producing versus ATP-hydrolyzing mitochondria. While oligomycin treatment in cells with intact oxidative phosphorylation (OXPHOS) results in mitochondrial membrane potential hyperpolarization, the addition of oligomycin to cells pretreated with AA, to block respiration, results in depolarization. This shift in oligomycin response, from hyperpolarization to depolarization termed "oligomycin null point" represents the state at which membrane potential relies on CV–ATP hydrolysis (Rego *et al*, 2001; Vesce *et al*, 2005; Nicholls *et al*, 2013).

To determine if under complete inhibition of respiration EPI can induce membrane potential depolarization, we first reproduced the oligomycin null-point phenomenon in human fibroblasts using AA and oligomycin. To ensure that effective concentrations of AA and oligomycin are used, we ran a parallel analysis of OCR with each of the compounds separately. OCR analysis determined that AA at 50 nM results in complete inhibition of respiration (Fig EV3C, left panel) with a concomitant increase in extracellular acidification rate (ECAR) indicating a glycolytic shift in ATP production (Fig EV3C, right panel), while oligomycin showed a dose-dependent effect on respiration between 50 nM and 5 μM (Fig EV3D).

To produce an oligomycin null-point phenomenon, we treated fibroblasts with increasing concentrations of AA for 30 min in the presence or absence of oligomycin. We monitored membrane potential using the fluorescence membrane potential indicator, tetramethylrhodamine ethyl ester (TMRE), and we used Mitotracker Green (MTG) (membrane potential independent) to control for mitochondrial mass. As expected, while oligomycin treatment alone led to hyperpolarization, the combination of AA and oligomycin at 5 μM resulted in a statistically significant depolarization (Fig EV3E and F). Lower concentrations of oligomycin trended toward a dose-dependent depolarization (Fig EV3F), although did not reach statistical significance. This result indicates that complete inhibition of CV is required to induce the oligomycin null-point. Moreover, this assay established that treatment of these cells with 5 μM AA for 30 min is required to produce the oligomycin null-point.

To determine if, as oligomycin, EPI can induce depolarization under complete inhibition of respiration, we repeated the same experiment, but this time replaced oligomycin with EPI. Remarkably, EPI did not lead to membrane potential depolarization (Fig EV3E). Altogether, we conclude that in order to induce membrane potential depolarization under AA, a complete inhibition of ATP hydrolysis is required, and that partial inhibition by low-oligomycin concentrations or by EPI is not sufficient to overcome the compensatory mechanisms that maintain membrane potential.

To determine the effect of inhibition of CV–ATP hydrolysis on mitochondrial matrix ATP content (mtATP), we monitored mtATP levels by using BioTracker™ (Wang *et al*, 2016) staining detected by fluorescence microscopy. Treating fibroblasts with AA for 30 min induced a dose-dependent depletion of the mitochondrial matrix ATP pool (Fig 4D). Remarkably, EPI treatment restored mtATP in AA-treated cells (Fig 4D). This result is in agreement with recent studies by Rieger *et al* (2021), showing that mtATP content specifically responds to changes in ATPIF1 expression.

Inhibition of CV–ATP hydrolysis by EPI treatment preserves mtATP and total cellular ATP content, and restores the proliferation rate in fibroblasts from patients with mitochondrial diseases

We next aimed to determine whether EPI treatment effectively decreases ATP hydrolysis in fibroblasts from patients with mitochondrial diseases, caused by mutations in mitochondrial proteins. EPI treatment for 30 min induced a dose-dependent increase in mtATP in each of the mutant fibroblasts tested (Fig 4E). To rule out that EPI was increasing mitochondrial respiration to restore mtATP levels, we monitored OCR before and after the addition of EPI. No differences in OCR were observed in intact cells after EPI injection (Fig EV3G). Such a rapid increase in mtATP is consistent with EPI directly inhibiting CV hydrolytic activity in isolated mitochondria and purified CV (Fig 2).

To determine whether the EPI-mediated increase in mtATP content initiated a restoration in cellular ATP homeostasis, we measured the effects of a 24 h EPI treatment on total cellular ATP content (Figs 4F and EV3H). Remarkably, treatment with EPI resulted in a significant increase in ATP in two of the mutant fibroblasts tested, a CIII deficiency (CIII-BCS1L) and a CV deficiency (CV-ATP6) (Fig 4F). Other mutants (P2:BCS1L, MIC26, and TMEM) only showed a slight increase in total cellular ATP, as did the control fibroblasts (Fig EV3H). Furthermore, CIII-deficient cells treated with EPI showed a statistically significant, mild improvement in proliferation rates after 72 h of treatment, while control and CV-deficient cells showed a similar trend, although not statistically significant (Fig 4G).

Altogether, these results demonstrate that impaired OXPHOS and CV–ATP hydrolysis both contribute to the depletion in cellular ATP observed in mutant fibroblasts.

© 2023 The Authors

Inhibition of ATP hydrolysis restores ATP content without increasing respiratory function or changing complex assembly and content

The above results suggest that the inhibition of hydrolysis alone is sufficient to improve ATP availability in conditions of impaired respiration. However, since the observed increase in ATP content could be the result of improved respiration, we further examined the effect of EPI on respiratory chain structure and function.

We chose to examine the effect of EPI on respiration in two mutant cells: CIII-BCSL1 and CV-ATP6. Both have reduced ATP content, but through different mechanisms: in BCS1L mutant, ATP depletion is caused by the combination of impaired respiration and increased ATP hydrolysis, whereas in the CV-ATP6 mutant, reduction in ATP content is primarily caused by impaired ATP synthesis by CV. We examined basal respiration, a parameter that directly responds to increased OXPHOS. In this assay, we did not treat with oligomycin or FCCP to avoid perturbation of ATP hydrolysis.

When compared with control cells, BCS1L mutant cells showed a decrease in basal, maximal, and ATP-linked respiration while the ATP6 mutants showed only an increase in maximal respiration (Fig 4H and Appendix Fig S5), as previously reported (Fernandez-Vizarra *et al*, 2007; D'Aurelio *et al*, 2010). Treatment with EPI did not result in any changes to OCR. ECAR was increased in both mutants when compared to control cells, in agreement with the glycolytic shift observed in mitochondrial diseases. EPI treatment showed a tendency to decrease lactate levels only in CIII-deficient cells, supporting a decrease in ATP demand induced by treatment with EPI (Fig EV3I). The absence of such a trend in CV-deficient cells supports that ATP synthesis is the major contributor to decreased ATP availability induced by CV-ATP6 mutation.

EPI did not affect the structure or content of CIII or CV in these cells as determined by BNGE analysis, further confirming that the increase in ATP content is not mediated by changes in complex assembly or content (Fig EV4A–D).

(+)-Epicatechin binds to mitochondrial ATP synthase and replaces ATPIF1 in intact living cells

We next assessed the amount of ATPIF1 bound to ATP synthase in CIII-BCS1L and CV-ATP6 mutant and control fibroblasts by PLA (Fig 5A–C), which revealed that ATPIF1 binding to ATP synthase was reduced in both mutants (Fig 5B). EPI treatment further decreased ATPIF1 binding both in control and in CV-ATP6 mutant cells, while the already low levels of ATPIF1 bound in CIII-BCS1L cells remained unchanged (Fig 5C). Measurements of ATP hydrolysis revealed a negative correlation between ATPIF1 binding to CV and the levels of ATP hydrolysis in these cells (Fig 5D). This negative correlation between ATPIF1 binding and ATP hydrolysis was absent in cells treated with EPI, where ATP hydrolysis was markedly decreased independent of the levels of ATPIF1 binding to CV.

Similar to fibroblasts from patients with other mitochondrial diseases (Fig 1E), we found that CIII-BCS1L fibroblasts express low levels of ATPIF1, suggesting the possibility that the lack of endogenous hydrolysis inhibition contributes to EPI efficacy in these cells. We, therefore, compared the effect of restoring ATPIF1 expression and activity with the effect of EPI treatment. We produced BCS1L cells stably overexpressing either WT ATPIF1 or the continuously active mutant of ATPIF1, H49K (Fig EV3A and B). Both ATPIF1-WT and ATPIF1-H49K expression decreased CV–ATP hydrolytic capacity in CIII-BCS1L cells, mimicking the effects of EPI in these same mutants (Fig 5E). In marked contrast to EPI treatment, the inhibition of hydrolysis by ATPIF1 overexpression failed to improve either mtATP or total cellular ATP content (Fig 5F and G). These data are consistent with the hypothesis that ATPIF1 blocks the rotation of ATP synthase in both forward and reverse directions, and explains why ATPIF1, in contrast to EPI, does not increase ATP content in CIII-BCS1L cells.

To determine the role of ATPIF1 content and activity in the efficacy of EPI, we analyzed the efficacy of EPI after restoring ATPIF1 levels by overexpressing WT ATPIF1, or ATPIF1 H49K (Figs 4E, 5D and EV3A and B) (Schnizer *et al*, 1996; Garcia-Aguilar & Cuezva, 2018). Addition of EPI in combination with the overexpression of ATPIF1 or ATPIF1 H49K did not result in any additional inhibition of ATP hydrolytic capacity beyond the level achieved with either one of the constructs. Furthermore, addition of EPI in cells already overexpressing WT or H49K ATPIF1 did not improve mtATP or total cellular ATP content.

To further elucidate the mechanism by which ATPIF1 overexpression prevents the effects of EPI, we examined if EPI could compete against ATPIF1 for binding to CV, PLA analysis showed increased ATPIF1 binding to CV in ATPIF1 WT and H49K overexpressing cells, validating that the constructs encoding for ATPIF1 WT and H49K produced proteins capable of incorporating into CV (Fig EV3B). EPI was not able to decrease the levels of ATPIF1 binding to CV in CIII-BCS1L cells overexpressing ATPIF1-WT or ATPIF1-H49K (Fig EV4E and F). These data are in agreement with EPI effectively inhibiting CV hydrolytic activity in conditions where CV binding sites to ATPIF1 are available.

Inhibition of ATP hydrolysis by EPI preserves muscle function in mice with muscular dystrophy

To determine whether inhibiting CV-mediated ATP hydrolysis can prevent tissue dysfunction *in vivo*, we chose Duchenne Muscular Dystrophy (DMD) as a disease model, in which mitochondrial depolarization has been reported in association with ATP depletion (Bulfield *et al*, 1984; Sicinski *et al*, 1989; McGreevy *et al*, 2015). DMD is caused by a mutation in the cytoskeletal protein dystrophin. The *mdx* genetic mouse model of dystrophin deficiency has been used extensively to study the pathophysiology of DMD (McGreevy *et al*, 2015; Yucel *et al*, 2018; Hammers *et al*, 2020). Loss of dystrophin destabilizes the myofiber membrane to cause pathological calcium overload in the cytosol during physical activity (Bonilla *et al*, 1988; Mareedu *et al*, 2021). Cytosolic calcium overload leads to an excessive uptake of calcium by the mitochondria resulting in mitochondrial depolarization and permeability transition (Moore *et al*, 2020; Budzinska *et al*, 2021; Dubinin *et al*, 2021). While ATP depletion has been documented in *mdx* mice and in DMD patients (Kelly-Worden & Thomas, 2014; Budzinska *et al*, 2021), the contribution of ATP hydrolysis by CV has not been tested.

To study the contribution of CV-mediated ATP hydrolysis to dystrophic pathology, we used D2.*mdx* mice, harboring the *mdx* mutation in a DBA/2J genetic background (D2.*mdx*). At 12 weeks of age, D2.*mdx* mice were subjected to exercise-induced gastrocnemius eccentric injury by stimulating muscle contraction using external

 © 2023 The Authors

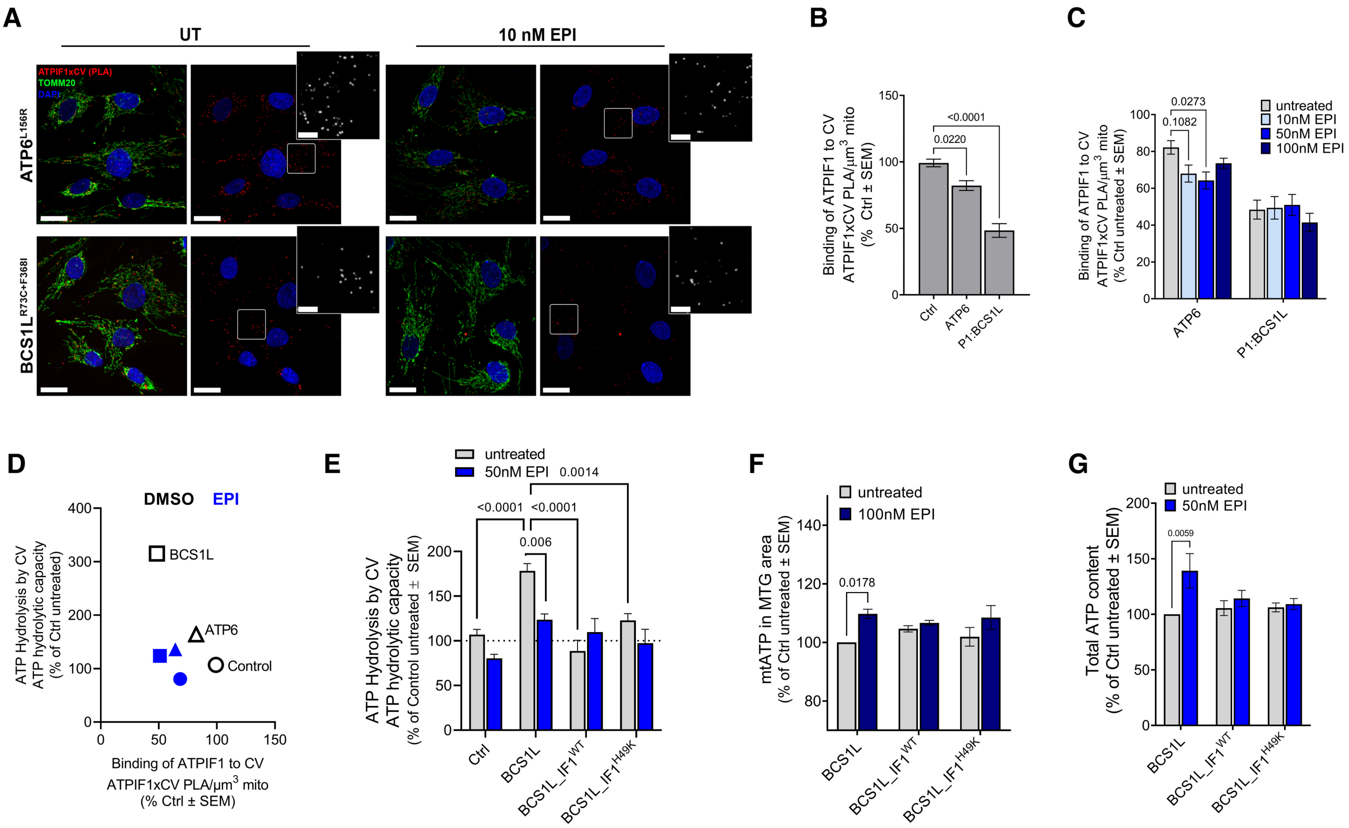

Figure 5. EPI replaces and mimics ATPIF1 inhibition of ATP hydrolysis, and is most effective where ATPIF1 levels are low (see also Fig EV4).

A–D PLA of CV and ATPIF1 (red). Anti-TOMM20 (green) was used to reveal mitochondrial architecture. (A) Representative confocal micrographs showing fibroblasts treated with EPI for 24 h. Maximum intensity projection is shown. Scale bars: 20 and 5 μm zoomed images. (B) Analysis of ATPIF1-CV complexes occurrence quantified as PLA dots per μm^{3}of mitochondria in patient fibroblasts normalized in % of Ctrl untreated (n = 2 from > 50 replicates per n). (C) Effect of EPI on ATPIF1-CV complexes occurrence quantified as PLA dots per μm^3 under the indicated conditions. Values are shown as relative to control untreated. (D) Correlation between maximal ATP hydrolytic capacity and CV-bound ATPIF1. Correlation is shown for control and mutant fibroblasts treated with DMSO or EPI. Note that ATP hydrolysis is increased where the levels of CV-bound ATPIF1 is decreased (BCS1L), and that in presence of EPI the biggest decrease in hydrolysis is observed in low ATPIF1 binding condition.

E–G Effect of EPI on CIII-deficient (BCS1L) cells stable-expressing ATPIF1^{WT} or ATPIF1^{H49K}. (E) CV ATP hydrolytic capacity measured by the acidification rate and normalized maximal respiration. Maximal respiration was determined using the OCR channel (Max OCR on SR). Effect of EPI is shown as % of control cells, untreated with EPI. Cells were treated for 24 h with 50 nM EPI ($n \geq 4$). (F) mtATP content measured as fluorescence intensity per mitochondria area and shown as % of control untreated (n = 5). Mitochondrial area was quantified using MTG staining. Cells were treated for 30 min. (G) Total cellular ATP content shown as % of untreated cells. Cells were treated for 24 h ($n \geq 4$).

Data information: In all cases, data represent average ± SEM. For each biological replicate, technical replicates were averaged. Two-way ANOVA followed by Šídák's multiple comparisons test shows statistical differences depicted by P-value.
Source data are available online for this figure.

electrodes (Blaauw *et al*, 2010; Khairallah *et al*, 2012; Call & Lowe, 2016; Hammers *et al*, 2020). We analyzed the extent of CV–ATP hydrolysis in gastrocnemius from WT and D2.*mdx* mice at rest or after exercise-induced injury. In each mouse, the right leg was subjected to exercise-induced injury and the left leg was kept at rest as a control. Gastrocnemius muscle was collected 1 or 24 h post-injury (pi). We found that the gastrocnemius from *mdx* mice had decreased CV (ATP5A1) and CII (SDHA) content (Figs 6A and EV5A). Exercise-induced injury further decreased CV (ATP5A1) and CII (SDHA) content 24 h after injury in *mdx* mice, while no decrease was observed in WT mice (Fig 6A). These decreases in CV and CII occurred in the absence of changes in mitochondrial content (Appendix Fig S6). HyFS analyses of gastrocnemius homogenates from *mdx* mice revealed an increase in CV–ATP hydrolytic capacity at both 1 and 24 h postinjury (Figs 6B and EV5B, respectively). In contrast, WT mice did not show any change in ATP hydrolytic capacity following the injury. Moreover, gastrocnemius of *mdx* mice revealed that exercise-induced injury results in cleavage of the mitochondrial inner membrane protein OPA1 (Figs 6C and EV5C), as well as cytochrome c release (Figs 6D and EV5D). Together, these findings demonstrated that exercise-induced injury in *mdx* mice resulted in mitochondrial changes characteristic of calcium overload.

The observation of a robust increase in CV–ATP hydrolysis in injured *mdx* mice revealed that these mice are a suitable disease model to test the impact of inhibiting CV–ATP hydrolysis *in vivo* on pathology. EPI administered twice a day for 2 weeks decreased susceptibility to injury in *mdx* mice in a dose-dependent manner, when compared with vehicle treated mice (Fig 7A and B). Remarkably,

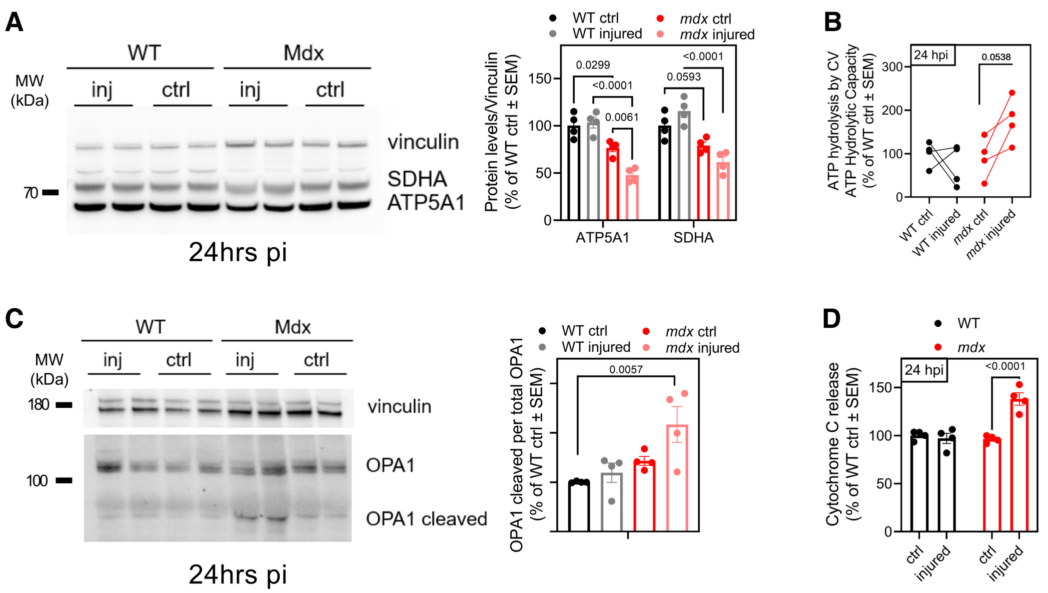

Figure 6. CV–ATP hydrolysis is increased in *mdx* mice, a model of Duchenne Muscular Dystrophy (see also Fig EV5).

A–D D2.*mdx* mice, harboring the *mdx* mutation in a DBA/2 J genetic background (D2.*mdx*) were subjected to exercise-induced gastrocnemius injury by stimulating muscle contraction (eccentric injury). Levels of CV, ATPIF1 and maximal CV ATP hydrolytic capacity were measured. (A) Representative western blot image (left) and quantification (right) showing CV (ATP5A1) and CII (SDHA) levels in gastrocnemius homogenate in WT and *mdx* mice after 24 h of eccentric injury (inj). Vinculin was used as loading control. Note that only in *mdx* mice eccentric injury results in reduced CV content. (B) Maximal CV ATP hydrolytic capacity per CV content measured in gastrocnemius homogenates from WT and *mdx* mice 24 h after eccentric injury. Note that hydrolytic capacity per CV was increased in mdx mice subjected to injury. (C) Analysis of levels of the cleaved OPA1 in gastrocnemius homogenate in WT and *mdx* mice 24 h after eccentric injury. (D) Cytochrome c release in gastrocnemius supernatants of WT and *mdx* at 24 h post-injury measured by ELISA. Note that induction of OPA1 cleavage and Cyt C release in response to injury occur only in the *mdx* mice. From (A–D), $n = 4$. For each biological replicate, technical replicates were averaged. Data represent average ± SEM. Two-way ANOVA followed by Šídák's multiple comparisons test shows statistical differences depicted by *P*-value.

Source data are available online for this figure.

EPI treatment decreased ATP hydrolysis in the injured muscle from *mdx* mice, an effect that was accompanied by a trend towards increased CV content (Figs 7C and EV6A). Analysis of muscle function demonstrated that the efficacy of EPI treatment improving the post-injury muscle force was strongly correlated with ATP hydrolysis inhibition (Fig 7D). Additionally, EPI treatment blocked OPA1 cleavage (Fig 7E) and cytochrome c release (Figs 7F and EV6B). A dynamic process of plasma membrane damage and repair occurs when mdx mice develop exercise induced-muscle injury, during which the material from the interstitial fluid can enter the cell. The extent and duration of plasma membrane barrier breach are tested using Evans Blue Dye (EBD), by measuring how much dye can enter the cell through regions of broken plasma membrane (Hamer *et al*, 2002). Twenty-four hours prior to injury, *mdx* mice were injected with EBD and the number of Evans Blue positive cells was quantified post-injury. EPI treatment decreased the number of Evans Blue positive fibers, indicating that EPI protected the integrity of gastrocnemius muscle fibers (Fig 7G). To further validate that the mechanism of improvement was through inhibition of hydrolysis, we studied human myotubes that model DMD *in vitro*. Our data showed that EPI was able to inhibit hydrolysis and preserved membrane integrity in DMD mutant cells (Fig EV6C–E), supporting that the beneficial effect of EPI can be cell autonomous. Principal component analysis (PCA) of different variables measuring overall muscle status, including muscle function, bioenergetics, OPA1 cleavage, cytochrome c release, and cell membrane integrity, showed that EPI triggered the improvement in muscular function in *mdx* mice, and restored parameters of muscle function in WT-injured mice, that clustered with the control (non-injured) groups (Fig 7H). These data support the hypothesis that ATP hydrolysis by CV contributes to muscle damage and accelerates mitochondrial apoptosis, via OPA1 cleavage.

Discussion

The reversal of Complex V activity was previously shown to be a protective response activated by acute stressors, such as heart ischemic injury and hypoxia/anoxia (Classen *et al*, 1989; St-Pierre *et al*, 2000). The proton extrusion mediated by CV hydrolyzing ATP, mitigates mitochondrial depolarization and apoptosis induced by these stressors. However, little is known about the local distribution of synthase and hydrolase activity of CV under physiological conditions, or how long-term ATP hydrolysis contributes to pathological conditions.

In isolated mitochondria, synthetic and reverse hydrolytic activities of ATP synthase were measured in the same preparation and showed that under different states of respiration and membrane potential, heterogeneous function of ATP synthase can co-exist in coupled mitochondria. Accumulating evidence supports the possibility that ATP hydrolysis can occur heterogeneously in the cell and even within individual mitochondrion simultaneously with ATP

 © 2023 The Authors

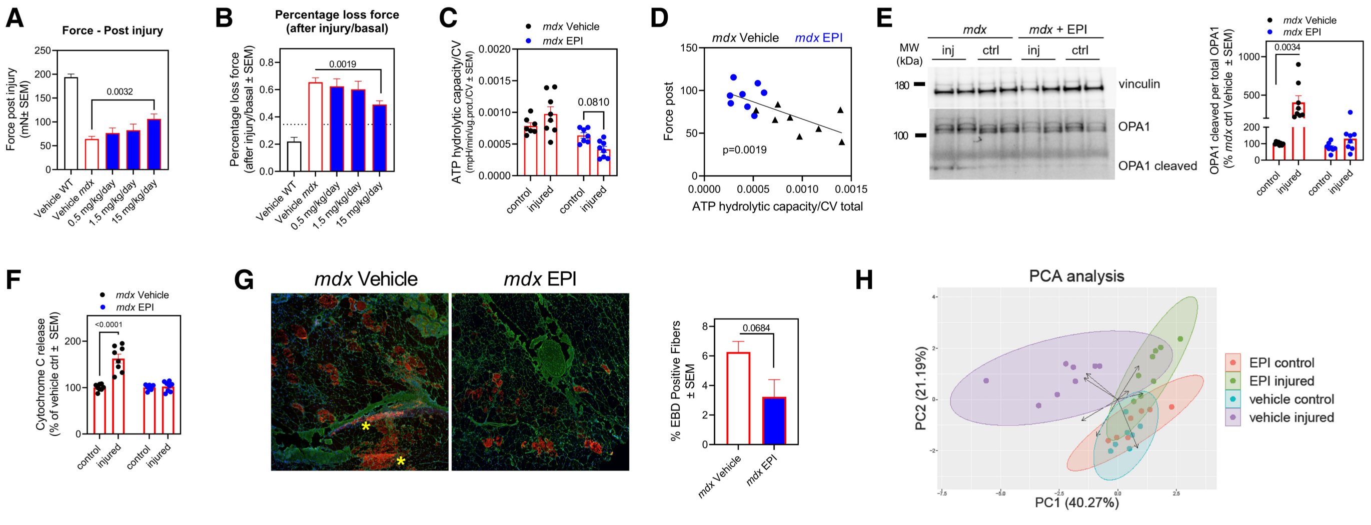

Figure 7. Inhibition of ATP hydrolysis by EPI treatment partially restores muscle function in a mouse model of Duchenne's muscular dystrophy (DMD) (see also Fig EV6).

A–H D2.*mdx* mice were treated with EPI orally twice daily, with doses ranging from 0.5 to 15 mg/kg/day. After 13 days of treatment, mice were subjected to exercise-induced gastrocnemius injury by stimulating muscle contraction and the effect of EPI on muscle force, CV ATP hydrolytic capacity, OPA1 cleavage and Cyt C release were measured. (A) Plantarflexor force quantification after eccentric injury. (B) Percentage of injury inflicted loss of force. (C) Maximal CV ATP hydrolytic capacity per CV content in gastrocnemius homogenates from *mdx* mice treated with vehicle or EPI measured 24 h postinjury. (D) Correlation between muscle force and maximal CV ATP hydrolytic capacity per CV content. Each experimental group is represented with different color. Note that increased CV ATP hydrolytic capacity correlates with reduced muscle force. (E) Levels of cleaved OPA1 in gastrocnemius homogenate from *mdx* mice subjected to the indicated combinations of injury and EPI treatment. (F) Cytochrome c release measured in supernatants from gastrocnemius muscle homogenates of *mdx* mice treated with vehicle or EPI 24 h post eccentric injury. (G) Detection and quantification of myofiber membrane integrity or damage following injury using Evans Blue dye (EBD) in gastrocnemius muscle from vehicle and EPI treated *mdx* mice. Damaged fiber membranes allow dye entry that appears as red color. Yellow asterisks indicate damage fibers ($n \geq 4$). (H) Principal Component Analysis (PCA) of the effect of EPI treatment on the recovery from injury in gastrocnemius muscle of mdx mice. Note that EPI-injured treated group cluster closer to the EPI or vehicle control group than to the vehicle-injured group. From (A–F), $n = 8$.

Data information: For each biological replicate, technical replicates were averaged. Data represent average ± SEM. Two-way ANOVA followed by Šídák's multiple comparisons test (C, E, F) and unpaired *t*-tests (A, B, G) shows statistical differences depicted by *P*-value. (D) Simple linear regression including *P*-value.
Source data are available online for this figure.

synthesis (Wolf *et al*, 2019; Salewskij *et al*, 2020; Rieger *et al*, 2021). Moreover, the inhibition of ATP synthase function was shown to impact the spatiotemporal organization of ATP synthase (Weissert *et al*, 2021), which can regulate the PMF locally (Rieger *et al*, 2021).

Global adaptation to maintain mitochondrial membrane potential was observed in OXPHOS deficiency represented by several ETC mutants in yeast, with transcriptional downregulation of ATP synthase inhibitor Inh1 (Liu *et al*, 2021). We found that this response in yeast is conserved in humans, as fibroblasts from patients with OXPHOS deficiencies showed a decrease in ATPIF1 protein content, revealing the role of ATPIF1 downregulation as a regulatory process during stress adaptation to increase hydrolysis. A paradigm in ATPIF1 function is whether the protein works as a unidirectional inhibitor of the F_1F_0-ATPase, only inhibiting the hydrolase activity of the enzyme, or whether ATPIF1 also inhibits synthesis (Husain & Harris, 1983). This is still under debate, and the discovery of a selective inhibitor of ATP hydrolysis that regulates the ATP synthase similarly to ATPIF1 can help to clarify that open question. In this way, overexpression and silencing of ATPIF1 can be found as beneficial depending on the model analyzed. Overexpression of ATPIF1 was shown to be protective against heart ischemic injury and neurotoxicity by preventing apoptosis (Rouslin *et al*, 1990; Formentini *et al*, 2014). Downregulation of ATPIF1 in cells followed by antimycin treatment was shown to recover cellular viability, while visual impairment was observed in a zebrafish ATPIF1 knockout model (Chen *et al*, 2014; Martin-Jimenez *et al*, 2018). Therefore, we propose that (1) acute adaptations decreasing ATPIF1 levels and reversing ATP synthase to avoid cell death, or (2) inhibiting the excessive hydrolysis of ATP that leads to ATP depletion can be both beneficial in different disease models and serve as potential therapeutics.

We used *in-silico* screening of natural compounds to look for potential replacements of ATPIF1 that inhibit hydrolysis. Revisiting natural products for drug discovery is increasingly powerful as, historically, natural products were the source of virtually all medicinal preparations, and they continue to enter clinical trials. Compound screening identified the catechin family as a group of compounds that could potentially bind ATP synthase and regulate its activity. Despite the long list of beneficial effects of Catechins and Epicatechins in mitochondrial biology, which was recently reviewed (Daussin *et al*, 2021), no molecular mechanism has been proposed for the improved mitochondrial function observed.

By comparing the effect of catechin and EPI, we have shown that EPI, but not Catechin (Appendix Fig S3), can inhibit ATP hydrolysis without decreasing the activity of other respiratory complexes. Moreover, using *in silico* analyses and *in vitro* competition assays,

© 2023 The Authors

we have identified the molecular target of EPI on mitochondria: the ATPIF1 binding site on ATP synthase. Structural, biochemical, and functional data implicate EPI as a partial mimic of ATPIF1, competing for the same binding site on ATP synthase. The partial mimicry comes from the fact that like ATPIF1, EPI inhibits hydrolytic activity by ATP synthase, but unlike ATPIF1, EPI does not impair its synthesis activity.

ATP synthase inhibitors such as BTB06584, oligomycin, aurovertin, and BMS-199264 were shown in the literature to reduce ATP depletion during heart and cardiac cell ischemia and reperfusion injury, where nonselective (both synthesis and hydrolysis) inhibition was observed for oligomycin and aurovertin (Grover *et al*, 2004; Grover & Malm, 2008; Ivanes *et al*, 2014). Knowing the mechanism and pharmacokinetics of the drug selected is essential when considering therapeutic approaches. For the above-mentioned compounds, pharmacokinetics is unknown.

We show that mitochondrial consumption of cytosolic ATP is a pathological compensation in different mitochondrial disease models. A decrease in total ATP content, together with an increase in maximal hydrolytic capacity was observed in mitochondrial diseases of CIII (BCS1L) and CV (ATP6) deficiencies. Similarly, in a mouse model of muscular dystrophy, the catalytic reversal of the ATP synthase is observed after eccentric injury. These changes may represent the adaptation of ATP synthase to different conditions, including those that induce cellular stress. Failure of ATP synthase to adequately adapt is already associated with disease (Daum *et al*, 2013; Johnson *et al*, 2019). Moreover, the hydrolytic adaptation can create a hysteretic consumption of cytosolic ATP and in this scenario, the regulation of ATP synthase activity by the inhibitory protein ATPIF1 is fundamental (Garcia-Bermudez & Cuezva, 2016).

Furthermore, our data show that the CIII-mutant fibroblasts, which harbor the largest decrease in ATPIF1 bound to CV and the highest increase in ATP hydrolytic activity, are also the most responsive to EPI treatment. Accordingly, these CIII-deficient fibroblasts, caused by a mutation in BCS1L showed the best improvement in total ATP content and cellular proliferation rates among the different mutant fibroblast lines treated with EPI. This result is in line with a recent yeast screen showing that mutations in ATP synthase that reduce ATP hydrolytic capacity can compensate for BCS1L mutations (Ostojic *et al*, 2013).

Muscular dystrophies cause a chronic increase in intracellular calcium, which induces mitochondrial calcium overload (Burr & Molkentin, 2015; Law *et al*, 2020; Mareedu *et al*, 2021; Zabłocka *et al*, 2021). The impairment in mitochondrial function and number induced by calcium overload prevents adequate repair and regeneration of myofibers (Kelly-Worden & Thomas, 2014; Vila *et al*, 2017; Moore *et al*, 2020; Ramos *et al*, 2020). This can lead to a vicious cycle in which tears in the sarcolemma cause high-intracellular calcium, which impairs mitochondrial function, preventing repair/regeneration and further prolonging muscle damage. Furthermore, proteomics and imaging approaches have confirmed that mitochondria are early responders to myofiber injury and are functionally imperative for repair and regeneration (Sharma *et al*, 2012; Vila *et al*, 2017). Our results in *mdx* mice show that ATP hydrolysis inhibition by EPI is sufficient to partially restore muscle force. In addition, we observed that EPI treatment can reverse some of the mitochondrial changes induced by calcium overload, such as OPA1 cleavage.

Therapeutic approaches to address mitochondrial disease have been limited, partly a reflection of the complex and not yet fully understood biology of these organelles. Most therapeutics to date have focused on activators of mitochondrial gene transcription, mitochondrial protective drugs, antioxidants, and inhibitors of the mitochondrial permeability transition pore (Viscomi & Zeviani, 2020). Here, we propose that under conditions where an impaired respiratory function cannot be restored, selective inhibition of CV hydrolytic activity, on its own, is sufficient to preserve the ATP pool, and improve cellular and tissue function.

Materials and Methods

Animals

D2.*mdx*, DBA2/J, C57BL/10ScSn.Dmd<*mdx*>/J (*mdx*) and C57BL/10ScSn/J mice were purchased from Jackson laboratories. For the EPI treatment study, mice were treated orally twice daily, 12 h apart, from doses ranging from 0.5 to 15 mg/kg/day. After 13 days of treatment, mice underwent muscle function testing and susceptibility to eccentric injury was determined, as described below. The following day, 1 h after the final dose, the mice were euthanized, and tissues were collected. All animal protocols were approved by the Animal Care and Use Committee at the University of Maryland School of Medicine. Samples were not blinded.

Hindlimb force measurements and susceptibility to injury

Maximal force production of the plantar flexor muscle group was measured *in vivo* with a 305C muscle lever system (Aurora Scientific Inc., Aurora, Canada), as previously described (Khairallah *et al*, 2012; Call & Lowe, 2016). Animals were anesthetized via inhalation (~2% isoflurane, SomnoSuite, Kent Scientific), placed on a thermostatically controlled table with anesthesia maintained via nose-cone (~2% isoflurane), the knee fixed with a pin pressed against the tibial head, and the foot firmly fixed to a footplate on the motor shaft. Contractions were elicited by percutaneous electrical stimulation of the tibial nerve (0.2 ms pulse, 500 ms train duration) at increasing frequencies. Following assessment of isometric torque, susceptibility to injury was assayed with 25 eccentric contractions as previously described (Khairallah *et al*, 2012; Call & Lowe, 2016) at maximal isometric torque (150 mS duration, 0.2-mS pulse train at 150 Hz). Eccentric contractions were achieved by translating the footplate 38° backward at a velocity of 800°/S after the first 100 mS of the isometric contraction. The decrease in the peak isometric force before the eccentric phase was taken as an indication of muscle damage.

Evans Blue Dye detection

Evans Blue Dye (EBD) detection was used as previously described as an *in vivo* marker of muscular fiber damage (Hamer *et al*, 2002). Briefly, mice were injected intravenously with 1% EBD (Sigma, St Louis, MO, USA) (w/v) in phosphate-buffered saline (PBS, pH 7.5), 24 h before they were euthanized. Tissues were harvested, fixed, and imaged as described (Hamer *et al*, 2002).

© 2023 The Authors

Tissue homogenization

Mouse gastrocnemius muscles were homogenized in 0.75 ml MAS (70 mM Sucrose, 220 mM Mannitol, 5 mM KH_2PO_4, 5 mM $MgCl_2$, 1 mM EGTA, 2 mM HEPES; pH 7.2) using a glass–glass Dounce homogenizer. Thirty strokes with the tight pestle were applied per sample. The homogenate was centrifuged at 1,200 × *g* to pellet non-broken material and the supernatant was stored at −80°C until further use.

Cell culture

Primary fibroblasts derived from patients were acquired from Telethon Network Genetic Biobank ($NDUFA6^{A178P/c.420+784C>T}$, $P1{:}BCS1L^{R73C/F368I}$, $P2{:}BCS1L^{R183C/R184C}$, $P1{:}TTC19^{L219X}$, $TMEM70^{Q197X}$), Coriell Institute for Medical Research ($ATP6^{L156R}$), and Control and $MIC26^{I117T}$ (Beninca *et al*, 2021). Cells were maintained in EMEM (Eagle's Minimum Essential Medium; ATCC #30-2003) and supplemented with 15% FBS and antibiotic–antimycotic (ThermoFisher, #15240062) in humidified atmosphere at 37°C and 5% CO_2. During the treatment with the different compounds, media was changed to phenol red-free DMEM (Dulbecco's Modified Eagle Medium; ThermoFisher #A1443001) containing 4.5 g/l glucose or galactose supplemented with 2 mM glutamine, 1 mM sodium pyruvate, 10% FBS, and antibiotic–antimycotic. Compounds were prepared in DMSO and added directly to the growth media at the specified concentration. Lentiviral infection of pLentiATPIF1-WT and pLentiATPIF1-H49K (produced by Welgen, Inc) was performed using 8 µg/ml polybrene in EMEM for 30 h and positive selection performed by 1 µg/ml puromycin.

Immortalized myoblasts derived from a healthy control and a DMD patient (DMD KM1328, mutation in exon 52 of DMD gene) were grown in Skeletal Muscle Growth Medium (Promocell) containing 1% penicillin–streptomycin at 37°C with 5% CO_2 (Mamchaoui *et al*, 2011). Upon reaching 100% confluency, the medium was replaced with Skeletal Muscle Differentiation Medium (Promocell) containing 1% penicillin–streptomycin.

Cellular proliferation rates

To measure cellular proliferation, fibroblasts were plated at 1,000 cells/well in duplicated 96-well imaging plates 98 h prior to the assay. Twenty-four hours after plating, one plate was used for imaging of Hoechst staining (1 µg/ml) for cell counting. The second plate was treated with 50 nM (+)-Epicatechin or DMSO and kept for 72 h until cell counting. Imaging was performed in triplicates with a Perkin Elmer Operetta system (10× objective). Analysis was performed with Perkin Elmer Harmony (Harmony 4.1) software and % change from plate 1 to plate 2 was calculated for the respective cells and treatments.

Membrane stability assay in DMD cells

The osmotic shock base solution was prepared using 5 mM HEPES, 5 mM KCl, 1 mM $MgCl_2$, 5 mM NaCl, 1.2 mM $CaCl_2$, and 1 mM glucose in DIW. Sucrose was added to the base solutions to reach 45 milliosmole (mosmol). The actual osmolarity was determined using a VAPRO vapor pressure osmometer (Wescor Inc.) Myotubes in 24-well plates were treated for 1 and 24 h at days 4–5 of differentiation. On day 5 of differentiation, the cells were subjected to 20 min of osmotic shock at 37°C using 45 mosmol solution. The supernatant was collected and centrifuged to separate cell debris. Adherent cells were trypsinized and pelleted before lysis in 50 µl of DIW and 3 freeze–thaw cycles. The Creatine Kinase Assay (Sekisui Diagnostics) was used to measure creatine kinase (CK) levels in both the supernatant and lysate fractions. In a 96-well plate, 8 µl of lysate or 12 µl of supernatant with 140 µl of reagent was loaded per well in triplicate. The U/l of CK was calculated as follows: (mOD/min) (total volume in ml) (dilution factor)/(6.22 M^{-1} cm^{-1}) (light path in cm) (sample volume in ml). The percent CK release was calculated as follows: $CK_{extracellular}/(CK_{extracellular} + CK_{intracellular}) \times 100$.

Mitochondrial isolation from the heart

Mice were anesthetized with isoflurane followed by a cervical dislocation, the heart was immediately removed and placed in ice-cold relaxation buffer (5 mM sodium pyrophosphate, 100 mM KCl, 5 mM EGTA, and 5 mM HEPES; pH 7.4). The heart was squeezed with tweezers to remove blood, minced with scissors, and then placed in a glass–glass Dounce homogenizer with 3 ml of HES homogenization buffer (250 mM sucrose, 5 mM HEPES, and 1 mM EDTA; pH to 7.2, adjusted with KOH). The heart tissue was homogenized with 10 strokes with the loose pestle and 15 strokes with the tight pestle. The homogenized tissue was placed in a prechilled 15 ml conical tube and centrifuged at 900 × *g* (4°C) for 10 min. The supernatant was removed, placed in a new tube, and centrifuged again at 900 × *g* for 10 min. The supernatant was then transferred to 2 ml microcentrifuge tubes and centrifuged at 10,000 × *g* (4°C) for 10 min. The mitochondrial pellets were resuspended in ice-cold HES buffer and mitochondrial protein was measured with a BCA assay (Pierce). The concentrated mitochondrial pellet was stored on ice.

Bovine Complex V preparations

Purification of mammalian F-type ATP synthase was conducted as previously described (Urbani *et al*, 2019) with slight modifications. Fresh *B. taurus* hearts were obtained immediately after slaughter by an authorized slaughterhouse and fat and connective tissues were carefully removed allowing the preparation of 1,000 g of minced meat. Each 500 g was suspended in 3,250 ml of 23 mM sodium phosphate buffer (pH 7.4 at 0°C) and homogenized for 5 min at 13,000 rpm in a homogenizer (Nihon Seiki), followed by centrifugation for 20 min at 1,000 × *g* in a refrigerated centrifuge (Kubota Model 9810; RS-6600 rotor). The precipitate was suspended in 3,375 ml of 22.2 mM sodium phosphate buffer (pH 7.4) and rehomogenized, followed by centrifugation for 20 min at 1,000 × *g*. Supernatants were then combined and centrifuged for a further 30 min at 8,000 × *g* with a refrigerated centrifuge (Beckman Model Avanti HP-30I) using a JLA-10.500 rotor. The precipitate was then suspended in 50 mM Tris–HCl buffer (pH 8.0) and pelleted for 30 min at 22,000 × *g* with an ultracentrifuge (Beckman Model-7) using a 45 Ti rotor. The pellet was suspended in 50 mM Tris–HCl buffer (pH 8.0) containing 660 mM sucrose to a final protein concentration of ~23 mg/mL. The suspension was kept in a 40 mM HEPES buffer (pH 7.8) containing 2 mM $MgCl_2$, 0.1 mM EDTA, and

© 2023 The Authors

0.1 mM DTT and solubilized on ice via slow addition of deoxycholate and decyl-maltoside to final concentrations of 0.7% (wt/vol) and 0.4% (wt/vol), respectively. The suspension was then centrifuged at 176,000 × *g* for 50 min and the supernatant was applied to a sucrose step gradient (40 mM HEPES pH 7.8, 0.1 mM EDTA, 0.1 mM DTT, 0.2% wt/vol decyl-maltoside and 2.0 M, 1.1 M, 1.0 M, or 0.9 M sucrose) and centrifuged at 176,000 × *g* for 15.5 h. Fractions exhibiting ATP hydrolysis activity were loaded onto a Poros-20HQ ion-exchange column. The detergent was exchanged to LMNG using a double gradient from 0.2 to 0% decyl-maltoside and 0–0.05% LMNG for 80 min at 1 ml/min. Complexes were eluted by a linear concentration gradient of 0–240 mM KCl in 40 mM HEPES pH 7.8, 150 mM sucrose, 2 mM $MgCl_2$, 0.1 mM EDTA, 0.1 mM DTT and 0.005% (wt/vol) LMNG. For preparation of monomer and tetramer fractions, ion-exchange chromatography was replaced by an additional overnight sucrose density gradient ultracentrifugation step (176,000 × *g* for 15.5 h) conducted using either 0.05% LMNG (monomer) or 0.05% GDN (tetramer) as gradient detergents.

Respirometry in intact cells

Primary fibroblasts were plated at 6,000 cells/well 48 h prior to the assay. Twenty-four hours after plating, standard maintenance media was replaced with high glucose (4.5 g/l) DMEM supplemented with 2 mM glutamine, 1 mM sodium pyruvate, 10% FBS, and antibiotic–antimycotic, and cells were treated with 50 nM (+)-Epicatechin. On the day of the assay, the medium was replaced with assay medium composed of DMEM (Sigma #D5030) with 5 mM HEPES, pH 7.4 supplemented with 2 mM glutamine, and 1 mM sodium pyruvate. OCR and ECAR were measured in a SH XF96 analyzer under basal conditions as well as after injection of 2 μM oligomycin, two sequential additions of 1.5 μM FCCP, followed by 1 μM rotenone with 2 μM Antimycin A (AA). Respiratory parameters were calculated according to standard protocols (Divakaruni *et al*, 2014), and all rates were corrected for nonmitochondrial respiration/background signal by subtracting the OCR insensitive to rotenone plus AA.

Respirometry in isolated mitochondria and homogenates

Isolated mitochondria

Heart mitochondria (0.75–1.5 μg) were loaded into an Agilent Seahorse XF96 microplate in 20 μl of MAS (70 mM Sucrose, 220 mM Mannitol, 5 mM KH_2PO_4, 5 mM $MgCl_2$, 1 mM EGTA, 2 mM HEPES; pH 7.2) plus 0.1% free fatty acid BSA containing substrates. The loaded plate was centrifuged at 2,000 × *g* for 5 min at 4°C (no brake) and an additional 130 μl of MAS was added to each well. When assessing compounds' effect on respirometry, the compounds were added at this point at the indicated concentration in MAS buffer. To avoid disrupting mitochondrial adherence to the bottom of the plate, MAS was added using a multichannel pipette pointed at a 45° angle to the top of the well-chamber, as instructed by the manufacturer. Substrate concentrations in the well when assay was starting in State 4 were as follow: 5 mM pyruvate + 5 mM malate. Substrate concentrations in the well when assay was starting in State 3 were as follow: (i) 5 mM pyruvate + 5 mM malate + 4 mM ADP. Injections were performed as indicated in the figure panels at the following final concentration in the well: oligomycin (3.5 μM), FCCP (4 μM), ATP (20 mM), Antimycin A (AA)(2 μM). Compounds were added at the indicated concentration in MAS after spinning the plate and before lacing it inside the XF96 equipment.

Tissue homogenates

Respirometry in previously frozen samples was performed as described (Osto *et al*, 2020; Acin-Perez *et al*, 2020a) in MAS containing 100 μg/ml of cytochrome c. For respiratory assays, we loaded 8 μg of total mouse gastrocnemius homogenates.

ATP hydrolysis in previously frozen samples (HyFS)

ATP hydrolysis capacity or State 4 acidification rates were measured using Agilent Seahorse XF96 as described (Fernandez-del-Rio *et al*, 2023). Briefly, plates were loaded with 0.75–1.5 μg of mouse heart mitochondria, 20 μg of mouse gastrocnemius homogenate, or 25 μg of cell lysate diluted in MAS (70 mM Sucrose, 220 mM Mannitol, 5 mM KH_2PO_4, 5 mM $MgCl_2$, 1 mM EGTA, 2 mM HEPES; pH 7.2). Cell lysates were prepared by subjecting the samples to 4 cycles of free-thaw (liquid nitrogen-37°C water bath) before measuring protein concentration. Initial respiration of the samples was sustained by the addition of 5 mM succinate + 2 μM rotenone in the MAS after centrifugation. Injections were performed as indicated in the figure panels at the following final concentration in the well: Antimycin A (AA) (2 μM), oligomycin (5 μM), FCCP (1 μM), ATP (20 mM). To assess maximal ATP concentration, ATP was injected consecutively.

Determination of ATP synthesis versus hydrolysis capacity in intact mitochondria

Quantification of the ratio of hydrolysis/synthesis in heart mitochondria fueled with either pyruvate plus malate or succinate plus rotenone (Succ+Rot) was determined by calculating the ratio between the ECAR rate on mitochondria started in state 4 after ATP injection (hydrolysis) and the basal OCR rate of mitochondria started in State 3 (synthesis) before any port injection.

Mitochondrial content

To determine mitochondrial mass, muscle homogenate (8 μg) in 20 μl of MAS (70 mM Sucrose, 220 mM Mannitol, 5 mM KH_2PO_4, 5 mM $MgCl_2$, 1 mM EGTA, 2 mM HEPES; pH 7.2) was placed in a clear-bottom 96-well microplate. Then, 130 μl of a 1:2,000 dilution of Mitotracker Deep Red FM (MTDR, ThermoFisher), a mitochondrial selective dye insensitive to weak or mild changes in membrane potential, was added and incubated for 10 min at 37°C. Plates were centrifuged at 2,000 × *g* for 5 min at 4°C (no brake), and supernatant was carefully removed. Finally, 100 μl of PBS was added per well and MTDR fluorescence measured ($\lambda_{excitation}$ = 625 nm; $\lambda_{emission}$ = 670 nm). Mitochondrial content was calculated as MTDR signal (minus blank) per microgram of protein as previously described (Acin-Perez *et al*, 2020a).

Immunofluorescence and proximity ligation assay (PLA)

Cells were fixed with 4% paraformaldehyde in phosphate-buffered saline (PBS) for 20 min at RT, permeabilized (0.1% Triton X-100,

© 2023 The Authors

0.05% sodium deoxycholate in PBS), blocked, and stained with primary and secondary antibodies in blocking solution (5% donkey serum). Titration of primary antibodies dilution (1:5,000) was made for visualization of spaced PLA dots. Saturation of antibodies was avoided. Coverslips were mounted with Mowiol. For PLA, cells were plated on coverslips and then fixed, permeabilized, and blocked as described above. After that, we followed the manufacturer's protocol using the Duolink *In Situ* Red Starter Kit Mouse/Rabbit (Sigma Aldrich). After the last wash step, coverslips were incubated overnight with described primary antibodies (see below) for immunofluorescence. Image acquisition was performed using Zeiss LSM880 Confocal system with Airyscan equipped with a Zeiss Pla-Apochromat 40×/1.2 and 63×/1.4 N.A. objective. 3D image stacks were acquired at optimal Z-distance and reconstructed using Zen black software. Quantification of PLA dots and mitochondria volume was performed in aleatorily acquired images using Aivia v.10 software. Pixel classification was used to segment mitochondria and PLA dots. Only PLA dots localizing in mitochondria were counted.

Total ATP content

Cells were washed twice with PBS and lysed using 0.5% Triton-X100 in PBS. After 3 min centrifugation at 11,000 × *g*, 5 µl of total lysate was used in triplicates for assessment of total ATP content using the ATP Determination Kit (#A22066 Thermo Fisher) and following the manufacturer's instructions. Luminescence was monitored at ~560 nm using a Tecan Spark luminometer.

Mitochondrial ATP content

Mitochondrial ATP content was assessed with the BioTracker™ ATP-Red dye, a live cell red fluorescent imaging probe for ATP. The probe specifically reports ATP content in the mitochondrial matrix of living cells. The probe without ATP forms a closed ring structure that is not fluorescent. In the presence of the negatively charged ATP, the covalent bonds between boron and ribose in the probe are broken and the ring opens, causing the probe to be fluorescent.

Cells were incubated for 1 h with 200 nM MTG and 1 µg/ml Hoechst, washed twice with PBS, and incubated for 15 min with 5 µM BioTracker™ ATP-red dye (Millipore) in medium at 37°C (5% CO_2).

Before imaging, the cells were washed twice with medium and fresh medium was added. Imaging was performed in triplicates and in two focus planes with a Perkin Elmer Operetta system (20× objective) at 37°C and 5% CO_2. Excitation and emission filters used for the combination of dyes: Hoechst (ex. 360–400, em. 410–480), MTG (ex. 460–490, em. 500–550) and mtATP Red (ex. 560–580, em. 590–640). Analysis was performed with Perkin Elmer Harmony (Harmony 4.1) software by measuring the average ATP red fluorescence intensity inside a region of interest (Filimonov *et al*, 2014) generated by the MTG area.

Membrane potential determination

Cells were incubated for 1 h with 200 nM MTG, 200 nM TMRE (Biotium) and 1 µg/ml Hoeschst, washed twice with PBS, and incubated with 200 nM in medium at 37°C (5% CO_2). Imaging was performed in triplicates and in two focus planes with a PerkinElmer Operetta CLS high-content system (20× objective) at 37°C and 5% CO_2. Analysis was performed with Perkin Elmer Harmony (Harmony 4.1) software by measuring mean TMRE fluorescence intensity inside an ROI generated by MTG area.

Lactate

1 × 10^6 cells were plated and treated for 24 h in the described media supplemented with 10% dialyzed FBS. Cells were fixed and media was frozen at −80°C until further processing. Media was thawed on ice and diluted 1:70 in PBS, 5 µl was used in triplicates for assessment of lactate using the Lactate-Glo Assay (#J5021 Promega) following manufacturer's instructions. Luminescence was recorded using a Tecan Spark luminometer.

Protein gel electrophoresis and immunoblotting

SDS-PAGE
Fifteen to thirty microgram protein from total tissue homogenates or cell lysates in RIPA buffer were loaded into 4–12% Bis-Tris precast gels (ThermoFisher Sci. NP0321) and gel electrophoresis was performed in xCell SureLock Mini-Cells (Novex), under constant voltage of 80 V for 15 min and 120 V for 60 min.

Blue native gel electrophoresis
Mitochondria derived from tissues were permeabilized with either 8 mg digitonin/mg protein (heart) or 3 mg digitonin/mg protein (gastrocnemius). Digitonin incubation was performed on ice for 5 min and then centrifuged at 20,000 × *g* for 30 min as previously described (Acin-Perez *et al*, 2008, 2020a). Cells preparations for BN-PAGE were performed as described (Fernandez-Vizarra & Zeviani, 2021). Supernatant containing mitochondrial complexes and super complexes were mixed with Blue Native sample buffer (5% Blue G dye in 1 M 6-amiohexanoic acid), loaded, and run on a 3–12% native precast gel (Invitrogen). Gels were run till the blue front ran out of the gel and gel was transferred to PVDF membranes.

Immunoblotting
Proteins were transferred to methanol-activated PVDF membrane in xCell SureLock Mini-Cells under 45 V constant voltage for 1–1.5 h at 4°C. Coomassie was completely washed off of blue native blots using 100% methanol. Blots were blocked with 3% BSA in PBS-T (1 ml/l Tween-20/PBS) and incubated with primary antibody diluted in 1% BSA/PBST overnight at 4°C. Primary antibodies used are listed below. The next day, blots were washed 3 × 10 min in PBS-T, probed with IRDye 680RD or HRP conjugated secondary antibodies diluted in blocking solution for 1 h at room temperature, and rinsed again 3 × 10 min in PBS-T. Detection was achieved using ChemiDoc Molecular Imager (BioRad). Band densitometry was quantified using ImageJ Gel Plugin (NIH).

Antibodies

Mouse monoclonal anti-ATP5A (1:1,000; #439800; Thermo Fisher Scientific), Mouse monoclonal anti-SDHA (1:1,000, #459200, Thermo Fisher Scientific), Mouse monoclonal anti-SDHB (1:1,000, #ab14714, Abcam), Rabbit polyclonal anti-UQCRC2 (1:1,000, #14742-1-AP; ProteinTech), Rabbit polyclonal anti-ATPIF1 (1:1,000,

© 2023 The Authors

#13268S; Cell Signaling Technology), Rabbit monoclonal anti-TOMM20 Alexa Fluor 488 (1:1,000; #ab205486; Abcam), Mouse monoclonal anti-Vinculin (1:1,000, #V9131, Sigma Aldrich), Mouse monoclonal anti-OPA1 (1:1,000, #612606, BD Transduction Laboratories), and Mouse monoclonal anti-cytochrome c (1:5,000, #ab110325, Abcam).

Complex V in gel activity

Heart permeabilized mitochondria (20 μg) were run on a 3–12% native precast gel (Invitrogen). CV in gel activity was performed as previously described (Acin-Perez *et al*, 2020b). CV in gel Activity was stopped in 50% methanol. Finally, gels were stained with Coomassie to correct for loading. Imaging was performed at different stages: after 3 h and O/N In Gel Activity, after fixing with 50% methanol and after Coomassie staining.

ATPIF1 binding assays

Recombinant ATPIF1 was added to bovine mitochondrial CV tetramer or isolated mouse heart mitochondria at a protein/protein ratio 1/5, 1/25, or 1/50 in MAS. Binding assay was performed at RT for 10 min. Samples were then prepared to be loaded in Native gels. Ten micrograms of CV tetramer or isolated mitochondria were used and recombinant ATPIF1 at the indicated ratio was added.

Cytochrome c release measurements

Cytochrome c (Cyt c) release was measured in mitochondrial supernatants of gastrocnemius homogenates. Fifty micrograms of gastrocnemius homogenate were centrifuged at 10,000 × *g* for 10 min. Supernatant was collected and cytochrome c levels were measured by western blot and ELISA (R&D Systems) using 10 and 3 μg of supernatant, respectively.

Catechin docking analysis

Ligands with molecular weight between 80 and 800 kDa were filtered from CoconutDB database (https://coconut.naturalproducts.net/) (Sorokina *et al*, 2021) using mongoDB. To reinforce the potential mitochondrial activity, PASS analysis (Filimonov *et al*, 2014) was used for prediction of the three-biological activities: creatine kinase inhibitor, antioxidant, and free radical scavenger. *In-situ* ligand screening was carried out using Autodock Vina 4.2 (Trott & Olson, 2010) against the bovine ATP synthase (PDB: 6ZQN) as a receptor. PYMOL structure viewer (https://pymol.org/2/) was used for visualization and selection of coordinates. The selected region contains the F1 head of the ATP synthase, more specifically with the Alpha and Beta subunits around the central stalk, where ATPIF1 is possibly interacting with the complex (Bason *et al*, 2011).

Structure preparation and binding site exploration

All simulations for the interaction of (+)- and (−)-Epicatechin, quercetin and (−)-catechin with F1-ATP synthase with ATPIF1 were started from the following x-ray structure Protein Data Bank ID: 1ohh (Cabezón *et al*, 2003). Structures of the epicatechins, quercetin and (−)-catechin were retrieved from Pubchem (CID: 182232: (+)-Epicatechin; 72276: (−)-Epicatechin; 5280343: quercetin; 73160: (−)-catechin). Structures were prepared using YASARA (Land & Humble, 2018), adding hydrogen atoms, checking the protonation states of side chains, and optimizing the hydrogen-bond network. If necessary, loops were closed using YASARA. Simulations were carried out with ATPIF1 present and absent. PELE (Protein Energy Landscape Exploration) webserver was used to identify potential epicatechin binding sites on F1-ATP synthase. PELE is a Monte Carlo (MC)-based technique that consists of three main steps, i.e., ligand and protein perturbation; side chain sampling; and minimization (Borrelli *et al*, 2005, 2010). Resulting poses were analyzed for ligand affinities and energies using AutoDock Vina (Trott & Olson, 2010). Visualizations were analyzed using UCSF Chimera (Pettersen *et al*, 2004).

Principal component analysis

Principal component analysis was carried out in R studio (R version 4.1.1) using Singular value decomposition to determine the principal components and the eigenvectors for the analyzed variables.

Statistical analysis

Statistical analysis was performed with GraphPad Prism® 9.01 using one-way, two-way analysis of variance (ANOVA) or pairwise comparison as indicated in the figure legends. Corrections for multiple comparisons were made by Šídák's or Dunnett's multiple comparisons when appropriate. Differences were considered statistically different at $P < 0.05$. Statistical significance is denoted by *P*-value in each figure. Individual points in a graph denote individual cell preparations, mouse samples, or biological replicates.

Data availability

This study deposits no data in external repositories.

Expanded View for this article is available online.

Acknowledgements

The authors would like to acknowledge Dani Dagan and Ajit S. Divakaruni for their critical reading of the manuscript and valuable discussion. GFP-ATPIF1 protein was a kind gift from Dr. Toshiharu Suzuki. This work was supported by National Institutes of Health grants: CURE: DDRC NIH-NIDDK P30 DK041301 (ML), UCLA/UCSD DERC NIH-NIDDK: P30 DK063491 (ML), R01 DK099618-05 (OSS), R01 CA232056-01 (OSS), R21AG060456-01 (OSS), R21 AG063373-01 (OSS), ADA Grant No. 1-19-IBS-049 (OSS), R01 AA026914 (ML, OSS) and Seed award from DGSOM at UCLA (ML). Work was also supported by AMED BINDS No. JP16K07266; MEXT No. 17H03647 (CG), Naito Foundation Subsidy for Female Researchers after Maternity Leave, MEXT No. 19K15749; JSPS No. 20J40167 (CJ) and Epirium Bio Inc (RM, CG (b), KE, GS and SD).

Author contributions

Orian S Shirihai: Conceptualization; resources; supervision; funding acquisition; writing – review and editing. **Rebeca Acin-Perez:** Conceptualization; formal analysis; supervision; validation; investigation; methodology; writing – original draft; writing – review and editing. **Cristiane Benincá:**

© 2023 The Authors

Conceptualization; resources; supervision; investigation; methodology; writing – original draft; writing – review and editing. **Lucia Fernandez del Rio:** Conceptualization; validation; investigation; methodology; writing – original draft; writing – review and editing. **Cynthia Shu:** Validation; investigation. **Siyouneh Baghdasarian:** Validation. **Vanessa Zanette:** Funding acquisition; investigation. **Christoph Gerle:** Resources; funding acquisition. **Chimari Jiko:** Methodology. **Ramzi Khairallah:** Resources; investigation. **Shaharyar Khan:** Resources; software; investigation. **David Rincon Fernandez Pacheco:** Validation; investigation. **Byourak Shabane:** Investigation. **Karel Erion:** Resources; funding acquisition. **Ruchi Masand:** Funding acquisition. **Sundeep Dugar:** Funding acquisition. **Cristina Ghenoiu:** Funding acquisition. **George Schreiner:** Funding acquisition. **Linsey Stiles:** Conceptualization; resources; supervision; funding acquisition; investigation; methodology; writing – original draft; writing – review and editing. **Marc Liesa:** Conceptualization; resources; supervision; writing – review and editing.

In addition to the CRediT author contributions listed above, the contributions in detail are: Conceptualization: RA-P, CB, LF, LS, ML, OSS; Methodology: RA-P, CB, LF, VZ, RK, SK, DRF, LS; Validation: RA-P, CB, LF, LS; Investigation: RA-P, CB, LF, CS, SB, VZ, BS, RK, SK, LS; Writing–Original Draft: RA-P, CB; Writing–Review and Editing: RA-P, CB, LF, CS, VZ, CG, CJ, RK, SK, DRF, RM, LS, ML, OSS; Funding Acquisition: VZ, CG, SK, RM, ML, and OSS; Resources: CB, VZ, CG, CJ, RK, SK, DRF, RM, LS, ML, OSS; Supervision: RA-P, CB, RM, ML, OSS. All the authors read and approved the final version of the manuscript.

Disclosure and competing interests statement

RM, CG (b), KE, GS, and SD were employees of Epirium Bio when this study was conducted. ML and OSS are cofounders and consultants of Enspire Bio LLC. OSS is a cofounder and SAB member of Senergy-Bio and Capacity-Bio, and when this study was conducted, he has been serving as a consultant or collaborator to LUCA-Science, IMEL, Epirium, Johnson & Johnson, Pfizer and Stealth Biotherapeutics.

References

Acin-Perez R, Fernandez-Silva P, Peleato ML, Perez-Martos A, Enriquez JA (2008) Respiratory active mitochondrial supercomplexes. *Mol Cell* 32: 529 – 539

Acin-Perez R, Benador IY, Petcherski A, Veliova M, Benavides GA, Lagarrigue S, Caudal A, Vergnes L, Murphy AN, Karamanlidis G *et al* (2020a) A novel approach to measure mitochondrial respiration in frozen biological samples. *EMBO J* 39: e104073

Acin-Perez R, Hernansanz-Agustin P, Enriquez JA (2020b) Analyzing electron transport chain supercomplexes. *Methods Cell Biol* 155: 181 – 197

Bason JV, Runswick MJ, Fearnley IM, Walker JE (2011) Binding of the inhibitor protein IF_1 to bovine F_1-ATPase. *J Mol Biol* 406: 443 – 453

Beninca C, Zanette V, Brischigliaro M, Johnson M, Reyes A, Valle DAD, J Robinson A, Degiorgi A, Yeates A, Telles BA *et al* (2021) Mutation in the MICOS subunit gene APOO (MIC26) associated with an X-linked recessive mitochondrial myopathy, lactic acidosis, cognitive impairment and autistic features. *J Med Genet* 58: 155 – 167

Blaauw B, Agatea L, Toniolo L, Canato M, Quarta M, Dyar KA, Danieli-Betto D, Betto R, Schiaffino S, Reggiani C (2010) Eccentric contractions lead to myofibrillar dysfunction in muscular dystrophy. *J Appl Physiol* 108: 105 – 111

Bonilla E, Samitt CE, Miranda AF, Hays AP, Salviati G, DiMauro S, Kunkel LM, Hoffman EP, Rowland LP (1988) Duchenne muscular dystrophy: deficiency of dystrophin at the muscle cell surface. *Cell* 54: 447 – 452

Borrelli KW, Vitalis A, Alcantara R, Guallar V (2005) PELE: protein energy landscape exploration. A novel Monte Carlo based technique. *J Chem Theory Comput* 1: 1304 – 1311

Borrelli KW, Cossins B, Guallar V (2010) Exploring hierarchical refinement techniques for induced fit docking with protein and ligand flexibility. *J Comput Chem* 31: 1224 – 1235

Budzinska M, Zimna A, Kurpisz M (2021) The role of mitochondria in Duchenne muscular dystrophy. *J Physiol Pharmacol* 72:

Bulfield G, Siller WG, Wight PA, Moore KJ (1984) X chromosome-linked muscular dystrophy (mdx) in the mouse. *Proc Natl Acad Sci USA* 81: 1189 – 1192

Burr AR, Molkentin JD (2015) Genetic evidence in the mouse solidifies the calcium hypothesis of myofiber death in muscular dystrophy. *Cell Death Differ* 22: 1402 – 1412

Cabezón E, Montgomery MG, Leslie AG, Walker JE (2003) The structure of bovine F1-ATPase in complex with its regulatory protein IF1. *Nat Struct Biol* 10: 744 – 750

Call JA, Lowe DA (2016) Eccentric contraction-induced muscle injury: reproducible, quantitative, physiological models to impair skeletal Muscle's capacity to generate force. *Methods Mol Biol* 1460: 3 – 18

Campanella M, Casswell E, Chong S, Farah Z, Wieckowski MR, Abramov AY, Tinker A, Duchen MR (2008) Regulation of mitochondrial structure and function by the F1Fo-ATPase inhibitor protein, IF1. *Cell Metab* 8: 13 – 25

Campanella M, Parker N, Tan CH, Hall AM, Duchen MR (2009) IF_1: setting the pace of the F_1F_o-ATP synthase. *Trends Biochem Sci* 34: 343 – 350

Chen WW, Birsoy K, Mihaylova MM, Snitkin H, Stasinski I, Yucel B, Bayraktar EC, Carette JE, Clish CB, Brummelkamp TR *et al* (2014) Inhibition of ATPIF1 ameliorates severe mitochondrial respiratory chain dysfunction in mammalian cells. *Cell Rep* 7: 27 – 34

Chinopoulos C, Adam-Vizi V (2010) Mitochondria as ATP consumers in cellular pathology. *Biochim Biophys Acta* 1802: 221 – 227

Classen JB, Mergner WJ, Costa M (1989) ATP hydrolysis by ischemic mitochondria. *J Cell Physiol* 141: 53 – 59

Connolly NMC, Theurey P, Adam-Vizi V, Bazan NG, Bernardi P, Bolanos JP, Culmsee C, Dawson VL, Deshmukh M, Duchen MR *et al* (2018) Guidelines on experimental methods to assess mitochondrial dysfunction in cellular models of neurodegenerative diseases. *Cell Death Differ* 25: 542 – 572

Daum B, Walter A, Horst A, Osiewacz HD, Kuhlbrandt W (2013) Age-dependent dissociation of ATP synthase dimers and loss of inner-membrane cristae in mitochondria. *Proc Natl Acad Sci USA* 110: 15301 – 15306

D'Aurelio M, Vives-Bauza C, Davidson MM, Manfredi G (2010) Mitochondrial DNA background modifies the bioenergetics of NARP/MILS ATP6 mutant cells. *Hum Mol Genet* 19: 374 – 386

Daussin FN, Heyman E, Burelle Y (2021) Effects of (−)-epicatechin on mitochondria. *Nutr Rev* 79: 25 – 41

de Oliveira MR, Nabavi SM, Braidy N, Setzer WN, Ahmed T, Nabavi SF (2016) Quercetin and the mitochondria: a mechanistic view. *Biotechnol Adv* 34: 532 – 549

Divakaruni AS, Paradyse A, Ferrick DA, Murphy AN, Jastroch M (2014) Analysis and interpretation of microplate-based oxygen consumption and pH data. *Methods Enzymol* 547: 309 – 354

Dubinin MV, Starinets VS, Talanov EY, Mikheeva IB, Belosludtseva NV, Serov DA, Tenkov KS, Belosludtseva EV, Belosludtsev KN (2021) Effect of the non-immunosuppressive MPT pore inhibitor alisporivir on the functioning of heart mitochondria in dystrophin-deficient mdx mice. *Biomedicine* 9: 1232

Ferguson SJ (2010) ATP synthase: from sequence to ring size to the P/O ratio. *Proc Natl Acad Sci USA* 107: 16755 – 16756

Fernandez-del-Rio L, Beninca C, Villalobos F, Shu C, Stiles L, Liesa M, Divakaruni AS, Acin-Perez R, Shirihai OS (2023) A novel approach to measure complex VATP hydrolysis in frozen cell lysates and tissue homogenates. *Life Sci Alliance*

Fernandez-Vizarra E, Zeviani M (2021) Mitochondrial disorders of the OXPHOS system. *FEBS Lett* 595: 1062 – 1106

Fernandez-Vizarra E, Bugiani M, Goffrini P, Carrara F, Farina L, Procopio E, Donati A, Uziel G, Ferrero I, Zeviani M (2007) Impaired complex III assembly associated with BCS1L gene mutations in isolated mitochondrial encephalopathy. *Hum Mol Genet* 16: 1241 – 1252

Filimonov DA, Lagunin AA, Gloriozova TA, Rudik AV, Druzhilovskii DS, Pogodin PV, Poroikov VV (2014) Prediction of the biological activity spectra of organic compounds using the pass online web resource. *Chem Heterocycl Comp* 50: 444 – 457

Formentini L, Pereira MP, Sanchez-Cenizo L, Santacatterina F, Lucas JJ, Navarro C, Martinez-Serrano A, Cuezva JM (2014) *In vivo* inhibition of the mitochondrial H^+-ATP synthase in neurons promotes metabolic preconditioning. *EMBO J* 33: 762 – 778

Garcia-Aguilar A, Cuezva JM (2018) A review of the inhibition of the mitochondrial ATP synthase by IF1 *in vivo*: reprogramming energy metabolism and inducing mitohormesis. *Front Physiol* 9: 1322

Garcia-Bermudez J, Cuezva JM (2016) The ATPase inhibitory factor 1 (IF1): a master regulator of energy metabolism and of cell survival. *Biochim Biophys Acta* 1857: 1167 – 1182

Gledhill JR, Montgomery MG, Leslie AG, Walker JE (2007) How the regulatory protein, IF_1, inhibits F_1-ATPase from bovine mitochondria. *Proc Natl Acad Sci USA* 104: 15671 – 15676

Grover GJ, Malm J (2008) Pharmacological profile of the selective mitochondrial F1F0 ATP hydrolase inhibitor BMS-199264 in myocardial ischemia. *Cardiovasc Ther* 26: 287 – 296

Grover GJ, Atwal KS, Sleph PG, Wang FL, Monshizadegan H, Monticello T, Green DW (2004) Excessive ATP hydrolysis in ischemic myocardium by mitochondrial F_1F_0-ATPase: effect of selective pharmacological inhibition of mitochondrial ATPase hydrolase activity. *Am J Physiol Heart Circ Physiol* 287: H1747 –H 1755

Hamer PW, McGeachie JM, Davies MJ, Grounds MD (2002) Evans Blue dye as an *in vivo* marker of myofibre damage: optimising parameters for detecting initial myofibre membrane permeability. *J Anat* 200: 69 – 79

Hammers DW, Hart CC, Matheny MK, Wright LA, Armellini M, Barton ER, Sweeney HL (2020) The D2.mdx mouse as a preclinical model of the skeletal muscle pathology associated with Duchenne muscular dystrophy. *Sci Rep* 10: 14070

Hochachka PW, Mommsen TP (1983) Protons and anaerobiosis. *Science* 219: 1391 – 1397

Husain I, Harris DA (1983) ATP synthesis and hydrolysis in submitochondrial particles subjected to an acid-base transition. Effects of the ATPase inhibitor protein. *FEBS Lett* 160: 110 – 114

Ivanes F, Faccenda D, Gatliff J, Ahmed AA, Cocco S, Cheng CH, Allan E, Russell C, Duchen MR, Campanella M (2014) The compound BTB06584 is an IF1-dependent selective inhibitor of the mitochondrial F1 Fo-ATPase. *Br J Pharmacol* 171: 4193 – 4206

Jiko C, Davies KM, Shinzawa-Itoh K, Tani K, Maeda S, Mills DJ, Tsukihara T, Fujiyoshi Y, Kuhlbrandt W, Gerle C (2015) Bovine F1Fo ATP synthase monomers bend the lipid bilayer in 2D membrane crystals. *Elife* 4: e06119

Johnson TA, Jinnah HA, Kamatani N (2019) Shortage of cellular ATP as a cause of diseases and strategies to enhance ATP. *Front Pharmacol* 10: 98

Kelly-Worden M, Thomas E (2014) Mitochondrial dysfunction in Duchenne muscular dystrophy. *Open J Endocr Metab Dis* 2014: 211 – 218

Khairallah RJ, Shi G, Sbrana F, Prosser BL, Borroto C, Mazaitis MJ, Hoffman EP, Mahurkar A, Sachs F, Sun Y *et al* (2012) Microtubules underlie dysfunction in duchenne muscular dystrophy. *Sci Signal* 5: ra56

Land H, Humble MS (2018) YASARA: a tool to obtain structural guidance in biocatalytic investigations. *Methods Mol Biol* 1685: 43 – 67

Lang DR, Racker E (1974) Effects of quercetin and F1 inhibitor on mitochondrial ATPase and energy-linked reactions in submitochondrial particles. *Biochim Biophys Acta* 333: 180 – 186

Law ML, Cohen H, Martin AA, Angulski ABB, Metzger JM (2020) Dysregulation of calcium handling in Duchenne muscular dystrophy-associated dilated cardiomyopathy: mechanisms and experimental therapeutic strategies. *J Clin Med* 9: 520

Liu S, Liu S, He B, Li L, Li L, Wang J, Cai T, Chen S, Jiang H (2021) OXPHOS deficiency activates global adaptation pathways to maintain mitochondrial membrane potential. *EMBO Rep* 22: e51606

Mamchaoui K, Trollet C, Bigot A, Negroni E, Chaouch S, Wolff A, Kandalla PK, Marie S, Di Santo J, St Guily JL *et al* (2011) Immortalized pathological human myoblasts: towards a universal tool for the study of neuromuscular disorders. *Skelet Muscle* 1: 34

Mareedu S, Million ED, Duan D, Babu GJ (2021) Abnormal calcium handling in Duchenne muscular dystrophy: mechanisms and potential therapies. *Front Physiol* 12: 647010

Martin-Jimenez R, Faccenda D, Allen E, Reichel HB, Arcos L, Ferraina C, Strobbe D, Russell C, Campanella M (2018) Reduction of the ATPase inhibitory factor 1 (IF1) leads to visual impairment in vertebrates. *Cell Death Dis* 9: 669

McGreevy JW, Hakim CH, McIntosh MA, Duan D (2015) Animal models of Duchenne muscular dystrophy: from basic mechanisms to gene therapy. *Dis Model Mech* 8: 195 – 213

Moore TM, Lin AJ, Strumwasser AR, Cory K, Whitney K, Ho T, Ho T, Lee JL, Rucker DH, Nguyen CQ *et al* (2020) Mitochondrial dysfunction is an early consequence of partial or complete dystrophin loss in mdx mice. *Front Physiol* 11: 690

Nesci S, Trombetti F, Ventrella V, Pagliarani A (2015) Opposite rotation directions in the synthesis and hydrolysis of ATP by the ATP synthase: hints from a subunit asymmetry. *J Membr Biol* 248: 163 – 169

Nicholls DG, Ferguson SJ, Publishing E, NetLibrary I (2013) *Bioenergetics 4*. Amsterdam: Elsevier, Academic Press

Osto C, Benador IY, Ngo J, Liesa M, Stiles L, Acin-Perez R, Shirihai OS (2020) Measuring mitochondrial respiration in previously frozen biological samples. *Curr Protoc Cell Biol* 89: e116

Ostojic J, Panozzo C, Lasserre JP, Nouet C, Courtin F, Blancard C, di Rago JP, Dujardin G (2013) The energetic state of mitochondria modulates complex III biogenesis through the ATP-dependent activity of Bcs1. *Cell Metab* 18: 567 – 577

Pettersen EF, Goddard TD, Huang CC, Couch GS, Greenblatt DM, Meng EC, Ferrin TE (2004) UCSF Chimera–a visualization system for exploratory research and analysis. *J Comput Chem* 25: 1605 – 1612

Ramos SV, Hughes MC, Delfinis LJ, Bellissimo CA, Perry CGR (2020) Mitochondrial bioenergetic dysfunction in the D2.mdx model of Duchenne muscular dystrophy is associated with microtubule disorganization in skeletal muscle. *PLoS One* 15: e0237138

Rego AC, Vesce S, Nicholls DG (2001) The mechanism of mitochondrial membrane potential retention following release of cytochrome c in apoptotic GT1-7 neural cells. *Cell Death Differ* 8: 995 – 1003

 © 2023 The Authors

Rieger B, Arroum T, Borowski MT, Villalta J, Busch KB (2021) Mitochondrial F1 FO ATP synthase determines the local proton motive force at cristae rims. *EMBO Rep* 22: e52727

Robergs RA, Ghiasvand F, Parker D (2004) Biochemistry of exercise-induced metabolic acidosis. *Am J Physiol Regul Integr Comp Physiol* 287: R502 – R516

Rouslin W, Broge CW, Grupp IL (1990) ATP depletion and mitochondrial functional loss during ischemia in slow and fast heart-rate hearts. *Am J Physiol* 259: H1759 –H 1766

Salewskij K, Rieger B, Hager F, Arroum T, Duwe P, Villalta J, Colgiati S, Richter CP, Psathaki OE, Enriquez JA *et al* (2020) The spatio-temporal organization of mitochondrial F1FO ATP synthase in cristae depends on its activity mode. *Biochim Biophys Acta Bioenerg* 1861: 148091

Schnizer R, Van Heeke G, Amaturo D, Schuster SM (1996) Histidine-49 is necessary for the pH-dependent transition between active and inactive states of the bovine F1-ATPase inhibitor protein. *Biochim Biophys Acta* 1292: 241 – 248

Sharma N, Medikayala S, Defour A, Rayavarapu S, Brown KJ, Hathout Y, Jaiswal JK (2012) Use of quantitative membrane proteomics identifies a novel role of mitochondria in healing injured muscles. *J Biol Chem* 287: 30455 – 30467

Sicinski P, Geng Y, Ryder-Cook AS, Barnard EA, Darlison MG, Barnard PJ (1989) The molecular basis of muscular dystrophy in the mdx mouse: a point mutation. *Science* 244: 1578 – 1580

Sorokina M, Merseburger P, Rajan K, Yirik MA, Steinbeck C (2021) COCONUT online: collection of open natural products database. *J Chem* 13: 2

St-Pierre J, Brand MD, Boutilier RG (2000) Mitochondria as ATP consumers: cellular treason in anoxia. *Proc Natl Acad Sci USA* 97: 8670 – 8674

Trott O, Olson AJ (2010) AutoDock Vina: improving the speed and accuracy of docking with a new scoring function, efficient optimization, and multithreading. *J Comput Chem* 31: 455 – 461

Urbani A, Giorgio V, Carrer A, Franchin C, Arrigoni G, Jiko C, Abe K, Maeda S, Shinzawa-Itoh K, Bogers JFM *et al* (2019) Purified F-ATP synthase forms a Ca^{2+}-dependent high-conductance channel matching the mitochondrial permeability transition pore. *Nat Commun* 10: 4341

Vesce S, Jekabsons MB, Johnson-Cadwell LI, Nicholls DG (2005) Acute glutathione depletion restricts mitochondrial ATP export in cerebellar granule neurons. *J Biol Chem* 280: 38720 – 38728

Vila MC, Rayavarapu S, Hogarth MW, Van der Meulen JH, Horn A, Defour A, Si T, Brown KJ, Hathout Y, Nagaraju K *et al* (2017) Mitochondria mediate cell membrane repair and contribute to Duchenne muscular dystrophy. *Cell Death Differ* 24: 330 – 342

Viscomi C, Zeviani M (2020) Strategies for fighting mitochondrial diseases. *J Intern Med* 287: 665 – 684

Wang L, Yuan L, Zeng X, Peng J, Ni Y, Er JC, Xu W, Agrawalla BK, Su D, Kim B *et al* (2016) A multisite-binding switchable fluorescent probe for monitoring mitochondrial ATP level fluctuation in live cells. *Angew Chem Int Ed Engl* 55: 1773 – 1776

Weissert V, Rieger B, Morris S, Arroum T, Psathaki OE, Zobel T, Perkins G, Busch KB (2021) Inhibition of the mitochondrial ATPase function by IF1 changes the spatiotemporal organization of ATP synthase. *Biochim Biophys Acta* 1862: 148322

Wolf DM, Segawa M, Kondadi AK, Anand R, Bailey ST, Reichert AS, van der Bliek AM, Shackelford DB, Liesa M, Shirihai OS (2019) Individual cristae within the same mitochondrion display different membrane potentials and are functionally independent. *EMBO J* 38: e101056

Yamazaki KG, Romero-Perez D, Barraza-Hidalgo M, Cruz M, Rivas M, Cortez-Gomez B, Ceballos G, Villarreal F (2008) Short- and long-term effects of (−)-epicatechin on myocardial ischemia-reperfusion injury. *Am J Physiol Heart Circ Physiol* 295: H761 –H 767

Yamazaki KG, Taub PR, Barraza-Hidalgo M, Rivas MM, Zambon AC, Ceballos G, Villarreal FJ (2010) Effects of (−)-epicatechin on myocardial infarct size and left ventricular remodeling after permanent coronary occlusion. *J Am Coll Cardiol* 55: 2869 – 2876

Yamazaki KG, Andreyev AY, Ortiz-Vilchis P, Petrosyan S, Divakaruni AS, Wiley SE, De La Fuente C, Perkins G, Ceballos G, Villarreal F *et al* (2014) Intravenous (−)-epicatechin reduces myocardial ischemic injury by protecting mitochondrial function. *Int J Cardiol* 175: 297 – 306

Yucel N, Chang AC, Day JW, Rosenthal N, Blau HM (2018) Humanizing the mdx mouse model of DMD: the long and the short of it. *NPJ Regen Med* 3: 4

Zabłocka B, Górecki DC, Zabłocki K (2021) Disrupted calcium homeostasis in Duchenne muscular dystrophy: a common mechanism behind diverse consequences. *Int J Mol Sci* 22: 11040

License: This is an open access article under the terms of the Creative Commons Attribution License, which permits use, distribution and reproduction in any medium, provided the original work is properly cited.

Expanded View Figures

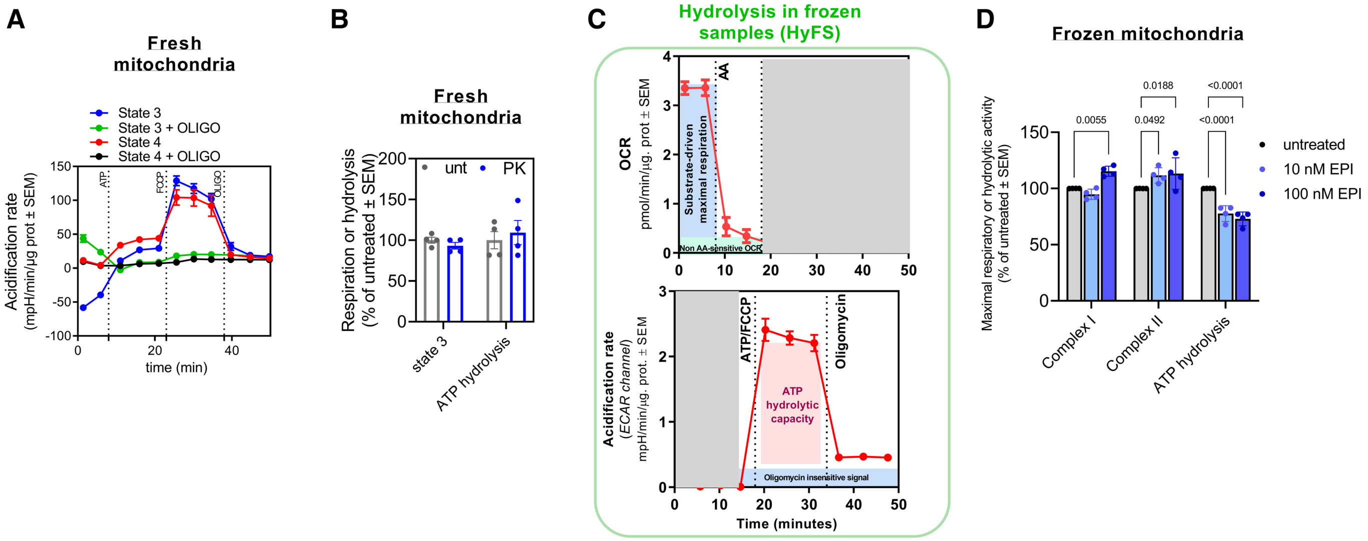

Figure EV1. ATP hydrolysis can be measured in fresh and frozen mitochondria (linked to main Fig 1). Also, see Appendix Supplementary Methods.

A Representative acidification rate traces of heart mitochondria respiring with the indicated substrates either in State 4 (no ADP) or State 3 (plus ADP) fueled by Pyruvate + Malate.

B State 3 and ATP hydrolytic activity in fresh heart mitochondria untreated or treated with proteinase K (PK) (n = 4).

C Overview of the ATP hydrolysis assay (HyFS). Representative trace of OCR (top) of a sample measured with the substrates of interest. Representative acidification rate (ECAR channel) trace from the same assay (bottom).

D Effects of increasing concentrations of EPI in maximal CI, CII and ATP hydrolytic activity measured in frozen mouse heart mitochondria (n = 4).

Data information: Each point represents a biological sample replicate. For each biological replicate, technical replicates were averaged. Data represent average ± SEM. Two-way ANOVA followed by Šídák's multiple comparisons test shows statistical differences depicted by *P*-value.

 © 2023 The Authors

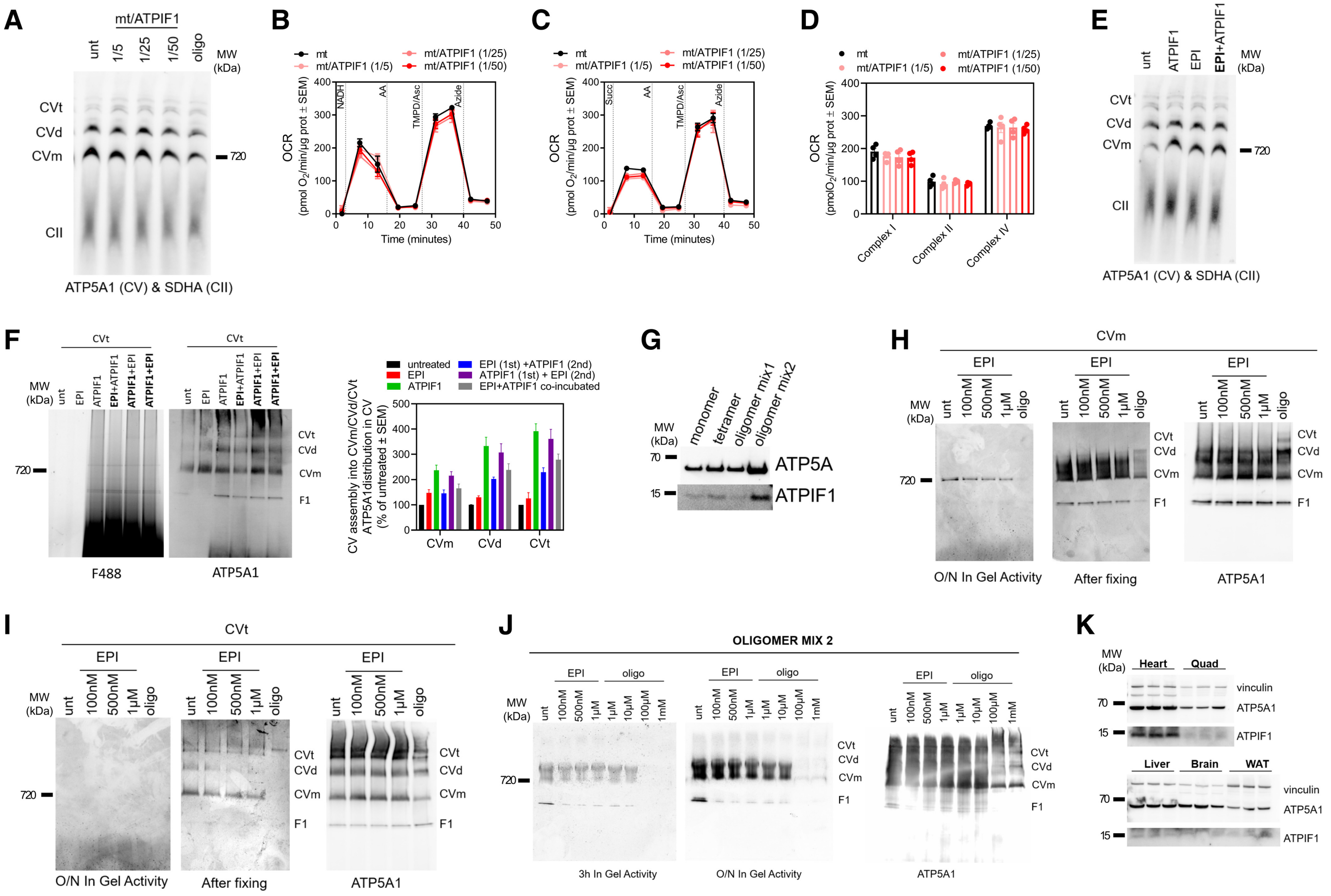

Figure EV2. EPI binds to ATPIF1 pocket in CV (linked to main Fig 3).

A CV assembly in heart mitochondria incubated in the presence of different concentrations of ATPIF1-GFP. CII (SDHA) was used as loading control.

B–D Representative Seahorse traces showing the effects of increasing concentrations of ATPIF1-GFP added to mitochondria on maximal CI, CII and CIV (B, C) activity measured in frozen mouse heart mitochondria and its quantification (D) (n = 4).

E CV assembly in heart mitochondria incubated with ATPIF1, EPI or both. CII (SDHA) was used as loading control.

F Representative blots showing the competition assay between ATPIF1-GFP and EPI for binding to CV tetramer (CVt) (left) and their quantification (right).

G Expression levels of ATP5A1 and ATPIF1 in the indicated bovine CV preparations.

H–J CV in gel ATP hydrolytic activity from purified bovine CV preparations under the indicated EPI and oligo concentrations; CV monomer (H) CV tetramer (I), and oligomer mix 2 (J). CVm: CV monomer; CVt: CV tetramer. CV in gel activity is measured after O/N incubation or 3h (J) (left blot) and after stopping the activity with 50% methanol (middle blot). Western blot for ATP5A1 was used as loading control (right blot).

K Expression levels of ATP5A1 and ATPIF1 in mouse tissue lysates. Vinculin is used as loading control.

Data information: For each biological replicate, technical replicates were averaged. Data represent average ± SEM.

© 2023 The Authors

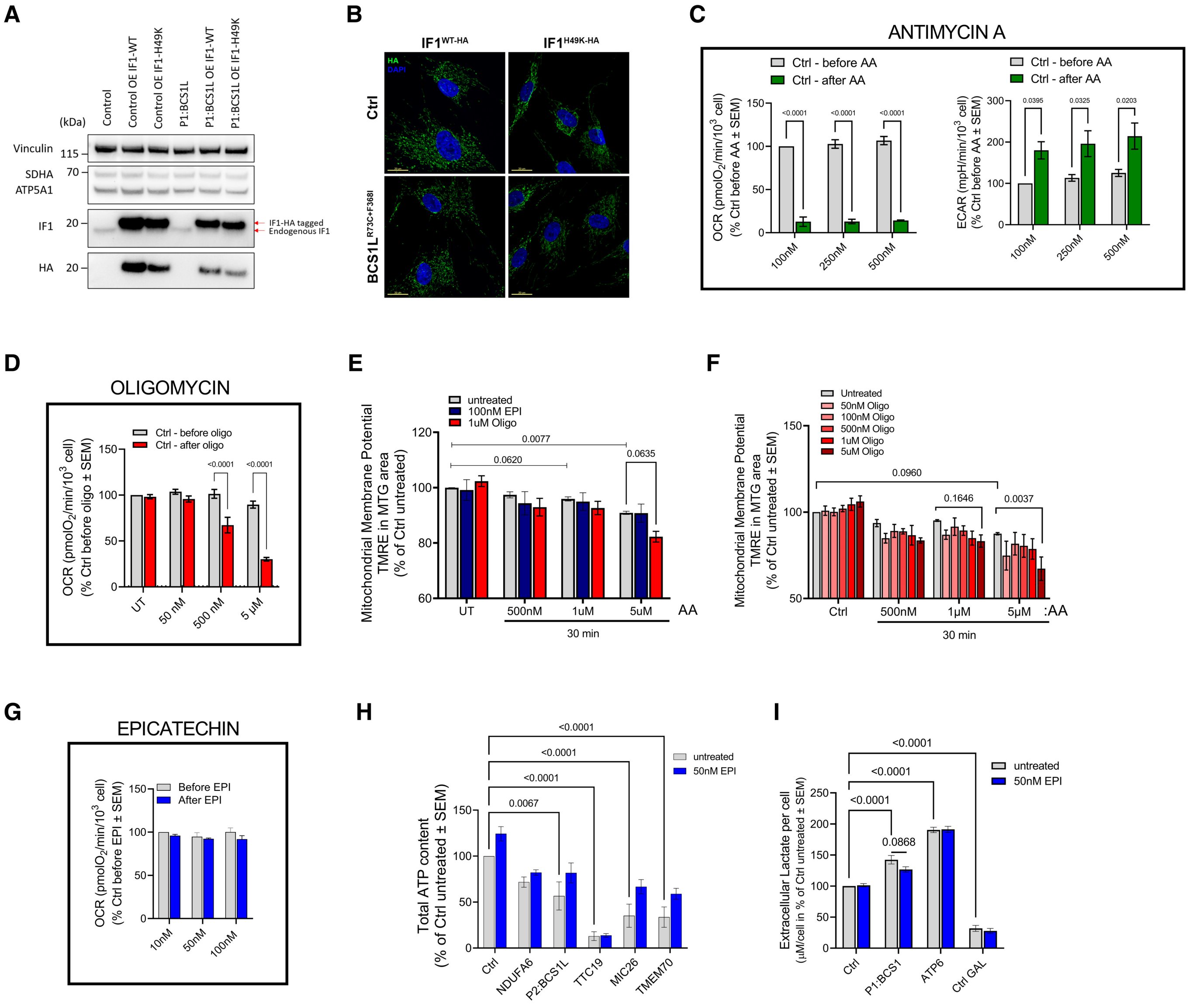

Figure EV3. Screening of EPI effect and inhibition of ATP hydrolysis in mitochondrial disease models (linked to main Fig 4).

A Representative SDS-PAGE blots for ATPIF1, HA, SDHB, ATP5A, and Vinculin showing the overexpression of ATPIF1-WT and H49K in Control (Ctrl) and CIII-deficient cells (P1:BCS1L).

B Representative confocal images showing mitochondrial localization of overexpressed ATPIF1-WT and H49K in Ctrl and CIII-deficient fibroblasts, labeled with anti-HA (green) antibody and DAPI (nuclei – blue). Maximum intensity projection is shown. Scale bars: 20 μm.

C Basal respiration (left) and acidification rates (right) in control fibroblasts before and after the direct injection of the indicated concentrations of Antimycin A (AA) (n = 4).

D Basal respiration in control fibroblasts before and after the direct injection of the indicated concentrations of oligomycin.

E, F Membrane potential measured using TMRE (average intensity) in Mitotracker Green (mitochondria area) normalized per % of untreated control: (E) control fibroblasts incubated for 30 min with AA plus DMSO, 100 nM EPI or 1 μM Oligo (n = 3); and (F) control fibroblasts incubated for 30 min with AA plus increased concentrations of Oligomycin (Oligo) (n = 3).

G Basal respiration (OCR) in control fibroblasts before and after the direct injection of the indicated concentrations of EPI (n = 4).

H Total ATP content measured in fibroblasts from patients with different mutations in mitochondrial proteins by luciferase assay and normalized by % of luminescence from control cells (n = 3).

I Extracellular lactate levels measured as % of the untreated control from Control (Ctrl), CIII-(P1:BCS1L) and CV-deficient (ATP6) fibroblasts treated with and without 50 nM EPI (n = 3). Control cells grown in galactose media (GAL) were included as a positive control for low levels of lactate.

Data information: In all cases, data represent average ± SEM. Two-way ANOVA followed by Šídák's multiple comparisons test shows statistical differences depicted by P-value.

 © 2023 The Authors

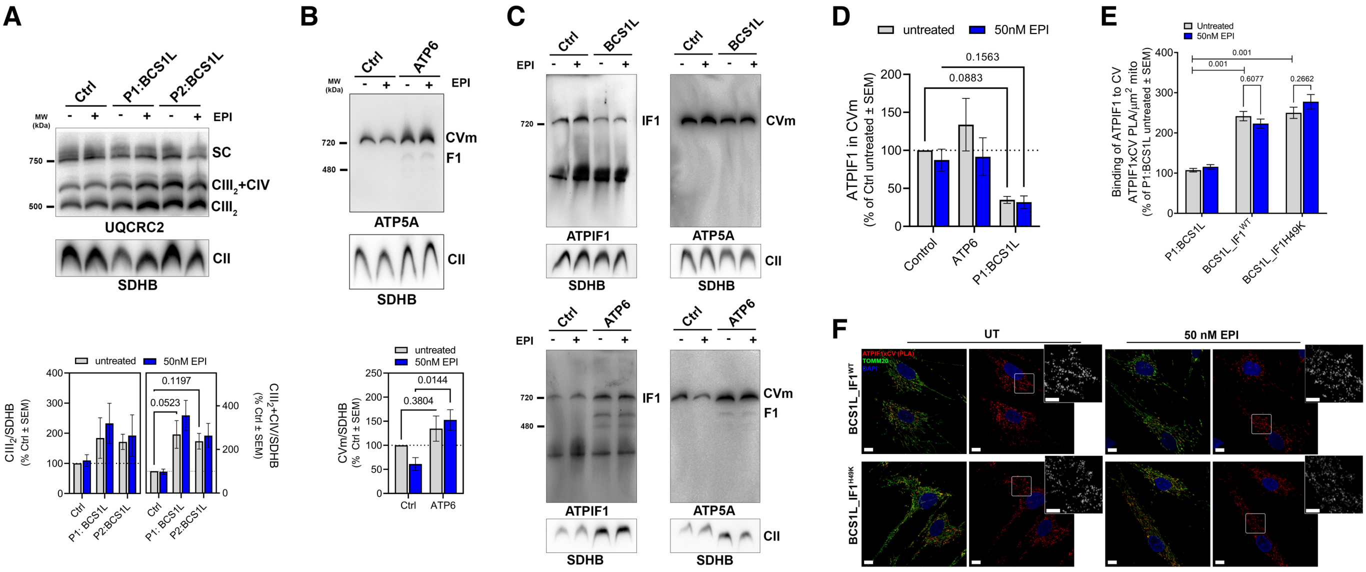

Figure EV4. EPI replaces ATPIF1 to block ATP hydrolysis in mitochondrial disease models (linked to main Fig 5).

A Representative BNGE blots (top) and quantification (bottom) of $CIII_2$, $CIII_2$ + CIV showing the distribution of CIII complexes and supercomplexes in control and CIII-deficient cells treated with and without 50 nM EPI for 24 h. Samples were immunoblotted with UQCRC2 antibody. SDHB was used as a loading control.

B Representative BNGE blots (top) and quantification (bottom) of CV monomer (CVm) supercomplex in control and CV-deficient cells treated with and without 50 nM EPI for 24 h. Samples were immunoblotted with ATP5A antibody. SDHB was used as a loading control.

C, D (C) Representative BNGE blots and quantification (D) of ATPIF1 relative intensity normalized per amount of CV and represented as % of control untreated cells in fibroblasts ($n = 3$). SDHB was used as a loading control.

E Chart shows PLA dots/μm^3 of mitochondria in fibroblasts normalized as % of Ctrl values ($n = 2$).

F Representative confocal images showing fibroblasts treated with EPI for 24 h, labeled with anti-ATPIF1 and anti-ATP5A1 (PLA in red) and anti-TOMM20 (green) antibodies. Maximum intensity projection is shown. Scale bars: 20 and 5 µm.

Data information: In all cases, data represent average ± SEM. Two-way ANOVA followed by Šídák's multiple comparisons test shows statistical differences depicted by *P*-value.

© 2023 The Authors

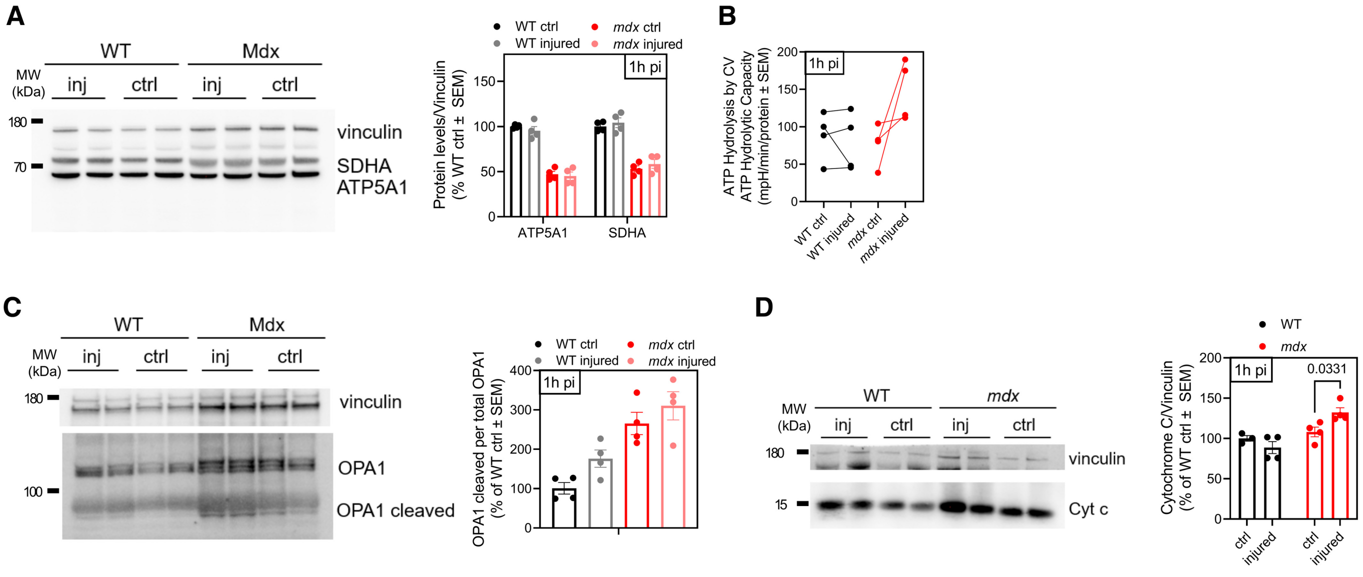

Figure EV5. ATP hydrolysis is increased in *mdx* eccentric injury model 1 h postinjury (linked to main Fig 6).

- A Representative western blot (right) and quantification (left) showing CV (ATP5A1) and CII (SDHA) levels in gastrocnemius homogenate in WT and *mdx* mice 1 h after eccentric injury. Vinculin was used as loading control ($n = 4$).
- B Maximal ATP hydrolysis capacity per total CV measured in frozen gastrocnemius homogenate in WT and *mdx* mice after 1 h of eccentric injury.
- C Analysis of OPA1 levels and isoforms (right) and quantification (left) in gastrocnemius homogenate in WT and *mdx* mice after 1 h of eccentric injury.
- D Cytochrome c release in gastrocnemius supernatants of WT and *mdx* 1 h postinjury measured by western blot. From (A–D), $n = 4$.

Data information: For each biological replicate, technical replicates were averaged. Data represent average ± SEM. Two-way ANOVA followed by Šídák's multiple comparisons test shows statistical differences depicted by *P*-value.

© 2023 The Authors

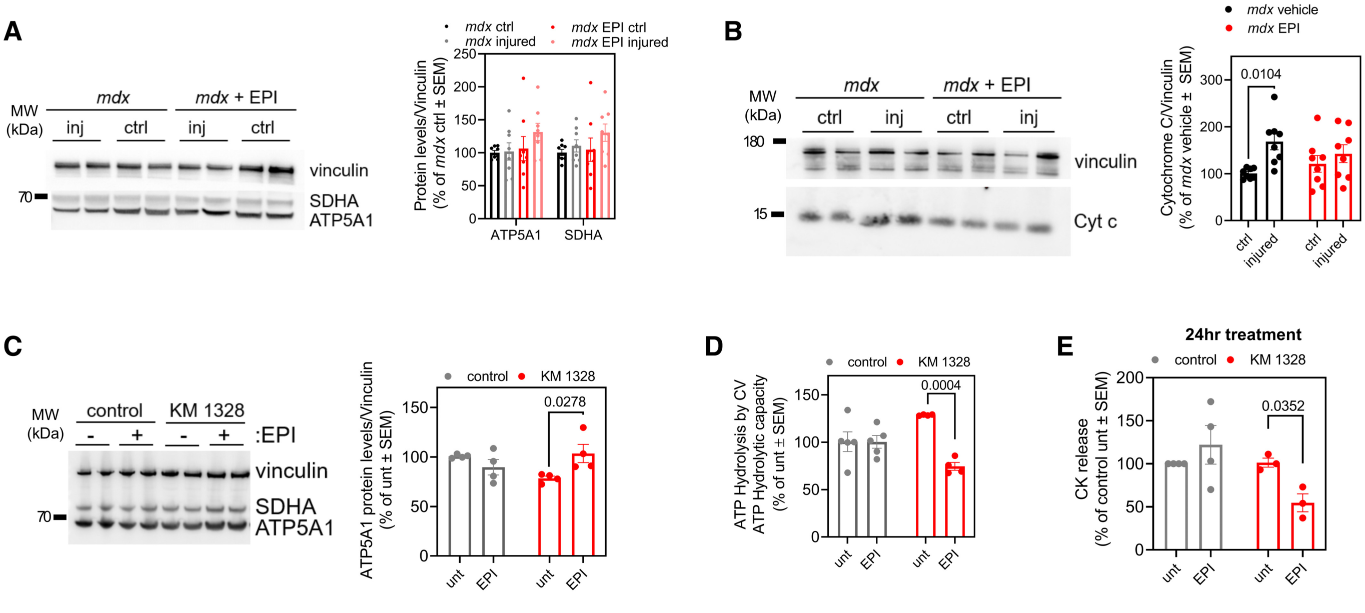

Figure EV6. *In vivo* and *in vitro* ATP hydrolysis inhibition by EPI in *mdx* mice and DMD cell lines (linked to main Fig 7).

A Representative western blot showing CV (ATP5A1) and CII (SDHA) levels in gastrocnemius homogenate in *mdx* vehicle or EPI treated 24 h after eccentric injury (left). Quantification of the protein levels (right). Vinculin was used as loading control (n = 8).

B Cytochrome c release in gastrocnemius supernatants of *mdx* vehicle or EPI treated 24 h after eccentric injury measured by western blot (n = 8). Quantification of the protein levels (right).Vinculin was used as loading control (n = 8).

C Representative western blot showing CV (ATP5A1) and CII (SDHA) levels in DMD cell lines after 24 h treatment with 50 nM EPI (left). Quantification of the protein levels versus vinculin versus control untreated (right). Vinculin was used as loading control ($n \geq 3$).

D Maximal ATP hydrolytic capacity in myotubes normalized by CV levels after 24 h of treatment with vehicle or 50 nM EPI ($n \geq 3$).

E Cell membrane stability as measured by creatine kinase (CK) release in myotubes treated 24 h with vehicle or 50 nM EPI ($n \geq 3$).

Data information: Each point represents a biological replicate. For each biological replicate, technical replicates were averaged. Data represent average ± SEM. Two-way ANOVA followed by Šídák's multiple comparisons test shows statistical differences depicted by P-value.

© 2023 The Authors

TABLE OF CONTENTS

Appendix Figures and Figure legends

Appendix Figure S1: Effect of Proteinase K in previously frozen samples on maximal respiration and maximal ATP hydrolytic capacity ……………………………………….....2

Appendix Figure S2: Epicatechin does not affect proton leak …..……………………….…3

Appendix Figure S3: Catechin does not inhibit CV ATP hydrolysis ………………………4

Appendix Figure S4: ATP hydrolysis inhibition by epicatechin in an oligomeric CV purified fraction from bovine mitochondria ………………………………………………………….5

Appendix Figure S5: Bioenergetics in mitochondrial deficient cells ………………………6

Appendix Figure S6: Mitochondrial content in gastrocnemius from *mdx* mice …………..7

Appendix Figures and Figure legends

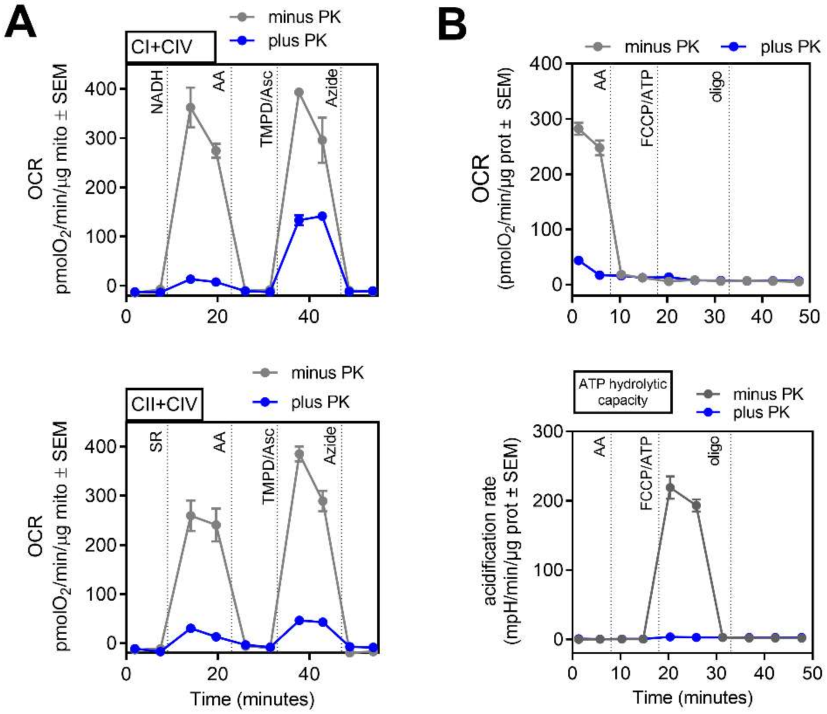

Appendix Figure S1: Effect of Proteinase K in previously frozen samples on maximal respiration and maximal ATP hydrolytic capacity

(**A**) Representative profile of RIFS respirometry in untreated or PK treated frozen mitochondria. CI and CIV (top panel) and CII and CIV (bottom panel). (**B**) Representative profiles of HyFS in untreated or PK treated frozen mitochondria. OCR (top panel) and acidification rate (bottom panel).

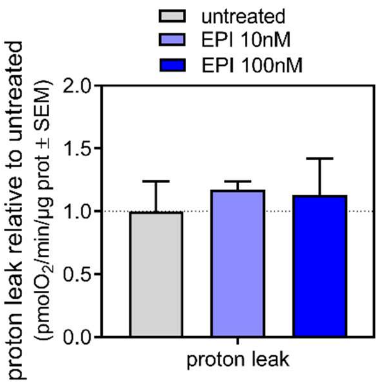

Appendix Figure S2: Epicatechin does not affect proton leak

Proton leak in isolated mitochondria from mouse heart. EPI was added in the respiration media at the indicated concentrations (n≥3). Note that EPI does not affect proton leak. For each biological replicate, technical replicates were averaged. Data represent average ± SEM.

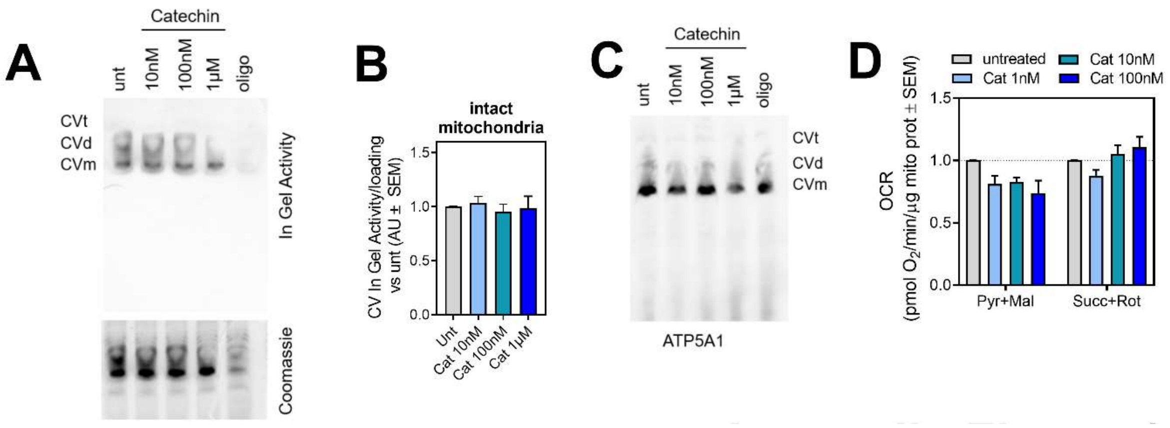

Appendix Figure S3: Catechin does not inhibit CV ATP hydrolysis.

(**A**) In gel ATP hydrolytic activity in mouse heart mitochondria incubated in the presence of the indicated concentrations of Catechin prior to freezing the mitochondrial preparation and lysing the mitochondria with digitonin. In gel activity is shown after 3hrs (top). Coomassie staining was used as loading control (bottom). Oligomycin (oligo) was used as control for ATP synthesis and hydrolysis inhibition. (**B**) Quantification of in gel ATP hydrolytic under the indicated Catechin concentrations (n=4). (**C)** Representative blot showing CV assembly by BNGE in mouse heart mitochondria incubated in the presence of the indicated concentrations of Catechin. (**D**) State 3 respiration driven by Pyr+Mal (left) and Succ+Rot (right) in isolated mitochondria from mouse heart. Catechin was added in the respiration media at the indicated concentrations (n=3). For each biological replicate, technical replicates were averaged. Data represent average ± SEM.

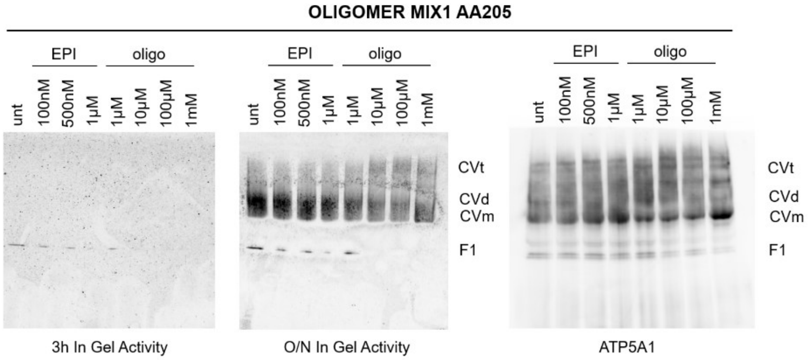

Appendix Figure S4: ATP hydrolysis inhibition by epicatechin in an oligomeric CV purified fraction from bovine mitochondria.

In gel ATP hydrolysis in oligomer mix 1 AA205 purified bovine CV preparation under the indicated EPI and oligo concentrations. In gel activity is shown after either 3hrs or O/N incubation (left) and after stopping the activity with 50% methanol (middle). Western blot for ATP5A1 was used as loading control (right).

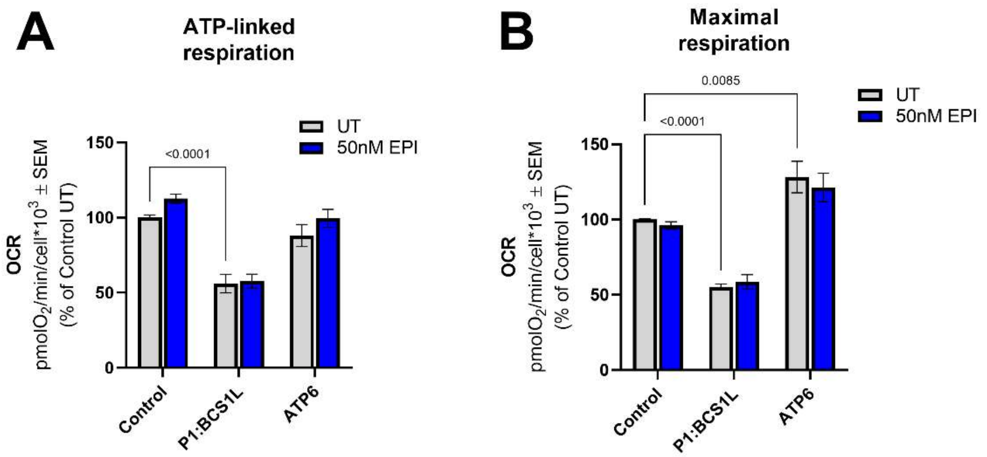

Appendix Figure S5: Bioenergetics in mitochondrial deficient cells

Bioenergetic analysis carried out in intact cells show that: **(A)** Compared to control cells, CIII-deficient fibroblasts (P1:BCS1L) have lower ATP-linked respiration, whereas CV-deficient fibroblast (ATP6) have similar levels. EPI does not influence ATP-linked respiration in any of the cell lines. **(B)** Compared to control cells, CIII-deficient cells exhibit a decrease in maximal respiration while CV-deficient cells reveal an increase in this parameter. EPI does not affect maximal respiration in any of the cell lines. Data represent average ± SEM of 8 independent biological replicates.

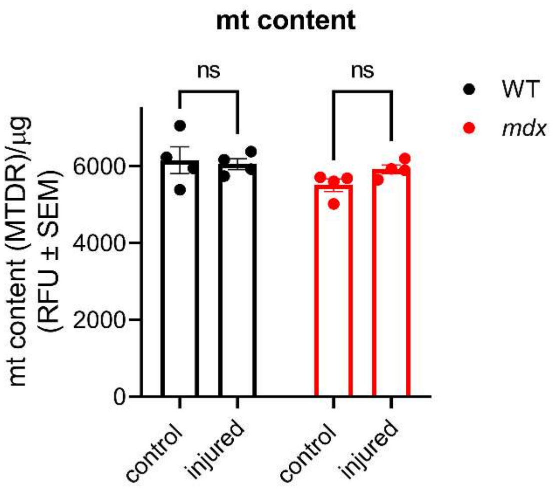

Appendix Figure S6: Mitochondrial content in gastrocnemius from *mdx* mice

Mitochondrial content is unchanged in *mdx* mice either in control or injured gastrocnemius.

Appendix S1: Expanded Experimental Information

2a. Rationale of using acidification as a read out for ATP hydrolysis measurement

Both the ATP synthase and hydrolase activities mitochondrial F_1F_O-ATP synthase can be indirectly measured in reductionist systems by monitoring pH changes in the experimental medium (Divakaruni *et al*, 2018; Sarkar & Chattopadhyay, 2017). Phosphorylation of ADP alkalinizes the experimental medium as it consumes H^+, and similarly, hydrolysis of ATP generates H^+ and acidifies the experimental medium. Specific involvement of the mitochondrial ATP synthase, distinct from other ATP hydrolyzing enzymes, is measured by acute sensitivity to low concentrations of oligomycin. The rough equation for ATP hydrolysis is given below, with the assumption that one of the two protons liberated during hydrolysis of two molecules of ATP will bind to inorganic phosphate (pKa=6.8).

$$2\ \text{Mg-ATP}^{2-} + 2\ H_2O \rightarrow 2\ \text{Mg-ADP}^{1-} + HPO_4^{2-} + H_2PO4^{1-} + H^+$$

When using the Seahorse XF Analyzer, quantitative values of ATP hydrolyzed by the ATP synthase can be obtained by calibrating the ECAR signal with known quantities of H^+ to obtain a H+ production rate (Desousa B.R. *et al*, 2022). Any reaction that results in the net change in free H^+ in the experimental medium can be detected with the pH channel of the XF Analyzer, be it the conversion of uncharged glucose to anionic lactate during glycolysis (glucose $\rightarrow$ 2 lactate^{-1} + 2H^+) or ATP hydrolysis in reductionist systems as detailed above (Divakaruni *et al.*, 2014). Use of the XF Analyzer to measure mitochondrial ATP hydrolysis was first conducted by McQuaker et al. (McQuaker *et al*, 2013).

2b. Studying ATP hydrolysis in previously frozen mitochondria

Frozen mitochondria (HyFS)

The term *previously frozen mitochondria* refer to mitochondria that have been frozen and thawed. In this process membrane sealing is breached to allow free diffusion. This procedure is used for respiratory analysis as we have previously shown (Acin-Perez *et al.*, 2020a). These mitochondria are completely uncoupled and do not maintain membrane potential. Previously frozen mitochondria cannot respire unless provided with direct electron donors, such as NADH or succinate to the electron transport chain complexes (Acin-Perez *et al.*, 2020a).

The analysis of ATP hydrolysis in previously frozen mitochondria is described in detail in (Fernandez-del-Rio L. *et al.*, in press)

2c. Studying ATP hydrolysis in intact mitochondria

Intact mitochondria are mitochondria isolated from cells or tissue, where the membrane integrity it preserved well enough to support respiration. Unless subjected to specific treatments, intact mitochondria pertain to mitochondria that are coupled, in which oxygen consumption is coupled to ATP synthesis. The term “coupled mitochondria” also means that the level of proton leak is maintained at physiological range for these cells or tissue.

The analysis of CV ATP hydrolysis in intact mitochondria presented additional challenges:

A. How do we assure we are looking at CV ATP hydrolysis and not at ATP hydrolysis by other hydrolyzes? Mitochondria contain a variety of ATP hydrolyses that are not CV.

   To determine the acidification rate produced by other hydrolyses, we inhibit CV by oligomycin. Oligomycin resulted in over 95% inhibition of the rate of acidification. This is in agreement with high abundancy of CV is in the mitochondria as compared to any other protein, including other hydrolyzes.

B. How we assure that acidification is not contributed by carbon dioxide product of respiration or by lactate produced in glycolysis?

   Coupled mitochondria can respire and produce CO_2 which forms acid. To assure CO_2 is not a significant source of protons we reduced respiration by shifting ATP/ADP ratio towards higher levels of ATP, thereby producing a stable state 4, in which respiration is inhibited by the product of ATP synthase, ATP. Normally, state 4 pertains to a sample depleted of ADP. Here we created a state 4 ATP, in which ATP concentrations are high enough to slow down respiration.

C. How we assure that acidification is not contributed by lactate produced in glycolysis?

   Lactate production through glycolysis requires supply of glucose and glycolytic enzymes, including lactate dehydrogenase. Isolated mitochondria preps do not have a supply of glucose and the cytosolic enzyme lactate dehydrogenase is removed during the purification of the mitochondria.

D. How we address the contribution of proton leak to hydrolysis?

   Proton leak is not sufficient to induce ATP hydrolysis. It is the proton motive force that has to reverse itself due to depolarization. Depolarization will stimulate respiration. Our results show that the addition of ATP did not result in an increase in respiration. To the contrary, it resulted in further inhibition of respiration.

REFERENCES

Acin-Perez R, Benador IY, Petcherski A, Veliova M, Benavides GA, Lagarrigue S, Caudal A, Vergnes L, Murphy AN, Karamanlidis G, Tian R, Reue K, Wanagat J, Sacks H, Amati F, Darley-Usmar VM, Liesa M, Divakaruni AS, Stiles L, Shirihai OS (2020) A novel approach to measure mitochondrial respiration in frozen biological samples. EMBO J 39: e104073

Desousa B.R., Kim K.K.O., Hsieh W.Y., Jones A.E., Swain P., Morrow D.H., Ferrick D.A., Shirihai O.S., Neilson A., Nathanson D.A., Rogers G.W., Dranka B.P., Murphy A.N., Affourtit C, Bensinger S.J., L. S, Romero N., A.S. D (2022) Calculating ATP production rates from oxidative phosphorylation and glycolysis during cell activation.

Divakaruni AS, Hsieh WY, Minarrieta L, Duong TN, Kim KKO, Desousa BR, Andreyev AY, Bowman CE, Caradonna K, Dranka BP, Ferrick DA, Liesa M, Stiles L, Rogers GW, Braas D, Ciaraldi TP, Wolfgang MJ, Sparwasser T, Berod L, Bensinger SJ et al. (2018) Etomoxir Inhibits Macrophage Polarization by Disrupting CoA Homeostasis. Cell Metab 28: 490-503 e7

Divakaruni AS, Paradyse A, Ferrick DA, Murphy AN, Jastroch M (2014) Analysis and interpretation of microplate-based oxygen consumption and pH data. Methods Enzymol 547: 309-54

Fernandez-del-Rio L., Benincá C., Villalobos F., Shu C., Stiles L., Liesa M., Divakaruni A.S., Acin-Perez R., O.S. S (in press) A Novel Approach to Measure Complex V ATP Hydrolysis in Frozen Cell Lysates and Tissue Homogenates. Life Science Alliance

McQuaker SJ, Quinlan CL, Caldwell ST, Brand MD, Hartley RC (2013) A prototypical small-molecule modulator uncouples mitochondria in response to endogenous hydrogen peroxide production. Chembiochem 14: 993-1000

Sarkar P, Chattopadhyay A (2017) Solubilization of the serotonin(1A) receptor monitored utilizing membrane dipole potential. Chem Phys Lipids 209: 54-60
