# Supplementary material for: Inhibition of ATP synthase reverse activity restores energy homeostasis in mitochondrial pathologies
Source: EMBO J. 2023 Mar 13;42(10):e111699. doi: 10.15252/embj.2022111699 (PMC10183817; doi:10.15252/embj.2022111699)
Supplement: Supplementary file 12 [file EMBJ-42-e111699-s005.pdf]

THE EMBO JOURNAL

Inhibition of ATP synthase reverse activity restores energy homeostasis in mitochondrial pathologies

Orian Shirihai, Rebeca Acín-Pérez, Cristiane Benincá, Lucia Fernandez del Rio, Cynthia Shu, Siyouneh Baghdasarian, Vanessa Zanette, Christoph Gerle, Chimari Jiko, Ramzi Khairallah, Shaharyar Khan, David Rincon Fernandez Pacheco, Byourak Shabane, Karel Erion, Ruchi Masand, Sundeep Dugar, Cristina Ghenoiu, George Schreiner, Linsey Stiles, and Marc Liesa **DOI: 10.15252/embj.2022111699**

Corresponding author(s): Orian Shirihai (oshirihai@mednet.ucla.edu) , Marc Liesa (mlrbmc@ibmb.csic.es)

Review Timeline:

Submission Date:	19th May 22
Editorial Decision:	21st Jun 22
Revision Received:	12th Oct 22
Editorial Decision:	8th Nov 22
Revision Received:	17th Dec 22
Editorial Decision:	11th Jan 23
Revision Received:	20th Jan 23
Editorial Decision:	24th Jan 23
Revision Received:	24th Jan 23
Accepted:	25th Jan 23

Editor: William Teale

Transaction Report:

(Note: With the exception of the correction of typographical or spelling errors that could be a source of ambiguity, letters and reports are not edited. Depending on transfer agreements, referee reports obtained elsewhere may or may not be included in this compilation. Referee reports are anonymous unless the Referee chooses to sign their reports.)

Dear Prof. Shirihai,

Thank you for submitting your manuscript for consideration by the EMBO Journal. It has now been seen by three referees whose comments are shown below.

At its heart, all referees agree that the work is based on a technically accomplished collection of experiments. They also state unambiguously that the manuscript is timely and the topic is highly important. However, the feedback was not unambiguously positive.

I would like to invite you to address the comments of all referees in a revised version of the manuscript. As you will see, two experts in the field struggled to extract the significance of your findings. I therefore urge you to re-organise the manuscript to focus the readers' attention on your novel experimental insights and the clinical perspectives they provide. I will be available and happy to talk over Zoom in two week's time if you have any questions, or would like to discuss the referees' comments further.

I should add that it is EMBO Journal policy to allow only a single round of revision, and acceptance of your manuscript will therefore depend on the completeness of your responses in this revised version.

When preparing your letter of response to the referees' comments, please bear in mind that this will form part of the Review Process File, and will therefore be available online to the community. For more details on our Transparent Editorial Process, please visit our website: https://www.embopress.org/page/journal/14602075/authorguide#transparentprocess

We generally allow three months as standard revision time, although this is a guideline rather than a deadline. As a matter of policy, competing manuscripts published during this period will not negatively impact on our assessment of the conceptual advance presented by your study. However, we request that you contact the editor as soon as possible upon publication of any related work, to discuss how to proceed. Should you foresee a problem in meeting this three-month deadline, please let us know in advance and we may be able to grant an extension.

Thank you for the opportunity to consider your work for publication. I look forward to your revision.

Yours sincerely,

William

William Teale, PhD
Editor
The EMBO Journal
w.teale@embojournal.org

When submitting your revised manuscript, please carefully review the instructions below and include the following items:

1) a .docx formatted version of the manuscript text (including legends for main figures, EV figures and tables). Please make sure that the changes are highlighted to be clearly visible.

2) individual production quality figure files as .eps, .tif, .jpg (one file per figure).

3) a .docx formatted letter INCLUDING the reviewers' reports and your detailed point-by-point response to their comments. As part of the EMBO Press transparent editorial process, the point-by-point response is part of the Review Process File (RPF), which will be published alongside your paper.

4) a complete author checklist, which you can download from our author guidelines (https://wol-prod-cdn.literatumonline.com/pb-assets/embo-site/Author Checklist%20-%20EMBO%20J-1561436015657.xlsx). Please insert information in the checklist that is also reflected in the manuscript. The completed author checklist will also be part of the RPF.

5) Please note that all corresponding authors are required to supply an ORCID ID for their name upon submission of a revised manuscript.

6) We require a 'Data Availability' section after the Materials and Methods. Before submitting your revision, primary datasets produced in this study need to be deposited in an appropriate public database, and the accession numbers and database listed under 'Data Availability'. Please remember to provide a reviewer password if the datasets are not yet public (see https://www.embopress.org/page/journal/14602075/authorguide#datadeposition). If no data deposition in external databases is needed for this paper, please then state in this section: This study includes no data deposited in external repositories. Note that

the Data Availability Section is restricted to new primary data that are part of this study.

Note - All links should resolve to a page where the data can be accessed.

7) When assembling figures, please refer to our figure preparation guideline in order to ensure proper formatting and readability in print as well as on screen:
http://bit.ly/EMBOPressFigurePreparationGuideline

Please remember: Digital image enhancement is acceptable practice, as long as it accurately represents the original data and conforms to community standards. If a figure has been subjected to significant electronic manipulation, this must be noted in the figure legend or in the 'Materials and Methods' section. The editors reserve the right to request original versions of figures and the original images that were used to assemble the figure.

8) For data quantification: please specify the name of the statistical test used to generate error bars and P values, the number (n) of independent experiments (specify technical or biological replicates) underlying each data point and the test used to calculate p-values in each figure legend. The figure legends should contain a basic description of n, P and the test applied. Graphs must include a description of the bars and the error bars (s.d., s.e.m.).

9) We would also encourage you to include the source data for figure panels that show essential data. Numerical data can be provided as individual .xls or .csv files (including a tab describing the data). For 'blots' or microscopy, uncropped images should be submitted (using a zip archive or a single pdf per main figure if multiple images need to be supplied for one panel). Additional information on source data and instruction on how to label the files are available at .

10) We replaced Supplementary Information with Expanded View (EV) Figures and Tables that are collapsible/expandable online (see examples in https://www.embopress.org/doi/10.15252/embj.201695874). A maximum of 5 EV Figures can be typeset. EV Figures should be cited as 'Figure EV1, Figure EV2" etc. in the text and their respective legends should be included in the main text after the legends of regular figures.

- For the figures that you do NOT wish to display as Expanded View figures, they should be bundled together with their legends in a single PDF file called *Appendix*, which should start with a short Table of Content. Appendix figures should be referred to in the main text as: "Appendix Figure S1, Appendix Figure S2" etc. See detailed instructions regarding expanded view here: .

- Additional Tables/Datasets should be labelled and referred to as Table EV1, Dataset EV1, etc. Legends have to be provided in a separate tab in case of .xls files. Alternatively, the legend can be supplied as a separate text file (README) and zipped together with the Table/Dataset file.

11) Our journal encourages inclusion of *data citations in the reference list* to directly cite datasets that were re-used and obtained from public databases. Data citations in the article text are distinct from normal bibliographical citations and should directly link to the database records from which the data can be accessed. In the main text, data citations are formatted as follows: "Data ref: Smith et al, 2001" or "Data ref: NCBI Sequence Read Archive PRJNA342805, 2017". In the Reference list, data citations must be labeled with "[DATASET]". A data reference must provide the database name, accession number/identifiers and a resolvable link to the landing page from which the data can be accessed at the end of the reference. Further instructions are available at .

Further instructions for preparing your revised manuscript:

Please make sure you upload a letter of response to the referees' comments together with the revised manuscript.

Please also check that the title and abstract of the manuscript are brief, yet explicit, even to non-specialists.

When assembling figures, please refer to our figure preparation guideline in order to ensure proper formatting and readability in print as well as on screen:
https://bit.ly/EMBOPressFigurePreparationGuideline
See also guidelines for figure legends: https://www.embopress.org/page/journal/14602075/authorguide#figureformat

IMPORTANT: When you send the revision we will require
- a point-by-point response to the referees' comments, with a detailed description of the changes made (as a word file).
- a word file of the manuscript text.
- individual production quality figure files (one file per figure)
- a complete author checklist, which you can download from our author guidelines (https://www.embopress.org/page/journal/14602075/authorguide).
- Expanded View files (replacing Supplementary Information)
Please see out instructions to authors

https://www.embopress.org/page/journal/14602075/authorguide#expandedview

Please remember: Digital image enhancement is acceptable practice, as long as it accurately represents the original data and conforms to community standards. If a figure has been subjected to significant electronic manipulation, this must be noted in the figure legend or in the 'Materials and Methods' section. The editors reserve the right to request original versions of figures and the original images that were used to assemble the figure.

Further information is available in our Guide For Authors: https://www.embopress.org/page/journal/14602075/authorguide

We realize that it is difficult to revise to a specific deadline. In the interest of protecting the conceptual advance provided by the work, we recommend a revision within 3 months (19th Sep 2022). Please discuss the revision progress ahead of this time with the editor if you require more time to complete the revisions. Use the link below to submit your revision:

https://emboj.msubmit.net/cgi-bin/main.plex

Referee #1:

This is an interesting paper which shows i) that oxidative phosphorylation is balanced by ATP hydrolysis within populations of mitochondria or within single cells, ii) that the balance may shift in favour of hydrolysis with disease and iii) that the compound epicatechin reduces hydrolysis by acting as an ATPIf1 mimetic, with potential benefit in some disease models.
I have to confess that I found the paper surprisingly hard to read and very hard to navigate. The overall message is certainly interesting, but the way it is presented is so overcomplicated that it is very easy to get confused and lost. The writing style is convoluted and laborious, the figures are very dense and hard to interpret and I feel strongly that the authors don't do their case justice. This is an excellent lab and I have no doubt that the quality of the work is excellent and the overall message is valuable and so I sincerely hope that the authors will accept my comments as supportive, as this is how they are intended. Please bear in mind that having accepted the role of reviewer I am obliged to work my way through the paper, but most readers won't have that investment and I'm afraid I feel that most won't bother.
Overall I urge the authors to simplify the language and the figures, trim down and remove material that is superfluous to make sure that the key message is transmitted with clarity. I appreciate the authors ambition to show all their data but most readers won't be interested in seeing every experiment, and so my advice would be to try to trim down as much as possible and move data to supplementary wherever possible to streamline the 'story'. In particular, Figures with so many tiny panels are almost impossible to read. I had to zoom onto some figures just to try to understand what they show or to read the axis labels. There is no question for me that for this paper, 'less will be more', in terms of content, language and illustration.
I will go through my thoughts about the manuscript in the order in which the work is presented.
1. Highlights are almost exactly the same as the abstract just split into statements. It would be good to shorten and simplify the highlights.
2. The description of the protocol is very laborious. The approach is elegant and quite simple but many readers will get lost. In Fig 1A, why does OCR decrease before ATP is added? What units are the changes in pH ? ('mpH units' but this looks like it has been normalised?) Again, the changes seem to precede drug additions, presumably because these have been misaligned?) This figure is key and needs to be crystal clear.
3. As a general point it really helps reviewing if you put page numbers on the text and label figures with a figure number.
4. I'm not sure why you need fig 1D or F here and would think these can go to supp figures?
5. Fig 1H you have plotted the WB values as ATPIF1 vs SDHB/vinculin (I'm not really sure what that means - how do measure against two proteins? - why not against ATP5A which is its binding partner?
6. These data do beg the question of what endogenous ATPIF1 is doing. Why does it not suppress ATP hydrolysis at rest? Have you done the same experiment with knockout of ATPIF1 to assess the role of the protein in suppressing ATP wastage at rest?
7. I note also that these experiments are done with mitochondria from mouse heart. Work going back to Rouslin suggested that ATPIF1 expression was very limited in small rodent heart. You haven't measured ATPIF1 levels in that prep as far as I can see - it would be very useful to know whether these data look very different if you use isolated mitochondria from bovine or even human heart (e.g.atrial appendage?)? I'd expect to see some reference to PMID: 1625346 somewhere here?
8. I don't understand what is shown in Fig 1L. what is being measured as 'activity' of complex I and II? This seems not to be explained at all.
9. In Fig 1D you use a red symbol for PK. This confuses interpretation of 1E, where red means something else. I didn't find the explanation of how you reach the numbers in Fig 1E very clear and this is very important as this is an interesting and important observation.
10. Fig 1I - 'epicathechin'?
11. Page 14; 'we analyzed ATP hydrolytic activity in tissues expressing different amounts of ATPIF1 (Figure S2H).' - where did data showing different levels of expression in different tissues come from? I am surprised there is no reference to PMID: 19559621
12. Fig 2- I'm not convinced that you really need to show all panels to 1A;

13. 2D and E could go to supplementary. Its not clear in the text what preparation was used for these measurements nor how were they made - are these intact cells or isolated mitochondria? Measured using Seahorse?
14. Effects of catechin (Fig 2F-I) are all negative - can these go to supp?
15. Page 13 and Fig 3: 'ATPIF1 inhibition' sounds like it means inhibition of the action of ATPIF1 but I think what you are doing is looking at inhibition of ATP hydrolysis by ATPIF1? I am surprised that you do not refer to PMID: 18590689 here?
16. Fig 3 explores the action of added recombinant ATPIF1 but we don't know how much endogenous protein there is? Or have I misunderstood? How do you know that endogenous levels are not already close to saturation?
17. Do you really need Fig 3L _?move to supp?
18. I think Fig 3M needs to be quantified?
19. Page 15 when introducing the H49K mutation worth stating that this is pH insensitive.
20. 'In cells expressing either ATPIF1-WT or H49K', should probably read 'overexpressing'?
21. Fig 4F I don't understand why EPI fails to cause depolarisation in the presence of AA if the membrane potential is being maintained by reversal of the synthase? If EPI selectively inhibits the reversed synthase activity you would expect potential to decrease? This is incorrectly referred to as Fig 4G in the text.
22. From Fig 4 G onwards this is referred to as the 'ATP fluorogenic dye' - what dye is this? Has it been validated? It is misleading to describe this as a more specific measure than TMRM - it is measuring something different - presumably. I have no idea Fig 4G mtATP measurements - no idea what is being measured or how this works and is really unconvincing. The arguments over whether ATP is imported through the reversed ANT is all confusing and adds unnecessary complication to an already complex paper. I don't understand why you don't simply use FCCP to drive maximal reversal of the synthase and promote hydrolysis?
23. I have concerns and reservations about all subsequent measures of 'mtATP' as it isn't clear what is being measured.
24. Fig 4K - the effects of EPI on lactate seem insignificant. A, I missing something?

25. Page 21- what do you mean in cells 'with endogenous levels of ATPIF1'? with high levels? Low levels?
26. P22 I guess you don't really mean 'no addictive effect of EPI'?!
27. I'm afraid that I don't feel qualified to comment on the mdm mouse experiments- my overall impression is that these are as convoluted and confusing as the rest of the paper. I am concerned that epicatechin has been shown to have multiple effects in cells and tissues and so interpreting changes in an animal model specifically in relation to effects on the ATP synthase may be difficult.

Overall I urge the authors to try to trim down this paper as much as they can at all levels to try to improve clarity and reduce 'noise'.

Referee #2:

Summary. The studies reported in this manuscript detail the affect of epicatechin on the activity of the ATP synthase. Specifically, the authors conclude that epicatechin inhibits ATP hydrolysis, but not ATP synthesis, of the ATP synthase by binding to the same site as the natural inhibitor protein, IF1. Furthermore, the studies suggest that epicatechin can help in the treatment of diseases that affect the electron transport chain and the ATP synthase, including Duchene Muscular dystrophy. In this context, epicatechin, is shown to increase the ATP content in the mitochondria and improve muscle force without an increase in mitochondria content.

Review: Overall, this is certainly an important study and may provide a new drug that may modulate the disease phenotype of subset of mitochondrial diseases. A problem I had was that the number of experiments presented was extremely dense. As a reviewer, I felt obliged to assess each figure, but as a reader, I would not have. There were some studies that I thought were ancillary and would be better omitted. Further, there are issues I had with the interpretation of the results. The authors certainly have a strong background in oxidative phosphorylation, and yet I struggled with understanding some of the studies. I must admit that I have never used a Seahorse XF96, which might add to some of my confusion, but at least on the surface, I understand the workings of the Seahorse.

With that stated, I will spend some time on some of my difficulties. (I am a little disappointed that there are no page numbers or line numbers, which makes referencing that point more difficult.)

The results start with talking about protocol to measure ATP hydrolysis and ATP synthesis at the same time.

The statement is made that 2 protons are net transported with the hydrolysis of ATP and that is referenced. But we know that there are 8 c-subunits in the ATP synthase, so that number is 2.67. But maybe the term "net" transport is important. With the hydrolysis at ATP at pH 7.0, there are 3/4 H+ released, due to the formation of Pi and ADP. In addition, there is an electrogenic exchange of ATP for matrix ATP, when measuring ATP hydrolysis. There are also some other electrogenic pump, including the

Pi transporter that brings in H+ into the matrix. But these issues become important later. Lastly, it should not be ignored that the respiratory chain pumps out protons.

In Figure 1, there are an oxygen consumption studies that shows state 3 and 4 respiration. First, it would have been good it the P/O ratio was reported. In Fig. A top, there are points for state 3 and state 4 respiration. There are 2 points before ATP is added. For State 3 control, the rate of respiration is steadily decreasing and may have continued, but ATP was added before the rate stabilize. It is not clear what the addition of ATP did, if anything. But, state 3 respiration requires the addition of ADP, and by adding ATP, that might remove ADP from the matrix, basically shifting it to state 4 with ATP. Then FCCP is added and respiration jumps for state 3 but not much for state 4. It is still difficult to understand why there is a difference in state 3 vs state 4 if ATP was added and if ADP is seemingly lost from the matrix.

In Fig. 1A bottom, it looks at the acidification of the buffer. As before, there are 2 time points before ATP addition, and it is unclear what ATP addition did. The addition of FCCP caused a sharp rise in acidification. The reason for this is as stated earlier: when ATP is hydrolyzed, it releases 3/4 H+. The reason they give for this increase in ATP hydrolysis relates to the conclusion they are trying to press that in mitochondria, there are complexes that are active in ATP hydrolysis and those in synthesis. While this may be correct, the increase in the rate of ATP hydrolysis here is that the system is uncoupled and ATP hydrolysis is favored over synthesis and the protons feely flow from the matrix to the buffer solution.

There are also some studies using Proteanase K that as used to show that the mitochondria were intact. But we are more concerned about the coupling, and the mitochondria can be impermeable to proteinase K, but not protons.

I had some issue with their use of "frozen" mitochondria. I think it would be better if they referred as "previously frozen" and detail whether what as frozen - the tissue or the mito. At the end of the para, they state "Addition of oligomycin at different concentrations in the assay media using frozen mitochondria revealed a dose dependent inhibition of ATP hydrolysis (Figure 1F), demonstrating the specificity of the assay." I'm not sure what they were saying other than there are not any other ATPases present.

Before the next section of in silico screening, they state: "We identified down regulation of ATPIF1 together with increasing ATP hydrolytic capacity as a common adaptive mechanism in mitochondrial diseases. This mechanism would allow for cytosolic ATP consumption to maintain membrane potential and consequently prevent eventual apoptotic cell death (Chen, Birsoy et al. 2014)." But this statement is in complete conflict with the conclusions in this study. In this study, they suggest that inhibition of the ATPase activity is better for the cell. But the statement above, suggests that opposite.

The Fig. 1I would be better if the interaction of Epicatechin with the F1 was shown. It also would have been good if the inhibition assay was done using isolated F1. In 1K, it is shown that Epicatechin only inhibits ATPase activity by 30% even at 0.1mM (Fig. 1L). If it is acting like IF1, then that must mean that it is displaced from the binding site - that the affinity is not high. This comes in later.

The experiments in Fig. 2 were done to reveal more basis for the interaction of Epicatechin with the ATP synthase. I would have liked to see more direct studies on this question. In Fig. 2A, the EPI is added to the mito, the enzymes extracted in detergent, and then run on a BN gel. The ATPase activity was measure in the gel, with 3 hr incubation. The inhibition of activity is shown to be much dramatic than 30% - this is despite the fact that the enzyme complex has been run through a BN gel without EPI. This is totally unexpected and suggests that something more has happened. Further, the 3hr time frame hard to interpret as for comparison, it is not clear how linear activity is over that time period.

For Fig. 2E, they state: "Changes in state 3 but not in MRR confirm that EPI is targeting ATP synthase." But this is not quite correct. It is possible that EPI altered ATP/ADP transport or maybe Pi transport. Maybe more.

For Fig. 2J-K, the question being addressed seems to be pretty simple. I say this because the figure adds to the work the reader has to do.

The remaining experiments are slight variations of the initial experiment and don't seem to provide much information - at least relative to the amount of effort is takes to go over them.

For Fig. 3, they state: To compare the two mechanisms of action, we performed similar experiments where we block ATPIF1 activity with the specific inhibitor BTB06584 (BTB)." But the paper they reference indicates that BTB "is an IF1-dependent selective inhibitor of the" ATP synthase. This is just the opposite of what is presented in this paper.

I also don't understand the use of IF1-GFP. Can this get into the mitochondria? If so, it wasn't shown. But I would not expect it to get in as the import of proteins in the mito is done during translation, and not post translation. Also, the GFP is folded, and even if it did get to the import machinery, it would not likely unfold to allow import.

But even so, I don't see much value to the experiments in Fig. 3 as BTB is not the main discovery in this paper. I suggest that this figure is dropped.

For Fig 4, the amount of information is small for the amount of effort it takes to go over the figures. There should be a control for the effect of EPI on PLA.

In this set of experiments, they use the IF1 mutant, H49K - which they describe as "constitutively active". This is correct, as it has to do with the protonation of His which is essential for binding to F1. This has been the basic reason why IF1 has been proposed to be an inhibitor of ATPase but not synthesis since it cannot bind to the ATP synthase at pH 8.0, the pH during state 3 respiration. The authors contend that IF1 inhibits ATPase and hydrolysis, while this is not the prevailing view. The papers they cite are not entirely consistent with this idea. This is pretty central to the mechanism they are proposing.

In Fig. 4F, G, it is stated: " Adding 1uM oligomycin further depolarized mitochondria, demonstrating that under AA, the mitochondrial ATP synthase was working in reverse. No changes were observed when EPI was incubated with AA, supporting the hypothesis that EPI will only act in the molecules of ATP synthase working in reverse and where ATPIF1 could be displaced (Figure 4G)." I think this is using data from Fig, 4F and 4G, but the assays are different - potential vs ATP content. So, I'm not sure the conclusion can made.

In Fig. 4H, the basis for the importance of this m/s, the level of ATP is measure with a fluorescent dye. I would have preferred direct measurement, but the odd result is that the effect is not present at 1uM EPI but was observed at 50nM even though the effect on ATPase is much better at 1uM than at 50nM, see Fig. 2B, for instance.

Fig. 4J is very confusing as it is not possible to ID the points.

At one point they say: "With these results, we propose that mitochondrial ATP hydrolysis is a common response to electron transport dysfunction." I think this idea is not unique and widely accepted as the potential gradient is essential for cell survival.

In the text, they say "...amelioration of the disease phenotype in CIII-BCS1L patient cells." I think this is a bit strong.

In the text they say "... suggesting that ATPIF1 displacement can only be detected if sufficient ATPIF1 is expressed and bound to ATP synthase.." I'm not sure what the point is here.

I think the data in Fig. 6 and 7, is the most important of the paper. But it took a long time to get to this point and I, was totally exhausted by the time I got to these figures. And, it is not entirely clear how much of the data shows a significant difference between treated and not treated.

This is stated a number of times "mitochondrial ATP Fluorescence Intensity". This is inaccurate. The exact dye used should be stated and maybe some reference as to how well it works.

I would also suggest being careful in using abbreviations. The writers assume that the reader recognizes the abbreviations. Please be careful to define them.

I think it also important to indicate what the "n" means in each experiment. What do each "n" represent? Do they represent another assay with the same preparation or another assay with a second preparation?

Overall, I would suggest making this paper more easily read. Do not include figures that are not to the point. Try to reduce the amount of data. This paper tries to do 2 things - show the mechanism of EPI and show that it helps in mitochondrial diseases. Maybe this is too much.

Referee #3:

The mitochondrial F1FO ATP synthase, working in the reverse mode as an ATPase, preserves the mitochondrial membrane potential under conditions of low ATP. The authors of the study reasoned that an increase in mitochondrial ATP hydrolysis, rather than decreased synthesis, can be detrimental in mitochondrial disease and contribute to pathogenesis. Using isolated mitochondria and monitoring oligomycin-sensitive pH changes, the authors assessed ATP hydrolysis in parallel to ATP synthesis and OXPHOS activities. They show that in cultured cells about 20% of the ATP synthase works in the reverse mode and that this fraction increases in patient-derived fibroblast with OXPHOS deficiencies. An in silico screen identified (+) Epicathechin (EPI) as a modulator of the F1FO ATP synthase. Experiments using isolated mitochondria and cellular models indicate that EPI acts as a competitive inhibitor of ATPIF1. In contrast to ATPIF1, EPI selectively inhibits the ATPase but not the ATP synthase activity. Further experiments reveal that low dose EPI treatment restores total ATP content in patient fibroblasts and show beneficial effects of EPI on muscular performance in Mdx mice in vivo.
The demonstration that ATP synthase hydrolyzes ATP in respiring mitochondria is intriguing and supports the emerging view that the proton gradient across the mitochondrial inner membrane is heterogenous. Similarly, it is a novel and interesting

concept to selectively inhibit ATP hydrolysis in mitochondrial OXPHOS disease. The beneficial effects of EPI treatment on muscle performance in Mdx mice underlines the potential of such an approach. At the same time, however, some of the conclusions of the authors remains unclear or appear premature and many issues need to be clarified.
1. The authors provide evidence that EPI acts as a competitive inhibitor of ATPIF1. Modelling suggests binding to the same binding site, but why EPI only affects ATPase while ATPIF1 inhibits both ATPase and synthase remains unclear. Some of the descriptions of the EPI effects are rather confusing (´dependence on ATPIF1`?). The experiments shown in Fig. 3MN are not convincing and need quantification.
2. The authors should provide additional evidence that the detected pH changes reflect exclusively ATP hydrolysis by the F1FO ATPase. The authors argue that isolated mitochondria are used, but it remains unclear how other (oligomycin-induced) effects within mitochondria can be excluded (for instance, proton-coupled channels etc.)
3. The analysis of possible beneficial effects of EPI treatment under conditions of low ATP or in patient fibroblast is very limited. The authors show the expected increase in ATP levels (Fig 4) but how this affects cell proliferation or other metabolic parameters typically associated with OXPHOS deficiencies is not analyzed (even though stated on p. 17). This would be important to substantiate the idea of the authors that such an approach could be a novel treatment strategy in mitochondrial disease.
4. Related to this point, I have a conceptual concern: Increased ATP hydrolysis by F1FO is thought to maintain the mitochondrial membrane potential in OXPHOS deficiency or under low ATP conditions. It therefore appears difficult to envision that inhibition of the ATPase could have significant beneficial effects.
5. The authors use different concentrations of EPI, ranging from 1 nM to 10 µM. The rationale remains unclear (for instance, 50 or 100 nM were used in some experiments in fibroblasts). Dose dependence is only shown for ATP hydrolysis, but not for ATP synthesis or respiration, precluding a direct comparison. Information is missing about how much EPI used in Fig. 2J, 3M, 4A, 5A and was administrated to mice in the experiments in Fig. 7.
6. The manuscript contains many inconsistencies, typos, wrong figure callouts and is rather difficult to read. Some statements appear not justified. For instance, the authors state on p23L16 that EPI administration twice a day for 2 weeks was able to decrease susceptibility to injury in EPI treated mdx mice in a dose-dependent manner compared to vehicle (Figure 7A-C). However, the figures do not show such a dose-dependent effect. On p17 in the caption of the section, it is stated in the caption that EPI treatment increases cell growth, but I could not find any data showing cell growth in Fig. 4 or S3. There are many errors in figure callouts. P16 last line, Figure 4F not 4G. P21, it seems that the order of figures (from 5G to 5I) is wrong in the figure. Legend to Fig. S3E is apparently missing.

The overall impression by all the reviewers is that the manuscript is very dense and convoluted, containing a lot of experimental models and data. In this revised version of the manuscript, we have simplified the text, removed the data deemed unnecessary by the reviewers and moved results to Extended View Figures as well as to the On-line Appendix. In the case of control data validating our new protocol to measure hydrolysis, we have now moved all these data to a new manuscript describing the ATP hydrolysis method (under review in LSA, LSA-2022-01628).

Please find below the point-by-point response to the reviewers.

Referee #1:

This is an interesting paper which shows i) that oxidative phosphorylation is balanced by ATP hydrolysis within populations of mitochondria or within single cells, ii) that the balance may shift in favour of hydrolysis with disease and iii) that the compound epicatechin reduces hydrolysis by acting as an ATPIf1 mimetic, with potential benefit in some disease models.
I have to confess that I found the paper surprisingly hard to read and very hard to navigate. The overall message is certainly interesting, but the way it is presented is so overcomplicated that it is very easy to get confused and lost. The writing style is convoluted and laborious, the figures are very dense and hard to interpret and I feel strongly that the authors don't do their case justice. This is an excellent lab and I have no doubt that the quality of the work is excellent and the overall message is valuable and so I sincerely hope that the authors will accept my comments as supportive, as this is how they are intended. Please bear in mind that having accepted the role of reviewer I am obliged to work my way through the paper, but most readers won't have that investment and I'm afraid I feel that most won't bother. Overall I urge the authors to simplify the language and the figures, trim down and remove material that is superfluous to make sure that the key message is transmitted with clarity. I appreciate the authors ambition to show all their data but most readers won't be interested in seeing every experiment, and so my advice would be to try to trim down as much as possible and move data to supplementary wherever possible to streamline the 'story'. In particular, Figures with so many tiny panels are almost impossible to read. I had to zoom onto some figures just to try to understand what they show or to read the axis labels. There is no question for me that for this paper, 'less will be more', in terms of content, language and illustration.

I will go through my thoughts about the manuscript in the order in which the work is presented.

1. Highlights are almost exactly the same as the abstract just split into statements. It would be good to shorten and simplify the highlights.

We thank the reviewer for his/her comment. We have shortened and simplified the highlights:

- A new protocol to quantify ATP hydrolysis by mitochondrial ATP synthase (CV) in fresh mitochondria is described.
- ATP hydrolysis by CV can occur in coupled mitochondria
- Epicatechin is a competitive and partial mimetic of ATPIF1, as it selectively inhibits ATP hydrolysis without inhibiting ATP synthesis.
- Selective inhibition of CV-ATP hydrolysis prevents ATP depletion independent of respiratory dysfunction.

2. The description of the protocol is very laborious. The approach is elegant and quite simple but many readers will get lost.

We appreciate this comment deeming that our approach is elegant. We have simplified the description of the protocol by dividing the material and methods text in two independent sections, based on the type of samples analyzed. The title of the new sections are:

1) Respirometry in isolated mitochondria and homogenates
2) ATP hydrolysis in previously frozen samples (HyFS)

We have also an additional section to explain how we calculate the proportions of ATP synthesis vs ATP hydrolysis under the heading: Determination of ATP synthetic versus hydrolytic capacity in intact mitochondria.

In addition, we provide now a graphical abstract of the protocol in Figure 1A, clarifying how ATP synthesis and hydrolysis are detected in intact mitochondria, and how FCCP (chemical uncoupling) and freeze- thawing (physical uncoupling) causes all mitochondrial ATP synthase molecules to act in reverse and hydrolyze. The scheme also shows that oligomycin will block both ATP synthesis and hydrolysis, revealing that oligomycin treatments are needed to confirm to specificity of acidification rates measuring complex V hydrolytic activity. In Figure 1B, by covering part of the traces with a grey panel, we highlight when ATP synthesis is being measured, by quantifying OCR prior to ATP injection (blue traces are wells with ADP, state 3). We also highlight when ATP hydrolysis is being measured, by quantifying acidification rates after ATP injection in mitochondria in the red trace (wells without ADP, state 4).

Furthermore, we submitted a separated manuscript that describes the protocol in extensive detail and includes all the laborious controls to validate these new methods. The generation of this second manuscript allow an extensive simplification of this manuscript. This separated manuscript is currently under review in LSA (LSA-2022-01628)

In Fig 1A, why does OCR decrease before ATP is added?

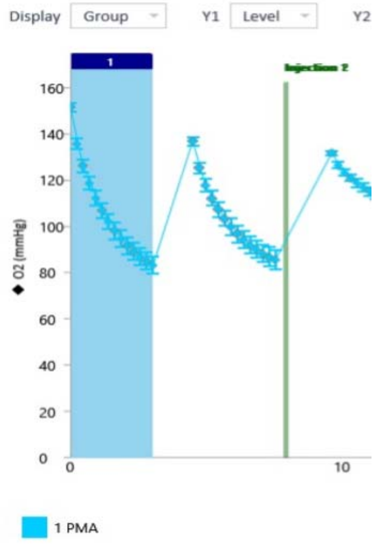

Figure 1. Excessive mitochondrial activity leads to a decrease in oxygen tension (O2), which cannot be recovered by mixing after the first measurement, explaining a decrease in OCR in the second measurement

Before the ATP is added, mitochondria are coupled and respiring under high concentrations of ADP and fuels (state 3, blue trace, now new Figure 1B). When the concentration of substrates required for maximal ATP synthesis is not saturated and substrates become limiting, a decline over time in OCR will be observed. As millimolar concentrations of ADP and oxidative fuels are present in our assays, the most common cause for such a decrease in OCR is when a mitochondrial preparation has an unexpectedly high activity that consumes too much oxygen. The decline in oxygen tension in the media can be sufficiently large that the standard mixing time between measurements will not be able to return to the initial values (see Figure 1 for the reviewer). Consequently, the following OCR measurement will start with less oxygen available, resulting in an effective decrease in OCR (less substrate available). In this letter, we provide the absolute values of the oxygen concentrations of the graph in previous Fig 1A (now Figure 1B), demonstrating that the excessive decline in oxygen tension explains the decrease in the second OCR measurement before the ATP is added (Figure 1 for the reviewer). Further confirming that the decrease in OCR is not explained by mitochondria being damaged during the assay, these same mitochondria markedly increase

respiration in response to FCCP. Such an increase can only occur if the mitochondria remain coupled and functional.

What units are the changes in pH? ('mpH units' but this looks like it has been normalised?) Again, the changes seem to precede drug additions, presumably because these have been misaligned?) This figure is key and needs to be crystal clear.

We thank the reviewer for giving us the opportunity to clarify this issue. Indeed, pH is an absolute unit that cannot be normalized. However, the change in mpH units over time, namely acidification rates, are exquisitely sensitive to the amount of mitochondria loaded. Therefore, we are normalizing the acidification rates, mpH units/minute per the total amount of protein loaded. We are not normalizing the raw mpH units. This has been clarified now in the y axis.

The graphs have not been misaligned. Changes in the rates of media acidification will be concurrent to changes in ATP synthesizing respiration. The reason is that mitochondrial ATP synthesis consumes protons, which basifies the media where mitochondria are respiring. High levels of mitochondrial ATP synthesis, and thus of proton consumption, explain the negative values of the acidification rates in the first two measurements under state 3. In the second measurement before ATP injection, the acidification rates become less negative because there is a decrease in ATP synthesizing respiration due to limited oxygen availability (see previous answer and Figure 1 for the reviewer). Indeed, the change in acidification rates perfectly matches the changes in OCR. Therefore, the fact that the changes in acidification rates match the changes in coupled OCR is strong evidence confirming the specificity of the assay measuring mitochondrial ATP synthesis. To make this figure crystal clear, we are now showing the quantification of OCR and acidification rates in the new Figure 1C and D, simplified the traces in Figure 1B and moved the complete traces to Supplementary (Figure EV1A). Furthermore, we provide a new scheme in Fig 1A that clarifies the relationship between acidification rates and complex V activity under the different respiratory states, as well as in intact versus previously frozen samples.

3. As a general point it really helps reviewing if you put page numbers on the text and label figures with a figure number.

We acknowledge the reviewer for his/her observation. We have now included page, line and figure numbers in the revised version of the manuscript.

4. I'm not sure why you need fig 1D or F here and would think these can go to supp figures?

This is a great suggestion. We have moved Figure 1D to Supplementary Figure EV1B. We have completely removed Figure 1F and the right panel of Figure 1J, since they do not provide essential evidence to support the main conclusion of this paper. However, these removed figure panels provide relevant information validating the specificity of the protocol and as a consequence they have been transferred to the methodology manuscript under review in LSA, LSA-2022-01628.

5. Fig 1H you have plotted the WB values as ATPIF1 vs SDHB/vinculin (I'm not really sure what that means - how do measure against two proteins? - why not against ATP5A which is its binding partner?

We have now plotted ATP1F1 against ATP5A1 in Figure 1E, as suggested by the reviewer. The normalization to ATP5A1 provides an even larger decrease in ATPIF1 expression in most cell lines.

6. These data do beg the question of what endogenous ATPIF1 is doing. Why does it not suppress ATP hydrolysis at rest? Have you done the same experiment with knockout of ATPIF1 to assess the role of the protein in suppressing ATP wastage at rest?

We thank the reviewer for bringing up this interesting point. ATPIF1 KO cells are not commercially available and KO approaches can cause long-term adaptations to change gene expression and remodel mitochondria. Therefore, to determine the acute role of ATPIF1 in ATP hydrolysis, we silenced ATPIF1 transfecting 2 different siRNA sequences and measured ATPIF1 protein content and ATP hydrolysis 48 hours after transfection (Figure 2 for the reviewers).

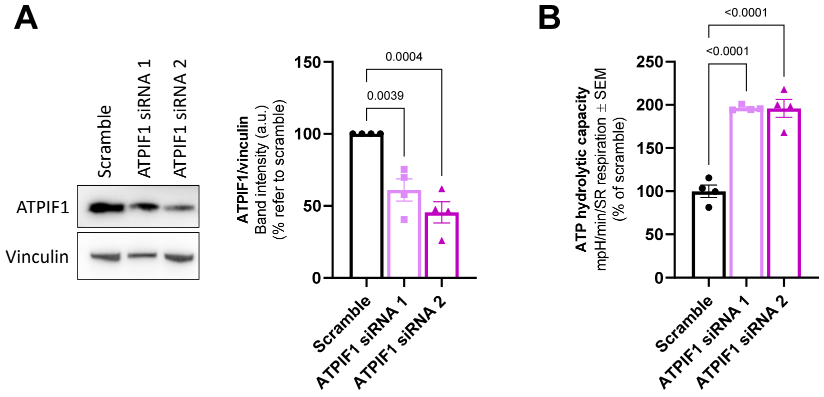

Figure 2. Knockdown of ATPIF1 increases ATP hydrolysis. **A**. Western blots analyses measuring ATPIF1 expression in HEK293T transfected with two siRNA against ATPIF1. **B**. ATP hydrolytic capacity measured in in HEK293T transfected with two siRNA against ATPIF1.

The expression of 2 different siRNA against ATPIF1 induced a 50% decrease in ATPIF1 protein content and a doubled total ATP hydrolytic capacity (Figure 2 for the reviewers). These results are consistent with the decrease in ATP hydrolytic capacity observed in control (Figure 4E) and CIII-deficient (Figure 5E) fibroblasts overexpressing the ATPIF1 mutant (H49K), which cannot detach from complex V.

Given that both the reviewers and editors recommended to simplify the manuscript, we have included the ATPIF1 in the methods paper submitted to LSA, as a biologically relevant model that confirms the validity of the method.

7. I note also that these experiments are done with mitochondria from mouse heart. Work going back to Rouslin suggested that ATPIF1 expression was very limited in small rodent heart. You haven't measured ATPIF1 levels in that prep as far as I can see - it would be very useful to know whether these data look very different if you use isolated mitochondria from bovine or even human heart (e.g.atrial appendage?)? I'd expect to see some reference to PMID: 1625346 somewhere here?

We measured ATPIF1 expression by Western blot in tissue lysates, including heart from mouse. The results were included in the original submission in Figure S2H (now Figure EV2K) and showed that IF1 expression was higher in heart than quadriceps. Given that ATPIF1 protein is confined to mitochondria, we expect that its expression profile in isolated mitochondria will be very similar to the profile in tissue lysates (now Figure EV2K). We believe that addressing the discrepancies on the amount of ATPIF1 detected in mouse, human, and bovine heart is beyond the scope of our manuscript. Addressing those will not change our main conclusion that ATP hydrolysis can occur in coupled mitochondria.

We thank the reviewer for his/her suggestion to discuss reference PMID 1625346. We initially omitted this reference because only showed ATP measurements in the cytosol in intact cardiomyocytes. We will be happy to discuss this reference if the reviewer believes the changes in cytosolic ATP caused by hypoxia can add to the message of our paper, without making the manuscript and discussion more convoluted.

8. I don't understand what is shown in Fig 1L. what is being measured as 'activity' of complex I and II? This seems not to be explained at all.

We thank the reviewer for pointing the lack of a description in the activity measurements in former Figure 1L (now Figure EV1D) y axis and its legend. This activity is oxygen consumption fueled by NADH, which provides electrons directly to complex I, and by succinate, which delivers electrons directly to complex II. The ATP hydrolysis condition was measured by adding ATP alone. The protocol used for this "activity" measurement is Respirometry In Frozen Samples (RIFS), which we previously published (PMID 32432379). These experiments aimed to validate whether the changes in mitochondrial function were a consequence of EPI selectively targeting complex V and other components of the electron transport chain. Now, we clarified that the activity is maximal oxygen consumption for complex I and II and acidification for ATP hydrolysis. To simplify Figure 1, we moved this panel to supplementary (now Figure EV1D).

9. In Fig 1D you use a red symbol for PK. This confuses interpretation of 1E, where red means something else. I didn't find the explanation of how you reach the numbers in Fig 1E very clear and this is very important as this is an interesting and important observation.

We have now moved former Figure 1D to Figure EV1B and changed the color of PK to blue, which will prevent the confusion with the red color. We thank the reviewer for pointing that the figure panel and legend were not clear and were missing important information. We have clarified in the text and methods how we have reached the number referring to the proportion of ATP synthetizing vs ATP hydrolyzing coupled mitochondria (now in the methods under the heading: Determination of ATP synthetic versus hydrolytic capacity in intact mitochondria).

10. Fig 1I - 'epicathechin'?

Thank you for spotting this typo. We have corrected the spelling for Epicatechin.

11. Page 14; 'we analyzed ATP hydrolytic activity in tissues expressing different amounts of ATPIF1 (Figure S2H).' - where did data showing different levels of expression in different tissues come from? I am surprised there is no reference to PMID: 19559621

The Western blots detecting ATPIF1 in different tissues are shown in new Figure EV2K, including mouse liver, WAT, BAT, heart, and skeletal muscle (quadriceps) using the rabbit polyclonal anti-ATPIF1 (1:1000, #13268S; Cell Signaling Technology). We thank the reviewer for pointing that this relevant reference was missing. We have now included the reference suggested by the reviewer.

12. Fig 2- I'm not convinced that you really need to show all panels to 1A;

We thank the reviewer for his/her suggestion. We have now simplified Figure 2, we have removed the "after fixing" panel. We kept the dose dependency, as it really speaks to a specific action of Epicatechin in complex V activity.

13. 2D and E could go to supplementary. Its not clear in the text what preparation was used for these measurements nor how were they made - are these intact cells or isolated mitochondria? Measured using Seahorse?

We have simplified the message of both panels consolidating them in a single panel in the new Figure 1J. We have used isolated mitochondria from mouse heart and performed respirometry using Seahorse standardized protocols. The data are presented as percentage of state 3 OCR (substrates plus ADP) of the untreated mitochondria, as well as of maximal respiration (over FCCP untreated). We have now clarified the type of samples used in the text and figure legend.

14. Effects of catechin (Fig 2F-I) are all negative - can these go to supp?

We have now moved all the panels related to catechin to the On-line Appendix.

15. Page 13 and Fig 3: 'ATPIF1 inhibition' sounds like it means inhibition of the action of ATPIF1 but I think what you are doing is looking at inhibition of ATP hydrolysis by ATPIF1? I am surprised that you do not refer to PMID: 18590689
here?

We thank the reviewer for this point. We have modified the text and made it clear that we are looking at inhibition of ATP hydrolysis by ATPIF1. We have also included the suggested reference when we first introduce ATPIF1 in the text.

16. Fig 3 explores the action of added recombinant ATPIF1 but we don't know how much endogenous protein there is? Or have I misunderstood? How do you know that endogenous levels are not already close to saturation?

The reviewer is right that there is still endogenous ATPIF1 in the samples that we are using. However, the addition of the recombinant IF1-GFP is still able to bind to CV and inhibit ATP hydrolytic activity in a dose dependent manner. This inhibition by IF1-GFP demonstrates that we are purifying a mixed population of complex V molecules, some with and other without IF1 bound. This is consistent with previous publications showing that not all complex V has IF1 bound after its purification. Otherwise, no ATP hydrolyzing activity could be detected from these preparations.

17. Do you really need Fig 3L? move to supp?

We agree with the reviewer and have moved this set of data to supplementary, now Figure EV3B-D.

18. I think Fig 3M needs to be quantified?

We have now quantified this set of experiments and included the data in the new Figure 3G.

19. Page 15 when introducing the H49K mutation worth stating that this is pH insensitive.

We thank the reviewer for his/her observation and we have now stated that the interaction of mutated IF1 is insensitive to pH.

20. 'In cells expressing either ATPIF1-WT or H49K', should probably read 'overexpressing'?

We have now fixed the text accordingly.

21. Fig 4F I don't understand why EPI fails to cause depolarisation in the presence of AA if the membrane potential is being maintained by reversal of the synthase? If EPI selectively inhibits the reversed synthase activity you would expect potential to decrease? This is incorrectly referred to as Fig 4G in the text.

We agree with the reviewer. We believe that we are not seeing depolarization in the presence of antimycin for two reasons: 1) Antimycin A induced depolarization in these cells is minor at high concentrations (5μM, 14%). Also, the expected response to oligomycin is only observed at AA 5μM and Oligomycin 5μM. These data support that our membrane potential measurements are not as sensitive to ATP hydrolysis induced by AA treatments that effectively block respiration (Figure EV3E) in these same cells. To show this in more detail, we performed a new experiment using different concentrations of oligomycin (see below, Figure 3 for reviewers), showing that even with complete inhibition of ATP synthase by oligomycin the membrane potential measurement is not efficient to demonstrate a direct effect of hydrolysis. Note that, only at high concentrations of AA and oligomycin, we can see further depolarization by oligomycin. In addition, this approach is integrating the entire mitochondrial population, meaning that there could be some mitochondria being ATP hydrolyzing mitochondria and others depolarizing because of increased ROS (which would be a depolarization insensitive to oligomycin and EPI treatment). These multiple factors contributing to AA induced depolarization and membrane potential preservation is what prompted us to measure mitochondrial ATP levels, as those would be more sensitive to changes in ATP hydrolysis than membrane potential. Consequently, our main motivation to show the membrane potential data was to support our conclusion that mtATP levels are more sensitive to changes in ATP hydrolysis than membrane potential. This new set of data has been included in Figure EV3D.

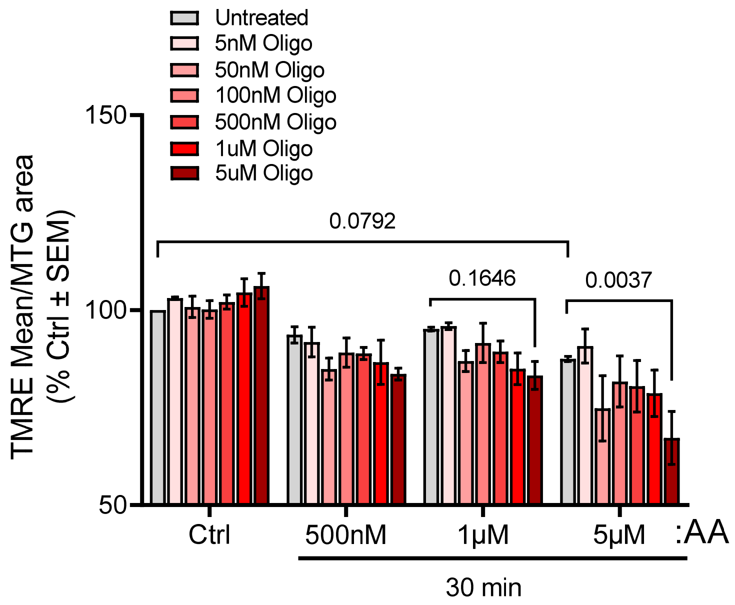

Figure 3. Dose dependent effects of oligomycin on antimycin A-induced depolarization. Membrane potential measured in intact cells as the ratio of TMRE/MTG fluorescence. Oligomycin induced depolarization is only observed in cells treated with 5 μM antimycin and only using 5 μM of oligomycin. These high concentrations support that these membrane potential measurements are not very sensitive to ATP synthase reversal to hydrolyze ATP.

22. From Fig 4 G onwards this is referred to as the 'ATP fluorogenic dye' - what dye is this? Has it been validated? It is misleading to describe this as a more specific measure than TMRM - it is measuring

something different - presumably. I have no idea Fig 4G mtATP measurements - no idea what is being measured or how this works and is really unconvincing.

We thank the reviewer for these questions, as it made us realize that we did not describe this method in sufficient detail. Indeed, TMRE is the most specific dye available to measure membrane potential. What we wanted to state is that changes in intramitochondrial ATP levels can be more sensitive to changes in complex V hydrolytic activity. The reason is that when the ATP synthase reverses, it stops synthesizing ATP and it starts consuming ATP, which in combination decrease ATP concentrations. The membrane potential measurement will only be sensitive to ATP consumption and will be mostly insensitive to a blockage in ATP synthesis.

We want to emphasize that we are not the first to use this probe to measure mtATP. This probe has been independently validated by other research groups. The mtATP probe was first described by Wang L et al (PMID: 26676712) and then by Rieger et al (PMID: 34595823, Fig.9C). Using this probe, Rieger et al. demonstrated that mtATP levels increased when hydrolysis was inhibited by overexpressing the dominant-negative IF1-H49K and that these levels decreased when IF1 was knocked out.

The arguments over whether ATP is imported through the reversed ANT is all confusing and adds unnecessary complication to an already complex paper. I don't understand why you don't simply use FCCP to drive maximal reversal of the synthase and promote hydrolysis?

Conceptually, FCCP is more effective depolarizing mitochondria than antimycin A and theoretically, FCCP should be more efficient activating ATP hydrolysis. We performed the experiment suggested by the reviewer and we observed the same trend of EPI compensating for FCCP-inducing depolarization on mtATP levels. But the efficiency of EPI under FCCP treatment was lower than AA-treated cells, for which we can just speculate that not all FCCP induced depolarization can be compensated by the partial inhibition of hydrolysis by EPI. Indeed, FCCP depolarizes by aiding proton to diffuse across the membrane, while antimycin A depolarizes by blocking proton pumping.

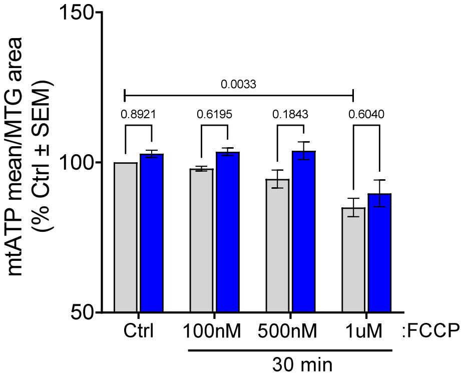

Figure 4. Effect of EPI on internal mitochondrial ATP pool under conditions where mitochondrial respiration is inhibited by antimycin A. Mitochondrial ATP (mtATP) content measured as fluorescence intensity per mitochondria area and shown as % of control untreated (n=3). Control fibroblasts were incubated for 30min with DMSO or EPI in different concentrations of FCCP. Mitochondrial area was quantified using MTG staining.

23. I have concerns and reservations about all subsequent measures of 'mtATP' as it isn't clear what is being measured.

We now provide a detailed description of this probe in the manuscript and cite the original reference describing the probe (PMID: 26676712), as well as the paper that used this probe to determine the role of IF1 on mitochondrial ATP levels (Rieger et al. PMID: 34595823).

24. Fig 4K - the effects of EPI on lactate seem insignificant. A, I missing something?

We agree with the reviewer and have now moved this panel to supplementary (Figure EV3J).

This experiment provided functional evidence that fibroblasts from patients with a mitochondrial disease had an increase in glycolysis to compensate mitochondrial dysfunction. In addition, the major goal was to determine whether EPI treatment could decrease the glycolytic flux, as EPI should decrease the demand for glycolysis by making more ATP available. A tendency is observed for CIII-deficient cells, but this change is not observed when ECAR or OCR were measured, meaning that the observed tendency is not reflecting a decrease in glycolysis (Figure EV3J). The fact that we do not see a decrease in glycolysis supports that glycolysis to lactate is not only driven by ATP demand in proliferating cells, but by other biosynthetic needs provided by mitochondria.

25. Page 21- what do you mean in cells 'with endogenous levels of ATPIF1'? with high levels? Low levels?

We have now fixed the text accordingly: “BCS1L shows low endogenous levels of ATPIF1”.

26. P22 I guess you don't really mean 'no addictive effect of EPI'?!

We have now fixed the text accordingly. The sentence now reads “no additive effect of EPI”

27. I'm afraid that I don't feel qualified to comment on the mdm mouse experiments- my overall impression is that these are as convoluted and confusing as the rest of the paper. I am concerned that epicatechin has been shown to have multiple effects in cells and tissues and so interpreting changes in an animal model specifically in relation to effects on the ATP synthase may be difficult.

We thank the reviewer for this comment. We have performed additional experiments *ex vivo*, using DMD cells as well as isolated mitochondria from *mdx* mice, where we show that EPI can restore membrane integrity. These experiments support the relevance of EPI direct actions in the mitochondrial ATP synthase of DMD muscles. These data further support our previous data showing the absence of metabolic effects induced by EPI treatment in WT mice, where ATP hydrolysis is not exacerbated. Indeed, the absence of an effect of EPI treatment in WT mice demonstrates that EPI is incapable of blocking complex V activity when it is synthesizing ATP.

Overall I urge the authors to try to trim down this paper as much as they can at all levels to try to improve clarity and reduce 'noise'.

We followed this recommendation: we moved multiple figures to the supplementary and to a new manuscript, where the ATP hydrolysis protocol is described in detail. We have shortened the text as well.

Referee #2:

Summary. The studies reported in this manuscript detail the affect of epicatechin on the activity of the ATP synthase. Specifically, the authors conclude that epicatechin inhibits ATP hydrolysis, but not ATP synthesis, of the ATP synthase by binding to the same site as the natural inhibitor protein, IF1. Furthermore, the studies suggest that epicatechin can help in the treatment of diseases that affect the electron transport chain and the ATP synthase, including Duchene Muscular dystrophy. In this context, epicatechin, is shown to increase the ATP content in the mitochondria and improve muscle force without an increase in mitochondria content.

Review: Overall, this is certainly an important study and may provide a new drug that may modulate the disease phenotype of subset of mitochondrial diseases. A problem I had was that the number of experiments presented was extremely dense. As a reviewer, I felt obliged to assess each figure, but as a reader, I would not have. There were some studies that I thought were ancillary and would be better omitted. Further, there are issues I had with the interpretation of the results. The authors certainly have a strong background in oxidative phosphorylation, and yet I struggled with understanding some of the studies. I must admit that I have never used a Seahorse XF96, which might add to some of my confusion, but at least on the surface, I understand the workings of the Seahorse.

With that stated, I will spend some time on some of my difficulties. (I am a little disappointed that there are no page numbers or line numbers, which makes referencing that point more difficult.)

We apologize for the inconvenience this might have caused. We have now included page, line and figure numbers in the revised version of the manuscript.

The results start with talking about protocol to measure ATP hydrolysis and ATP synthesis at the same time.

The statement is made that 2 protons are net transported with the hydrolysis of ATP and that is referenced. But we know that there are 8 c-subunits in the ATP synthase, so that number is 2.67. But maybe the term "net" transport is important. With the hydrolysis at ATP at pH 7.0, there are 3/4 H+ released, due to the formation of Pi and ADP. In addition, there is an electrogenic exchange of ATP for matrix ATP, when measuring ATP hydrolysis. There are also some other electrogenic pump, including the Pi transporter that brings in H+ into the matrix. But these issues become important later. Lastly, it should not be ignored that the respiratory chain pumps out protons.

We acknowledge the reviewer for his/her deep understanding of proton pumping and stoichiometry of ATP synthesis and hydrolysis. We did not include a discussion on the stoichiometry and contribution of other proton exchange mechanism to avoid a convoluted discussion. To acknowledge this complexity and the importance of stoichiometry in ATP hydrolysis and synthesis, we have referred to authors to read the following reference summarizing these issues (PMID: 20858734).

In Figure 1, there are an oxygen consumption studies that shows state 3 and 4 respiration. First, it would have been good it the P/O ratio was reported. In Fig. A top, there are points for state 3 and state 4 respiration. There are 2 points before ATP is added. For State 3 control, the rate of respiration is steadily decreasing and may have continued, but ATP was added before the rate stabilize. It is not clear what the addition of ATP did, if anything.

We thank the reviewer for this important point. We provided an explanation for this decrease during the second measure of state 3 (see Figure 1 for the reviewers). The explanation for this decrease supports that ATP would further accelerate the decrease in respiration and the simultaneously increase in the acidification rate, as a result of the decrease in mitochondrial ATP synthesis (which consumes protons, see response to reviewer 1).

But, state 3 respiration requires the addition of ADP, and by adding ATP, that might remove ADP from the matrix, basically shifting it to state 4 with ATP.

Indeed, we agree with this statement. This was the purpose of adding ATP, to make sure that the acidification rate was not being modified by ATP synthesizing respiration consuming protons. Thus, the addition of ATP ensures that acidification is exclusively reporting on ATP hydrolysis.

We agree with the reviewer that it is not a straight-forward explanation than can be easily deduced from just looking at the graph. State 4 mitochondria have been in a hyperpolarized state of high ROS generation for more than 30 minutes, when compared to state 3 mitochondria. Complex I is extremely sensitive to ROS and RET (reverse electron transport) (PMID 27052170; PMID 27348412; PMID 27076081) inducing its degradation. However, complex V is not degraded by ROS. This difference in hyperpolarization sensitivity explains why oligomycin-sensitive acidification after FCCP injection is the same in state 3 and state 4 mitochondria, as complex V hydrolyzing activity is not altered by an extended hyperpolarization and ROS.

In Fig. 1A bottom, it looks at the acidification of the buffer. As before, there are 2 time points before ATP addition, and it is unclear what ATP addition did.

The 2 time points reflect the changes in ATP-synthesizing respiration. As addressed in one of the responses to reviewer 1, the second acidification rate value measured before the injection of ATP is less negative, because there is a decrease in mitochondrial ATP -synthesizing respiration. As a result, less protons are consumed by the ATP synthase and therefore, the acidification rate becomes less negative. The oligomycin sensitivity of the acidification rates after ATP addition demonstrates that complex V activity hydrolyzing ATP is responsible for ATP induced acidification. The reviewer raises an excellent point on why state 4 mitochondria can further increase acidification rates in response to ATP. This is explained by the fact that the mitochondrial preparations are not pure and still contain some endogenous ATP, ADP and contaminant ATPases. This means that we cannot prevent the generation of some endogenous ADP in state 4 mitochondria. However, the contribution of this endogenous ADP production is overridden by saturating ATP addition to increase acidification rates. It is important to note that the fold increase in acidification rates induced by ATP injection in state 3 mitochondria is larger than in state 4 mitochondria, as expected from the blockage of maximal ATP synthesis with maximal proton consumption. The fold increase in state 4 is much lower because ATP synthesis is already blocked and some hydrolysis can occur from endogenous ATP.

The addition of FCCP caused a sharp rise in acidification. The reason for this is as stated earlier: when ATP is hydrolyzed, it releases 3/4 H+. The reason they give for this increase in ATP hydrolysis relates to the conclusion they are trying to press that in mitochondria, there are complexes that are active in ATP hydrolysis and those in synthesis. While this may be correct, the increase in the rate of ATP hydrolysis here is that the system is uncoupled and ATP hydrolysis is favored over synthesis and the protons feely flow from the matrix to the buffer solution.

We thank the reviewer for these great points. The addition of FCCP indeed causes a rise in acidification, by depolarizing the mitochondria and forcing all molecules of ATP synthase to do hydrolysis. In this regard, the reviewer is right that the free flow of protons from the matrix to the buffer characterized by uncoupling could change acidification rates independently of complex V. However, oligomycin treatment effectively prevents any acidification induced by FCCP, which effectively demonstrates that the flow of protons to the media is controlled by mitochondrial ATP synthase, and media acidification by FCCP is not a result of an uncontrolled flow of protons.

There are also some studies using Proteanase K that as used to show that the mitochondria were intact. But we are more concerned about the coupling, and the mitochondria can be impermeable to proteinase K, but not protons.

The responses of OCR to ATP, oligomycin and FCCP injections demonstrated that these fresh mitochondrial fractions are coupled. If fresh mitochondria were uncoupled or poorly coupled, the responses to ATP, oligomycin, and FCCP would be inexistent and poor. Indeed, the insensitivity to proteinase K further confirmed the absence of a large damage to the membrane explaining ATP hydrolysis. Given that the proteinase K is just a confirmation of the OCR profiles shown in the main Figures, we moved the proteinase K data to the supplementary to make the manuscript less convoluted (Figure EV1B).

I had some issue with their use of "frozen" mitochondria. I think it would be better if they referred as "previously frozen" and detail whether what as frozen - the tissue or the mito. At the end of the para, they state "Addition of oligomycin at different concentrations in the assay media using frozen mitochondria revealed a dose dependent inhibition of ATP hydrolysis (Figure 1F), demonstrating the specificity of the assay." I'm not sure what they were saying other than there are not any other ATPases present.

We agree with the reviewer in both observations. We have now fixed the text to indicate previously frozen samples instead of just frozen samples. Regarding Figure 1F, we have completely removed it from the manuscript and included it in a new manuscript that describes this new method measuring ATP hydrolytic activity (under review in LSA, LSA-2022-01628).

Before the next section of in silico screening, they state: "We identified down regulation of ATPIF1 together with increasing ATP hydrolytic capacity as a common adaptive mechanism in mitochondrial diseases. This mechanism would allow for cytosolic ATP consumption to maintain membrane potential and consequently prevent eventual apoptotic cell death (Chen, Birsoy et al. 2014)." But this statement is in complete conflict with the conclusions in this study. In this study, they suggest that inhibition of the ATPase activity is better for the cell. But the statement above, suggests that opposite.

We agree with the reviewer that this statement could be confusing. We meant to say that this mechanism (ATP hydrolysis), when it is adaptive, it can prevent cell death. However, in some mitochondrial diseases, this mechanism can be maladaptive and do more harm than good (consuming more ATP when this consumption is not needed to prevent cell death). To avoid any confusions, we removed this statement.

The Fig. 1I would be better if the interaction of Epicatechin with the F1 was shown. It also would have been good if the inhibition assay was done using isolated F1. In 1K, it is shown that Epicatechin only inhibits ATPase activity by 30% even at 0.1mM (Fig. 1L). If it is acting like IF1, then that must mean that it is displaced from the binding site - that the affinity is not high. This comes in later.

In Figure 3, we demonstrated IF1-EPI competition using isolated complex V treated with EPI and purified IF1-GFP. These data support the binding of EPI in the IF1-binding site predicted by the computational modelling shown in Figure 1G. In addition, EPI treatment blocked ATP hydrolysis activity in the same purified complex V, measured using in gel activity assays (Figure 2). Thus, Figure 3 validates the modeling data shown in 1G and provides the mechanism for the decrease in ATP hydrolysis induced by Epicatechin treatment shown in Figure 2. It was a possibility to move Figure 1I to Figure 3. However, such a move would make the text more convoluted.

The experiments in Fig. 2 were done to reveal more basis for the interaction of Epicatechin with the ATP synthase. I would have liked to see more direct studies on this question. In Fig. 2A, the EPI is added to the mito, the enzymes extracted in detergent, and then run on a BN gel. The ATPase activity was measure in the gel, with 3 hr incubation. The inhibition of activity is shown to be much dramatic than 30% - this is despite the fact that the enzyme complex has been run through a BN gel without EPI. This is totally unexpected and suggests that something more has happened. Further, the 3hr time frame hard to interpret as for comparison, it is not clear how linear activity is over that time period.

We thank the reviewer for this important remark. We believe that it is not totally unexpected that EPI preserved the interaction with complex V after running the gel. We provide evidence that Epicatechin is binding to the same site as IF1 and it can even compete with IF1. Therefore, if the blue native gel conditions preserve the interaction between endogenous IF1 and complex V, why is it totally unexpected that the interaction with EPI is preserved after running a blue native gel? Another valid interpretation is that preserving the inhibitory effect is further proof that epicatechin binds to complex V with a similar force than IF1. Furthermore, this is not the only experimental set that supports the direct interaction of EPI with complex V in the IF1 binding site. Here a list of the other evidence shown:

1) Computer modelling approaches using complex V structure.
2) Epicatechin added to the reaction buffer used in the gel to measure ATP hydrolysis activity in purified complex V.
3) Competition experiments between purified complex V, purified IF1 and EPI treatments added before and after adding IF1 to the reaction.

For Fig. 2E, they state: "Changes in state 3 but not in MRR confirm that EPI is targeting ATP synthase." But this is not quite correct. It is possible that EPI altered ATP/ADP transport or maybe Pi transport. Maybe more.

The reviewer is correct and there could be other components that might explain a change in state 3, but not in maximal respiration. This statement came after presenting Figure 1G, which shows that computer modeling and unbiased approaches already identified that Epicatechin binds to the IF1-binding pocket in mitochondrial ATP synthase. To be more accurate and acknowledge these possibilities we changed our statement to: “An increase in state 3 respiration that is not accompanied by an increase in MRR supported our interpretation that EPI modulates ATP synthase”

For Fig. 2J-K, the question being addressed seems to be pretty simple. I say this because the figure adds to the work the reader has to do.
The remaining experiments are slight variations of the initial experiment and don't seem to provide much information - at least relative to the amount of effort is takes to go over them.

We believe it is important to use different approaches to confirm that EPI is inhibiting ATP hydrolysis by direct binding to complex V and that this interaction is not mediated by other facilitators. We agree though that the figure had an excessive number of panels and we followed this suggestion of the reviewer: we have now simplified Figure 2 and moved some panels to supplementary. Despite moving these panels, the current figure still shows that Epi blocked complex V hydrolytic activity in intact mitochondria, uncoupled mitochondria and in purified complex V. Such a sequence is needed to illustrate a direct action of EPI on complex V.

For Fig. 3, they state: To compare the two mechanisms of action, we performed similar experiments where we block ATPIF1 activity with the specific inhibitor BTB06584 (BTB)." But the paper they reference indicates that BTB "is an IF1-dependent selective inhibitor of the" ATP synthase. This is just the opposite of what is presented in this paper.
But even so, I don't see much value to the experiments in Fig. 3 as BTB is not the main discovery in this paper. I suggest that this figure is dropped.

We thank the reviewer for this comment and dropped this figure, as it is not providing any essential information to support the main findings of our study.

I also don't understand the use of IF1-GFP. Can this get into the mitochondria? If so, it wasn't shown. But I would not expect it to get in as the import of proteins in the mito is done during translation, and not post translation. Also, the GFP is folded, and even if it did get to the import machinery, it would not likely unfold to allow import.

We used IF1-GFP in previously frozen mitochondria and in Complex V purified from bovine heart. Mitochondrial import is inactive in previously frozen mitochondria and it does not exist in purified complex V. These approaches provided a key evidence showing that Epicatechin directly binds to complex V and in the IF1-binding site.

For Fig 4, the amount of information is small for the amount of effort it takes to go over the figures. There should be a control for the effect of EPI on PLA.

We have now rearranged the panels leaving in the main figure only those that are more relevant for the flow of the paper. We have also added the imaging and quantifications of EPI effect on PLA for the CIII-deficient cells with overexpressed levels of ATPIF1 (Figure EV4C-D). The overexpression of ATPIF1 is notable in the PLA levels, but as expected, no difference was observed when EPI was added.

In this set of experiments, they use the IF1 mutant, H49K - which they describe as "constitutively active". This is correct, as it has to do with the protonation of His which is essential for binding to F1. This has been the basic reason why IF1 has been proposed to be an inhibitor of ATPase but not synthesis since it cannot bind to the ATP synthase at pH 8.0, the pH during state 3 respiration. The authors contend that IF1 inhibits ATPase and hydrolysis, while this is not the prevailing view. The papers they cite are not entirely consistent with this idea. This is pretty central to the mechanism they are proposing.

We acknowledge the reviewer for his/her suggestions. Indeed, we contend that IF1 inhibits ATPase and hydrolysis to include all views present in the literature. However, our study does not try to solve the IF1 controversy. The main point that we want to make is that IF1 structure itself and its binding mode can block rotation of the ATP synthase in both directions. In the case of EPI, it is a much smaller molecule than IF1 and cannot behave like IF1 (stick on a wheel analogy). As we observe that EPI only blocks hydrolysis and cannot act as a "stick on a wheel", we only conclude that the mechanism of action of EPI should be somewhat different than IF1 despite sharing the binding site.

In Fig. 4F, G, it is stated: " Adding 1uM oligomycin further depolarized mitochondria, demonstrating that under AA, the mitochondrial ATP synthase was working in reverse. No changes were observed when EPI was incubated with AA, supporting the hypothesis that EPI will only act in the molecules of ATP synthase working in reverse and where ATPIF1 could be displaced (Figure 4G)." I think this is using data from Fig, 4F and 4G, but the assays are different - potential vs ATP content. So, I'm not sure the conclusion can made.

We agree that the statement of ATPIF1 displacement is speculative and our data does not provide unequivocal support to this statement. In addition, our data shows that mitochondrial ATP levels are more sensitive to changes in ATP hydrolysis activity when compared to membrane potential in our in vitro models of mitochondrial dysfunction. Therefore, we have removed this statement: "supporting the hypothesis that EPI will only act in the molecules of ATP synthase working in reverse and where ATPIF1 could be displaced".

In Fig. 4H, the basis for the importance of this m/s, the level of ATP is measure with a fluorescent dye. I would have preferred direct measurement, but the odd result is that the effect is not present at 1uM EPI but was observed at 50nM even though the effect on ATPase is much better at 1uM than at 50nM, see Fig. 2B, for instance.

We thank the reviewer for allowing us to clarify this issue. We believe that the difference in effectiveness between the data in Figure 2 and Figure 4 is explained by the use of isolated mitochondria from mice in Figure 2 and the use of primary human fibroblasts in Figure 4. It is common to lose efficacy at higher concentrations of compounds in more complex systems such as intact cells. In whole cells adaptive responses, such as multidrug resistance upregulation, are present and these mechanisms do not exist in isolated mitochondria. The specificity and sensitivity of this probe reporting mitochondrial ATP content has been validated by other laboratories (PMID: 26676712; PMID: 34595823)

Fig. 4J is very confusing as it is not possible to ID the points.

We have now changed the way the data were plotted to avoid any confusion.

At one point they say: "With these results, we propose that mitochondrial ATP hydrolysis is a common response to electron transport dysfunction." I think this idea is not unique and widely accepted as the potential gradient is essential for cell survival.

We agree with this point and this was a result from editing a longer sentence. The novelty of our data lies on the demonstration that ATP hydrolysis can be maladaptive and drive disease.

In the text, they say "...amelioration of the disease phenotype in CIII-BCS1L patient cells." I think this is a bit strong.

We agree with the reviewer that this statement is a bit strong. We have toned it down in the revised version and described the specific improvements observed in these cells.

In the text they say "... suggesting that ATPIF1 displacement can only be detected if sufficient ATPIF1 is expressed and bound to ATP synthase." I'm not sure what the point is here.

That displacement of ATPIF1 induced by Epicatechin can only be observed in mitochondria from tissues with IF1 expression. Therefore, Epicatechin effectiveness is expected to be higher in tissues where IF1 is not expressed. We decided to remove this speculative statement, as we did not compare Epicatechin efficacy in different tissues.

I think the data in Fig. 6 and 7, is the most important of the paper. But it took a long time to get to this point and I, was totally exhausted by the time I got to these figures. And it is not entirely clear how much of the data shows a significant difference between treated and not treated.

We have reorganized Figures 6 and 7 and added additional evidence of the effects of EPI in ATP hydrolysis in cell culture models of Duchenne's Molecular Dystrophy (DMD). These new data further support that Epicatechin acts in a muscle-autonomous manner. We believe that the important and significant effects of EPI in DMD models are much clearer now, considering also how we have simplified the rest of the figures to make the manuscript less dense and easier to follow and read.

This is stated a number of times "mitochondrial ATP Fluorescence Intensity". This is inaccurate. The exact dye used should be stated and maybe some reference as to how well it works.

We have removed these statements and substitute them by: "fluorescence of the mitochondria ATP probe". We now introduce this probe in more detail, citing the main paper showing the validation of this mitochondrial ATP probe (PMID: 26676712), as well as the use of the probe to determine the role of IF1 in mitochondrial ATP hydrolysis (PMID: 34595823).

I would also suggest being careful in using abbreviations. The writers assume that the reader recognizes the abbreviations. Please be careful to define them.

We have now provided an appendix containing all the abbreviations the manuscript and we have also made sure they are all appropriately defined.

I think it also important to indicate what the "n" means in each experiment. What do each "n" represent? Do they represent another assay with the same preparation or another assay with a second preparation?

We have defined n in each figure legend. Each "n" indicates biological/independent replicates. The value for each biological sample was a result of averaging technical replicates.

Overall, I would suggest making this paper more easily read. Do not include figures that are not to the point. Try to reduce the amount of data. This paper tries to do 2 things - show the mechanism of EPI and show that it helps in mitochondrial diseases. Maybe this is too much.

We thank the reviewer for his/her advice. We have now tried to simplify the paper and make it easier to read. Accordingly, we generated a second manuscript focusing exclusively on the description and validation of the ATP hydrolysis method.

Referee #3:

The mitochondrial F1FO ATP synthase, working in the reverse mode as an ATPase, preserves the mitochondrial membrane potential under conditions of low ATP. The authors of the study reasoned that an increase in mitochondrial ATP hydrolysis, rather than decreased synthesis, can be detrimental in mitochondrial disease and contribute to pathogenesis. Using isolated mitochondria and monitoring oligomycin-sensitive pH changes, the authors assessed ATP hydrolysis in parallel to ATP synthesis and OXPHOS activities. They show that in cultured cells about 20% of the ATP synthase works in the reverse mode and that this fraction increases in patient-derived fibroblast with OXPHOS deficiencies. An in silico screen identified (+) Epicathechin (EPI) as a modulator of the F1FO ATP synthase. Experiments using isolated mitochondria and cellular models indicate that EPI acts as a competitive inhibitor of ATPIF1. In contrast to ATPIF1, EPI selectively inhibits the ATPase but not the ATP synthase activity. Further experiments reveal that low dose EPI treatment restores total ATP content in patient fibroblasts and show beneficial effects of EPI on muscular performance in Mdx mice in vivo.
The demonstration that ATP synthase hydrolyzes ATP in respiring mitochondria is intriguing and supports the emerging view that the proton gradient across the mitochondrial inner membrane is heterogenous. Similarly, it is a novel and interesting concept to selectively inhibit ATP hydrolysis in mitochondrial OXPHOS disease. The beneficial effects of EPI treatment on muscle performance in Mdx mice underlines the potential of such an approach. At the same time, however, some of the conclusions of the authors remains unclear or appear premature and many issues need to be clarified.

Referee #3:

1. The authors provide evidence that EPI acts as a competitive inhibitor of ATPIF1. Modelling suggests binding to the same binding site, but why EPI only affects ATPase while ATPIF1 inhibits both ATPase and synthase remains unclear. Some of the descriptions of the EPI effects are rather confusing (´dependence on ATPIF1`?). The experiments shown in Fig. 3MN are not convincing and need quantification.

We thank the reviewer for this important remark. Indeed, the mechanism of action on how ATPIF1 can block synthesis at more basic pH (in respiring mitochondria) is still under debate. Thus, any statement trying to address this differential effect is going to be speculative. Our favorite hypothesis is that the differences in size and affinity between EPI and IF1 can explain this difference in activity. Another possibility is that the structure of the IF1 binding site might change when the ATP synthase works in reverse and maybe only then EPI can bind with sufficient affinity.

We apologize for the lack of clarity. Our intention was not to conclude that EPI would not be active if IF1 is present. We just wanted to describe our observation that EPI can compete with IF1 for the same binding site. Therefore, it is a possibility that IF1 content can determine the efficacy of EPI blocking ATP hydrolysis activity without affecting synthesis.

We provide now quantifications of the gels in new Figure 3, showing a statistically significant decrease induced by EPI treatment.

2. The authors should provide additional evidence that the detected pH changes reflect exclusively ATP hydrolysis by the F1FO ATPase. The authors argue that isolated mitochondria are used, but it remains unclear how other (oligomycin-induced) effects within mitochondria can be excluded (for instance, proton-coupled channels etc.)

We thank the reviewer for his/her observation. Measurements were performed in previously frozen mitochondria, where no proton gradient exists and as a result proton-coupled channels are inactive and cannot participate. In addition, both in intact mitochondria and in previously frozen mitochondria, oligomycin blocked acidification rates induced by the addition of ATP. Consequently, the changes in pH are specifically driven by the reverse action of F1F0 ATPase or Complex V.

3. The analysis of possible beneficial effects of EPI treatment under conditions of low ATP or in patient fibroblast is very limited. The authors show the expected increase in ATP levels (Fig 4) but how this affects cell proliferation or other metabolic parameters typically associated with OXPHOS deficiencies is not analyzed (even though stated on p. 17). This would be important to substantiate the idea of the authors that such an approach could be a novel treatment strategy in mitochondrial disease.

We thank the reviewer for his/her remark. We now provided the effects of EPI on cellular proliferation in fibroblasts from patients with OXPHOS deficiencies in new Figure 4I, showing that EPI increases proliferation in patients with CIII mutations. We have also included new experiments in Figure EV6D-F in human cell lines of Duchenne's Muscular Dystrophy (DMD) showing the beneficial effects of EPI treatment and ATP hydrolysis inhibition in membrane stability.

4. Related to this point, I have a conceptual concern: Increased ATP hydrolysis by F1FO is thought to maintain the mitochondrial membrane potential in OXPHOS deficiency or under low ATP conditions. It therefore appears difficult to envision that inhibition of the ATPase could have significant beneficial effects.

This is an excellent point. This concern is shared with any maladaptive mechanism that promotes disease. Our conclusion is not that ATP hydrolysis is unequivocally pathogenic or bad *per se*. It is only pathogenic when it is overactivated, surpassing the demand for the exact polarization needed. We would have never expected reversal to be maladaptive. However, the identification of the first selective inhibitor of ATP synthase reversal revealed to our surprise that in some cases, ATP hydrolysis is excessive and thus maladaptive. Furthermore, epicatechin treatments do not cause a complete inhibition of ATP hydrolysis. We report that partial inhibition promotes benefits. To clarify this important point, we added these statements in the discussion:

"Therefore, we propose that (1) acute adaptations decreasing ATPIF1 levels and reversing ATP synthase to avoid cell death, or (2) inhibiting the excessive hydrolysis of ATP in a "parasitic" way can be both beneficial in different disease models and serve as potential therapeutics".

5. The authors use different concentrations of EPI, ranging from 1 nM to 10 μM. The rationale remains unclear (for instance, 50 or 100 nM were used in some experiments in fibroblasts). Dose dependence is only shown for ATP hydrolysis, but not for ATP synthesis or respiration, precluding a direct comparison. Information is missing about how much EPI used in Fig. 2J, 3M, 4A, 5A and was administrated to mice in the experiments in Fig. 7.

We thank the reviewer for giving us the opportunity to clarify this concern. We have now indicated the concentrations used in each Figure panel, in their legends and in the methods section. We chose the lowest concentration that provided maximal blockage of ATP hydrolysis, after performing extensive titrations in isolated mitochondria and intact cells. We did not include the titration for ATP synthesis in the manuscript because we already show data on the effects of epicatechin on state 3 respiration and we have been currently advised to simplify the manuscript.

In marked contrast to isolated mitochondria and previously frozen tissue, in intact fibroblasts we observed that efficacy decreased at micromolar concentrations, explaining why we focused at 50 nM and 100 nM.

6. The manuscript contains many inconsistencies, typos, wrong figure callouts and is rather difficult to read. Some statements appear not justified. For instance, the authors state on p23L16 that EPI administration twice a day for 2 weeks was able to decrease susceptibility to injury in EPI treated mdx mice in a dose-dependent manner compared to vehicle (Figure 7A-C). However, the figures do not show

such a dose-dependent effect. On p17 in the caption of the section, it is stated in the caption that EPI treatment increases cell growth, but I could not find any data showing cell growth in Fig. 4 or S3. There are many errors in figure callouts. P16 last line, Figure 4F not 4G. P21, it seems that the order of figures (from 5G to 5I) is wrong in the figure. Legend to Fig. S3E is apparently missing.

We thank the reviewer for his/her observation and apologize for the missing callouts. We have carefully read the revised manuscript to assure that every figure callout is accurate.

Dear Orian,

All three referees have now written a report on the revised version of your manuscript. As you will see, they are all impressed with the work that has been put into revising the manuscript, and are still generally supportive of its publication in EMBO Journal. I would like you to respond to all the referees comments, paying particular attention to Reviewer 2's comments on the effect of oxygen availability on the rate of cytochrome oxidation. That said, I addition to this, there are a number of minor issues to be tackled.
I am looking forward to reading your manuscript after this second round of revisions. Please let me know whether you would like any input from me during this process.

Best wishes,

William

William Teale, PhD
Editor
The EMBO Journal
w.teale@embojournal.org

Instructions for preparing your revised manuscript:

Please make sure you upload a letter of response to the referees' comments together with the revised manuscript.

Please also check that the title and abstract of the manuscript are brief, yet explicit, even to non-specialists.

When assembling figures, please refer to our figure preparation guideline in order to ensure proper formatting and readability in print as well as on screen:
https://bit.ly/EMBOPressFigurePreparationGuideline
See also guidelines for figure legends: https://www.embopress.org/page/journal/14602075/authorguide#figureformat

At EMBO Press we ask authors to provide source data for the main manuscript figures. Our source data coordinator will contact you to discuss which figure panels we would need source data for and will also provide you with helpful tips on how to upload and organize the files.

IMPORTANT: When you send the revision we will require
- a point-by-point response to the referees' comments, with a detailed description of the changes made (as a word file).
- a word file of the manuscript text.
- individual production quality figure files (one file per figure)
- a complete author checklist, which you can download from our author guidelines (https://www.embopress.org/page/journal/14602075/authorguide).
- Expanded View files (replacing Supplementary Information)
Please see out instructions to authors
https://www.embopress.org/page/journal/14602075/authorguide#expandedview

Please remember: Digital image enhancement is acceptable practice, as long as it accurately represents the original data and conforms to community standards. If a figure has been subjected to significant electronic manipulation, this must be noted in the figure legend or in the 'Materials and Methods' section. The editors reserve the right to request original versions of figures and the original images that were used to assemble the figure.

Further information is available in our Guide For Authors: https://www.embopress.org/page/journal/14602075/authorguide

We realize that it is difficult to revise to a specific deadline. In the interest of protecting the conceptual advance provided by the work, we recommend a revision within 3 months (6th Feb 2023). Please discuss the revision progress ahead of this time with the editor if you require more time to complete the revisions. Use the link below to submit your revision:

https://emboj.msubmit.net/cgi-bin/main.plex

Referee #1:

This paper is very much improved after reshaping. I think it is an interesting story. There are a few minor issues that still need attention perhaps residuals left after the heavy editing.

1. In highlights: you say epi inhibits CV mediated ATP depletion independent of respiratory chain dysfunction: it's not clear what this means - what depletion? I think what you mean is prevents ATP hydrolysis as net ATP is increased? You also state that 'ATP hydrolysis by ATP synthase (CV) can occur in coupled mitochondria' but really this message is lost in the paper.
2. Abstract - CIII needs to be defined as complex III.
3. Fig 1E axis label still reads IF1/ATP5A/vinculin. Your point by point states that this is just to ATP5A - ? which is correct?
4. Fig 2C needs to be quantified?
5. Line 228 - you say that you ran BNGE to exclude effects on assembly or dimerization but don't mention whether there were changes in assembly or dimerization until much later raising the obvious question in the reader's mind.
6. The statement about catechin on line 234 seems a bit lost as you haven't discussed actions (or lack of actions) of catechin elsewhere?
7. I am puzzled by the numbers on the axes of a number of graphs - Figs 2B, E, G; Fig 3B, H, Fig 6 A, C, Fig 7B all label the axis as a '% of untreated' but the numbers are all <1 : have they been normalised to 1 rather than as a %? in 3B, the biggest effect reads as '0.2% of untreated' - how can that be right? If the untreated is almost 0 and the 1/50 is quite a dark band on the blot, surely this is an increase by several fold rather than a tiny fractional increase? This issue extends throughout the figures - Fig EV1 B and D, EV3 EFG, EV6 BCDE...
8. I find Fig 3H quite hard to interpret - it shows EPI hydrolytic capacity % inhibition : firstly I don't understand the numbers - do you really mean 0.1% inhibition of ATP synthase? And why are some points at almost no inhibition at all? How was ATPIF1 expression quantified (i.e. what do these numbers mean? 0.8 of what?)
9. I am curious about the sentences: (line 300 onwards) 'These results suggest that EPI cannot displace ATPIF1 bound to CV but rather prevent ATPIF1 binding to CV. As such, EPI is more likely to be an effective inhibitor of CV hydrolytic activity in conditions where CV binding sites to ATPIF1 are unsaturated'. Isn't this quite important in relation to potential therapeutic applications as one is looking to inhibit ATPase reverse activity in pathological states in which ATPIF1 is likely to be bound to CV?
10. The mismatch between the ability of EPI to preserve intramitochondrial ATP while failing to stop proton pumping so that it doesn't cause depolarisation in AA treated cells still doesn't make sense to me and I am unconvinced by the arguments. If the compound inhibits ATP hydrolysis sufficiently to preserve ATP it must inhibit proton pumping as well - the failure to see this makes me question the measurements, and even with the new data, suggest to me that this is just not the best assay. Is the issue simply that Epi is only a very partial inhibitor of the ATPase reversal?
11. Here you haven't explained how ATP is measured - an issue I raised in the first review and which you claim to have addressed in your rebuttal. On line 318 we are rather abruptly told that mitochondrial ATP content was changed - there is no explanation of how this was measured and even in the Methods, this is very cursory and simply states how the cells were loaded. Do you simply measure fluorescence intensity? At what wavelengths? Is this a simple function of ATP concentration? I see that you introduce the method further at line 348 - this should come where the probe is first mentioned, otherwise you risk losing the reader.
12. In the rebuttal, you state that 'not all FCCP induced depolarization can be compensated by the partial inhibition of hydrolysis by EPI' - I wouldn't expect EPI to have any effect at all on the depolarisation induced by FCCP but it should limit the rate of ATP depletion, as does oligomycin (or ATPIF1).
13. A trivial point, but on Line 32 - I'd replace 'remarkably' with 'surprisingly'! It isn't remarkable but it is surprising!
14. It might make sense to show that loss of mtATP due to AA treatment is blocked by oligomycin.
15. Line 370- I don't really understand the statement 'This raised the question whether under these conditions, ATP synthesis by CV contributes to the ATP pool'. The data show that ATP consumption tends to deplete ATP levels. This concluding statement seems a bit obscure and is then not followed up. Perhaps more importantly, part of the thesis suggested in the introduction and abstract is the suggestion that even in control (non disease) cells, some mitochondrial structures may be consuming ATP while others are generating it. If this were correct, you would expect to see a significant increase in total ATP in response to EPI even in control cells, and it seems that you do (Fig 4G), although small. I don't understand why you barely comment on this?
16. Lines 372 onwards: It is very odd that there is no measurable decrease in oxygen consumption in the Complex V disease model (is this heteroplasmic or homoplasmic? If the former, what is the mutant load?) You might expect membrane potential to be increased in this mutant. Fig EV3 again we have issues with axis labelling with % values shown as 1...
17. Line 440 - is the reduction on both CV and CII in the mdx due to loss of mitochondrial mass?
18. Line 459: 'The decrease in percentage of positive fiber after Evan's Blue dye detection in gastrocnemius (Hamer et al, 2002) in EPI treated mdx mice showed that EPI protected' what an odd way to phrase this? A decrease in Evan's Blue positive (damaged) fibres showed that...?
19. Did EPI increase ATP levels in the mdx muscle?
20. Line 509 - you talk about comparing epi ad catechin, but there are now almost no data on catechin in the paper.
21. Line 499 - I've no idea what 'a parasitic way' means in this context.
22. Line 520 'As no inhibition of synthase activity must be tolerable to be considered a good therapeutic approach' - meaning?
23. Line 537 'showed the best improvement in total ATP content and cellular proliferation rates of all the cells analyzed'. You

mean in response to epi?
24. Too many sentences simply aren't grammatically complete: line 550: Our results in mdx mice show that ATP hydrolysis EPI is sufficient to partially restore muscle force.

Overall there are a lot of awkward language and a lack of clarity especially in the introduction and discussion that tends to undermine the message a little. I suspect this is the result of heavy editing where it is easy to leave legacy text behind. A careful read and careful editing could much improve the paper.

Referee #2:

This is a revised version of the same m/s submitted in June, 2022. The authors addressed a large number of concerns identified by 3 reviewers.

Summary - from before. The studies reported in this manuscript detail the effect of epicatechin on the activity of the ATP synthase. Specifically, the authors conclude that epicatechin inhibits ATP hydrolysis, but not ATP synthesis, of the ATP synthase by binding to the same site as the natural inhibitor protein, IF1. Furthermore, the studies suggest that epicatechin can help in the treatment of diseases that affect the electron transport chain and the ATP synthase, including Duchene Muscular dystrophy. In this context, epicatechin, is shown to increase the ATP content in the mitochondria and improve muscle force without an increase in mitochondria content.

Review: Overall, this manuscript has the potential to be an important finding. However, the number of issues throughout the manuscript places some doubt on the entire findings. I think that the manuscript still needs to be cleaned up as to not drag down the significance of the findings.

The m/s is divided into to 2 sections. The first part tries to identify the mode of action of epicatechin and proposes that it inhibits the mitochondrial ATP synthase in a manner and at the same site, as the natural inhibitor protein. The studies demonstrating this conclusion are not straightforward and not direct. I believe that if this is the hypothesis, that direct studies on the purified F1 ATPase is the best and most direct approach as suggested earlier.

There are some issues that I have with the m/s and the author responses. I'll start with the author responses.

For Ref.1., and concerning Fig. 1A, it is proposed that the decrease in the oxygen concentration it the reason for the changes in the OCR. The arguments start with a number of indisputable facts, but not really related to the issue. The added figure in the response suggests that "excessive mitochondrial activity" which results in a lower oxygen concentration is responsible for the decrease in OCR.

But this cannot be correct. The affinity for oxygen by cytochrome oxidase is very high and the reaction is zero order until the very end - nearly anaerobic conditions. Since mixing by the Seahorse apparently reverses this effect, the mixing must bring some other substrate into less limiting conditions.

The second reason why this is not a correct conclusion is based on their data from the first version of the m/s. In Figure 1A, the OCR increases back to normal with the addition of FCCP. Thus, it is not an issue with the oxygen concentration.

I also do not like how the issue was solved by simply blocking out the data in the figures. The data is pretty important for the reader - if they are to try to interpret the results.

For reviewer 3, comment 2, in regard to oligomycin and frozen mitochondria. The authors respond that "no proton potential exists". But in fact, they frequently look at state 3 and 4. They also state that "both in intact mitochondria and previously frozen mitochondria". This is an example of a confusing statement that is frequent in the m/s. As I understand it, the "previously frozen mitochondria" are in fact, intact. This is an issue in many of the studies where I am not exactly sure what is being assayed.

Some of the issue in the text are:

Line 91. "confirmed" is too strong.
Line 129. "The increase in acidification happened in both in state 3 or state 4 mitochondria, confirming that ATP hydrolysis occurs independently of the presence of ADP."

I still find this confusing since the addition of external ATP might cause all ADP to be depleted in the matrix, so there would not be a state 3 rate at all.

Line 140. "ATP hydrolysis in mitochondria was approximately 20% of the maximal hydrolytic capacity measured in uncoupled mitochondria." I guess the point that is "remarkable" is that there was ATP hydrolysis at all. I don't find this so surprising as mitochondria are always a bit leaky. And when you add ATP, this likely gets exchanged for the ADP in the matrix. So, there is no

state 3 respiration.

Line 197. Quercetin does not inhibit ETC as such. It should be noted that it does not bind to the IF1 binding site. Neither of the 22 references support the claim.
Line 241. It would be good to indicate the concentration of mitochondria and IF1. Also, please distinguish between GFP-IF1 and IF1.
Line 254. Competes is a strong statement.
Line 258. What is F488?
Line 259. Competed is a strong statement.
Line 269. Oligomycin does not inhibit F1 as it is shown to do in this figure.
Line 329. Confirm is a strong statement.
Line 348. Is the signal linear? It also reports ADP, albeit not as strongly.
Line 381. ECAR is never defined and is not actually correct in this use.
Line 391. Not statistically significant, P=0.4. In fact, lots of the reported data are not significant.
Line 414. This is a rather remarkable conclusion that IF1 inhibits ATP synthesis in vivo.
Line 822. Competitive binding has not been demonstrated.

Overall, for all of the figure legends, I would recommend a more expansive description of the experiment, the amounts, the method, the sample, ... It is very difficult to decipher how the experiment was done.

Overall, this is certainly an important study and may provide a new drug that may modulate the disease phenotype of subset of mitochondrial diseases. However, the studies in the first part do it no service.

Referee #3:

The authors have improved the description of their findings in the manuscript and significantly increased its clarity. Although the authors have satisfactorily addressed most of my points, I am still not convinced that they can exclude that other proteins than the F1FO ATP synthase affect acidification in an oligomycin-dependent manner. Therefore, using acidification to monitor directionality of the ATP synthase might be an over-simplification. Regardless, I feel that the authors provide overall compelling evidence in support of their central hypothesis that the EPI treatment (and selective inhibition of the ATPase) can have beneficial effects, if the membrane potential can be maintained.
Two minor points: The authors specified now also the EPI concentrations used in the various experiments. I am wondering whether they always used (+) EPI in all experiments or also natural EPI (containing only small amounts of (+) EPI) or even commercially available (-) EPI, for instance in the mouse feeding experiments. The EPI isoform used should be clarified in all experiments. Finally, the Western blot in Fig. 7 showing OPA1 processing is of low quality and I am wondering how specific bands were quantified. This is however only of minor relevance for the conclusions of the authors and could also be omitted.

We appreciate the valuable comments from reviewers, which certainly improved our manuscript. We want to express our appreciation and gratitude for the unusual level of effort and time the referees have put into this manuscript. THANK YOU! We have now modified the text to address the reviewer's points and highlighted the main changes in the text and figures.

Referee #1:

This paper is very much improved after reshaping. I think it is an interesting story. There are a few minor issues that still need attention perhaps residuals left after the heavy editing.

We thank the reviewer for his/her suggestions and for appreciating our efforts in improving the manuscript, which has been substantially improved as a result after the important points highlighted by the reviewers.

1. In highlights: you say epi inhibits CV mediated ATP depletion independent of respiratory chain dysfunction: it's not clear what this means - what depletion? I think what you mean is prevents ATP hydrolysis as net ATP is increased?

Thank you for giving us the opportunity to clarify this highlight. Now it reads as: "*Selective ATP hydrolysis inhibition of CV can increase cellular ATP content without restoring respiratory function*." We believe that this highlight reflects better what we meant: that the decrease in cellular ATP content caused by respiratory dysfunction can be mitigated just by blocking CV hydrolytic activity, without correcting the electron transport chain defect.

You also state that 'ATP hydrolysis by ATP synthase (CV) can occur in coupled mitochondria' but really this message is lost in the paper.

We agree with this remark of the reviewer. The message is lost in the paper because our main finding is the identification of a selective inhibitor of CV hydrolytic activity, which can mitigate the bioenergetic and proliferation defects caused by respiratory dysfunction, as well as improve muscle function in Duchenne's muscular dystrophy. However, the new method that we developed to measure ATP hydrolysis revealed that ATP hydrolysis is occurring in coupled mitochondria as well. We are preparing a new manuscript in which we will describe the molecular mechanisms that explain this concurrency. Currently we are testing three possibilities: i) whether ATP hydrolysis can occur in different cristae in the same mitochondrion, ii) whether some individual mitochondria are ATP hydrolyzing while others are ATP synthesizing or iii) whether cristae and mitochondria heterogeneity in terms of ATP hydrolyzing activity co-exist.

2. Abstract - CIII needs to be defined as complex III.

We have now changed "CIII" to "Complex III"

3. Fig 1E axis label still reads IF1/ATP5A/vinculin. Your point by point states that this is just to ATP5A - ? which is correct?

We thank the reviewer for identifying this typographic error in the y axis. The graph is showing IF1/ATP5A levels, and we corrected the y axis.

4. Fig 2C needs to be quantified?

We have now included the quantification of Figure 2C and show that EPI does not change CV assembly.

5. Line 228 - you say that you ran BNGE to exclude effects on assembly or dimerization but don't mention whether there were changes in assembly or dimerization until much later raising the obvious question in the reader's mind.

We thank the reviewer for this comment. We have now described that EPI does not change complex V assembly or dimerization in page 12 as suggested. Now it reads:

First, we treated intact mitochondria with increasing concentrations of EPI. These intact mitochondria treated with EPI were then analyzed by blue native gel electrophoresis (BNGE), to determine whether EPI modulated ATP synthase assembly and in-gel hydrolytic activity (Acin-Perez et al, 2020a). EPI decreased in gel ATP hydrolytic activity in a dose-dependent manner (Figure 2A-B), without altering the assembly of CV (Figure 2C). These results show that EPI inhibition of ATP hydrolysis does not involve disruption of the assembled CV.

We also added this summary statement later in page 12, to further clarify it:

These data suggest that EPI directly binds CV to decrease ATP hydrolytic activity, rather than by modulating an endogenous CV regulator.

6. The statement about catechin on line 234 seems a bit lost as you haven't discussed actions (or lack of actions) of catechin elsewhere?

We agree with the referee about this statement. We have now removed it from the text.

7. I am puzzled by the numbers on the axes of a number of graphs - Figs 2B, E, G; Fig 3B, H, Fig 6 A, C, Fig 7B all label the axis as a '% of untreated' but the numbers are all <1 : have they been normalised to 1 rather than as a %? in 3B, the biggest effect reads as '0.2% of untreated' - how can that be right? If the untreated is almost 0 and the 1/50 is quite a dark band on the blot, surely this is an increase by several fold rather than a tiny fractional increase? This issue extends throughout the figures - Fig EV1 B and D, EV3 EFG, EV6 BCDE...

We apologize for the discrepancy between % and values being <1. All the values represent a change over the untreated control situation, with the untreated values being normalized to 1 (or

100% when expressed as percentage). We have now changed the values in the Y axis from 1 to 100% for consistency, where the control untreated is 100% instead of 1.

8. I find Fig 3H quite hard to interpret - it shows EPI hydrolytic capacity % inhibition : firstly I don't understand the numbers - do you really mean 0.1% inhibition of ATP synthase? And why are some points at almost no inhibition at all?

We acknowledge the reviewer for this comment, which is directly related to the previous point (i.e. labeling % when the control values were 1 instead of 100%). The Y axis refers to how much of the total ATP hydrolytic activity observed in a particular sample that is inhibited by EPI. We have now referred it as percentage, where 100% would mean that all the ATP hydrolytic activity in that sample can be inhibited by EPI.

How was ATPIF1 expression quantified (i.e. what do these numbers mean? 0.8 of what?)

The X axis is the amount of ATPIF1/vinculin referred as arbitrary units obtained by quantifying the bands on Western blot assays. We have now included this information in the Y axis.

9. I am curious about the sentences: (line 300 onwards) 'These results suggest that EPI cannot displace ATPIF1 bound to CV but rather prevent ATPIF1 binding to CV. As such, EPI is more likely to be an effective inhibitor of CV hydrolytic activity in conditions where CV binding sites to ATPIF1 are unsaturated'. Isn't this quite important in relation to potential therapeutic applications as one is looking to inhibit ATPase reverse activity in pathological states in which ATPIF1 is likely to be bound to CV?

We acknowledge the reviewer for this important point. We modified the text to make it less confusing, and now in page 15 it reads:

To further test the possibilities that EPI prevents ATPIF1 binding to CV as compared to displace the already bound ATPIF1 from CV we run a displacement and competition assay side by side. To test for displacement, we incubated purified CV with ATPIF1-GFP for 5 minutes to allow for binding to take place. EPI was then added to the mix for an additional incubation of 10 minutes before the sample was analyzed by BNGE to determine the amount of ATPIF1-GFP bound to CV (Figure EV2F). In a parallel competition assay, we incubated purified CV tetramers with ATPIF1-GFP in the presence of EPI. We found that EPI treatment did not displace ATPIF1-GFP that was already bound to CV. However, if EPI and ATPIF1-GFP were incubated concurrently, ATPIF1 binding to CV was decreased. These results suggest that EPI can prevent ATPIF1 binding to CV but cannot displace an already bound ATPIF1 from CV.

We agree with the reviewer that when CV is saturated with IF1, or at least completely occupied by IF1, the therapeutic efficacy of EPI will be reduced. However, we see a decrease in IF1 expression in fibroblasts from patients with mitochondrial diseases, such as complex III deficiency. Therefore, we expect that EPI will be efficacious in diseases showing a decrease in

IF1 expression coupled with excessive ATP hydrolytic activity. This is discussed as follows in page 27:

Furthermore, in our results with patient derived fibroblasts, the CIII deficiency due to BCS1L mutations that presented with lowest amount ATPIF1 bound to CV and correspondent higher hydrolytic capacity, showed the best improvement after EPI treatment in total ATP content and cellular proliferation rates of all the cells analyzed.

10. The mismatch between the ability of EPI to preserve intramitochondrial ATP while failing to stop proton pumping so that it doesn't cause depolarisation in AA treated cells still doesn't make sense to me and I am unconvinced by the arguments. If the compound inhibits ATP hydrolysis sufficiently to preserve ATP it must inhibit proton pumping as well - the failure to see this makes me question the measurements, and even with the new data, suggest to me that this is just not the best assay. Is the issue simply that Epi is only a very partial inhibitor of the ATPase reversal?

The short answer is, Yes, the reviewer identified the issue, which is that EPI is a partial inhibitor of ATP hydrolysis, while oligomycin, when used at high concentrations is a complete inhibitor. We rationalize that depolarization will require that the remaining levels of ATP hydrolysis will be lower than the levels of proton entry by ATP synthesis and uncoupling. And then, the difference between the two processes need to produce a membrane potential change that is above the noise for TMRE measurements.

Furthermore, the concept of "Oligomycin Reversal Point" where under some circumstances oligomycin depolarizes mitochondria that have impaired respiratory function has been studied in extreme conditions and always in very high concentrations of oligomycin, higher those required to inhibit oxidative phosphorylation.

We tried to explain better our point of view in the results session from page 16 onwards (see highlighted text):

Previous studies have demonstrated that ATP hydrolysis by CV is required to preserve mitochondrial membrane potential under conditions of impaired respiratory function when proton motive force is lost (Connolly et al., 2018). To test the effect of EPI on mitochondrial membrane potential we adapted the, previously well described, antimycin A model of inducing CV ATP hydrolysis. This model takes advantage of the opposite effect of oligomycin in ATP producing versus ATP hydrolyzing mitochondria. While oligomycin treatment in cells with intact oxidative phosphorylation results in mitochondrial membrane potential hyper-polarization, the addition of oligomycin to cells pre-treated with antimycin A to block respiration results in depolarization. This shift in oligomycin response, from hyper-polarization to de-polarization termed "Oligomycin reversal" represents the state at which membrane potential relies on CV ATP hydrolysis.

We can only see that oligomycin depolarizes mitochondria when used at high concentrations of both oligomycin and antimycin concentrations, with these concentrations being higher than the minimal concentrations required to see decreases in respiration. The need for high concentrations of oligomycin to induce depolarization, combined with the fact that EPI has lower efficacy than Oligomycin blocking complex V, explains why EPI cannot depolarize antimycin A treated cells. However, the effects on mitochondrial ATP levels without changes in respiration confirm the effects of EPI on mitochondrial ATP hydrolysis.

We also changed the conclusion of the membrane potential session in page 17 to expose the reviewer point:

To produce oligomycin reversal phenomenon we treated fibroblasts with increasing concentrations of AA for 30 minutes in the presence or absence of oligomycin and monitored membrane potential using the fluorescence membrane potential indicator, TMRE. As expected, while Oligomycin treatment alone led to hyperpolarization, the combination of AA and oligomycin at 5uM resulted in a statistically significant depolarization (Figure EV3E-F). Lower concentrations of oligomycin gave a dose dependent trend toward depolarization (Figure EV3F), which did not reach statistical significance. This result indicates that complete inhibition of CV is required to demonstrate oligomycin reversal. Surprisingly, this assay also identified 5uM AA concentration and 30 minutes duration are required to produce oligomycin reversal in these cells.

11. Here you haven't explained how ATP is measured - an issue I raised in the first review and which you claim to have addressed in your rebuttal. On line 318 we are rather abruptly told that mitochondrial ATP content was changed - there is no explanation of how this was measured and even in the Methods, this is very cursory and simply states how the cells were loaded. Do you simply measure fluorescence intensity? At what wavelengths? Is this a simple function of ATP concentration? I see that you introduce the method further at line 348 - this should come where the probe is first mentioned, otherwise you risk losing the reader.

We apologize to the reviewer for the lack of information, In the revised version in the methods section, we have now added a description of the probe and we have answered the questions raised by the reviewer.

Included in the methods section on page 37:

Mitochondrial ATP (mtATP) content was measured with the BioTracker™ ATP-Red dye, a live cell red fluorescent imaging probe for ATP. The probe specifically reports ATP content in the mitochondrial matrix of living cells. The probe without ATP forms a closed ring structure that is not fluorescent. In the presence of the negatively charged ATP, the covalent bonds between boron and ribose in the probe are broken and the ring opens, causing the probe to be fluorescent.

We also cited the first paper describing this method (Wang, Yuan et al., 2016), when introducing this methodology for the first time in the manuscript (page 17).

Do you simply measure fluorescence intensity? At what wavelengths? Is this a simple function of ATP concentration?

Fluorescence intensity of the mtATP probe (red channel) was measured in ROIs (region of interest) defined by mitochondrial staining with MTG (green channel). In this way, we eliminate any unspecific, non-mitochondrial, fluorescence contribution to our measurements. The fluorescence quantified is exclusively confined to mitochondria (MTG ROI). Excitation and emission filters used for the combination of dyes: Hoechst (ex. 360-400, em.410-480), MTG (ex. 460-490, em. 500-550) and mtATP Red (ex. 560-580, em. 590-640).

We have now included the explanation below in the method section (page 37):

Excitation and emission filters used for the combination of dyes: Hoechst (ex. 360-400, em.410-480), MTG (ex. 460-490, em. 500-550) and mtATP Red (ex. 560-580, em. 590-640). Analysis was performed with Perkin Elmer Harmony (Harmony 4.1) software by measuring the average ATP red fluorescence intensity inside a region of interested (ROI) generated by the MTG area.

12. In the rebuttal, you state that 'not all FCCP induced depolarization can be compensated by the partial inhibition of hydrolysis by EPI' - I wouldn't expect EPI to have any effect at all on the depolarisation induced by FCCP but it should limit the rate of ATP depletion, as does oligomycin (or ATPIF1).

We apologize for the confusion, which is caused by an incomplete sentence. The following underlined text was missing in this statement: "*not all the decrease in mtATP caused by FCCP induced depolarization can be compensated by the partial inhibition of hydrolysis by EPI*"

What we mean is that partial inhibition of ATP hydrolysis caused by EPI treatment could not completely rescue mtATP content in FCCP treated cells, as expected from the fact that ATP synthesis (CV in forward) is decreased by FCCP as well.

13. A trivial point, but on Line 32 - I'd replace 'remarkably' with 'surprisingly'! It isn't remarkable but it is surprising!

Indeed! We have replaced remarkably with surprisingly.

14. It might make sense to show that loss of mtATP due to AA treatment is blocked by oligomycin.

We thank the referee for this valuable suggestion. We have run each of the mutant cell models with and without oligomycin and with Antimycin A. The results demonstrated a complex pattern

where oligomycin effectively increases mtATP in all mutant cell lines but ineffectively under AA. We suspect that inhibition of respiration by AA and possibly the large levels of ROS generated by AA prevent the ability of oligomycin to restore mtATP. In examining the behavior of the mtATP dye under AA, we decided we need to better understand the effects of AA-induced depolarization, ROS and matrix pH changes on dye performance before AA experiment can be interpreted. We, therefore, suggest not including such an experiment in the current manuscript.

15. Line 370- I don't really understand the statement 'This raised the question whether under these conditions, ATP synthesis by CV contributes to the ATP pool'. The data show that ATP consumption tends to deplete ATP levels. This concluding statement seems a bit obscure and is then not followed up.

We apologize for this statement. It is unclear because it is hard for the reader to guess what "This" and "these conditions" was referring to. We removed this obscure sentence and added the following on page 19:

The above results demonstrate that, under impaired respiratory chain function, a depletion in cellular ATP is contributed by both impaired oxidative phosphorylation, as well as by CV ATP hydrolysis. As such, this is suggesting that the inhibition of hydrolysis alone is sufficient to improve ATP availability in conditions of impaired respiration. However, since the observed increase in ATP content could be the result of improved respiration, we further examined the effect of EPI on respiratory chain structure and function.

Perhaps more importantly, part of the thesis suggested in the introduction and abstract is the suggestion that even in control (non disease) cells, some mitochondrial structures may be consuming ATP while others are generating it. If this were correct, you would expect to see a significant increase in total ATP in response to EPI even in control cells, and it seems that you do (Fig 4G), although small. I don't understand why you barely comment on this?

We agree with the reviewer that this is an important point to be further discussed and we hope to address it in future publications. However, we would like to acknowledge the reviewer for this important observation, and we included now in page 18 onwards the tendency observed:

Remarkably, treatment with EPI resulted in a significant increase in total cellular ATP in two of the mutant fibroblasts tested, a CIII deficiency (CIII-BCS1L) and a CV deficiency (CV-ATP6) (Figure 4H) while in control cells a tendency is observed.

Furthermore, CIII-BCS1L also had increased proliferation rates after EPI treatment, with Ctrl and CV-deficient cells showing a similar tendency that was not statistically significant (Figure 4G).

16. Lines 372 onwards: It is very odd that there is no measurable decrease in oxygen consumption in the Complex V disease model (is this heteroplasmic or homoplasmic? If the

former, what is the mutant load?) You might expect membrane potential to be increased in this mutant. Fig EV3 again we have issues with axis labelling with % values shown as 1...

The patient sample had a 90% mutation load (Pastores, Santorelli et al., 1994), but no respirometry was provided in that publication.

However, mutations in ATP6, and particularly the mutation we are using in this manuscript, mtDNA 8993 T>G [Leu156Arg (L156R)], has been described to have heterogeneity in their respiratory profile depending on their mtDNA haplotype. This has been very well characterized by Manfredi's group where they generated different cybrid cell lines with exactly the same nucleus but different mitochondria coming from different patients in D'Aurelio et al, 2010 (D'Aurelio, Vives-Bauza et al., 2010). They reported that:

"The bioenergetic defects varied dramatically, not only among different ATP6 mutants, but also among lines carrying the same T8993G mutation"

Including in page 19:

As compared to control cells, BCS1L mutant cells shows a decrease in basal, maximal and ATP-linked respiration while the ATP6 mutant shows only an increase in maximal respiration (Figure 4H and Appendix 5), as previously reported (D'Aurelio et al., 2010, Fernandez-Vizarra, Bugiani et al., 2007).

In Appendix Figure 5, we are showing all the bioenergetic profile measured in these cell lines.

We have corrected all the Y axis and they are now reflecting 100% as the control/untreated values instead of 1.

17. Line 440 - is the reduction on both CV and CII in the mdx due to loss of mitochondrial mass?

We addressed mitochondrial mass using MTDR staining in homogenates as published (Acin-Perez, Benador et al., 2020) and found that there are no differences in mitochondrial mass. We have now included this data in Appendix section (Appendix Figure 6)

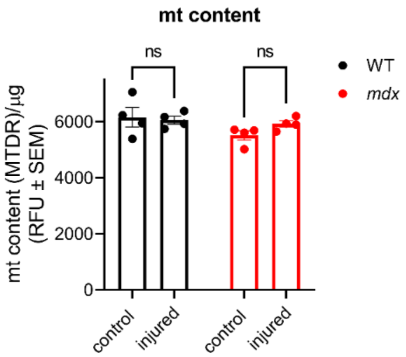

18. Line 459: 'The decrease in percentage of positive fiber after Evan's Blue dye detection in gastrocnemius (Hamer et al, 2002) in EPI treated mdx mice showed that EPI protected' what an odd way to phrase this? A decrease in Evan's Blue positive (damaged) fibres showed that...?

We have rewritten the segment as follows in page 23:

When mdx mice are expose to exercise induced muscle injury there is a dynamic process of plasma membrane damage and repair, during which the material from the interstitial fluid can enter the cell. The extent and duration of plasma membrane barrier breach is tested using Evan's Blue dye that enter the cell through broken plasma membrane (Hamer et al, 2002). Twenty-four hours prior to injury, mdx mice were injected with Evan's Blue and the number of Evans Blue positive cells was quantified post injury. EPI treatment decreased the number of Evan's Blue positive fibers, indicating that EPI protected the integrity of gastrocnemius muscle fibers

19. Did EPI increase ATP levels in the mdx muscle?

We agree with the reviewer that it would be very interesting to measure ATP levels in muscle. In this particular experimental set up, we did not have access to fresh isolated muscles (only frozen). Moreover, due to the quick turnover of ATP, the procedure requires for a very fast extraction followed by flash freezing of the tissue after euthanizing the animal. Alternatively, live-imaging experiments could be performed to measure ATP levels *in situ*. This is indeed a very interesting point and measurement that could be included in the future if we get access to fresh samples.

20. Line 509 - you talk about comparing epi ad catechin, but there are now almost no data on catechin in the paper.

We acknowledge the reviewer for his/her comment. In the first version of the manuscript, we had included a comprehensive analysis of the effects of catechin on mitochondrial function. The overall impression by all the three reviewers was that we should remove the data since it showed no effect of catechin on hydrolysis. We believe that is still important to include this set of data and they are now part of the Appendix (Appendix Figure 3). We are now referring to this data when pointing to the comparison between the effects of EPI and catechin in the discussion.

21. Line 499 - I've no idea what 'a parasitic way' means in this context.

We have now changed the sentence and now it reads on page 25: …*that leads to ATP depletion can be both beneficial in different disease models and serve as potential therapeutics*.

22. Line 520 'As no inhibition of synthase activity must be tolerable to be considered a good therapeutic approach' - meaning?

We acknowledge the reviewer for this observation. We mean that an approach that blocks both forward and reverse activity of CV, like oligomycin, cannot be used as a therapy. Blocking ATP synthase rotation in both directions will block ATP synthesis as well, being toxic.

We removed this part of the text and kept only as (page 26):

Knowing the mechanism and pharmacokinetics of the drug selected is essential when considering therapeutic approaches. For the above-mentioned compounds, pharmacokinetics is unknown.

23. Line 537 'showed the best improvement in total ATP content and cellular proliferation rates of all the cells analyzed'. You mean in response to epi?

Yes, we meant in response to EPI. We have now added it in the sentence that now reads "*the best improvement after EPI treatment in total ATP content and cellular proliferation rates of all the cells analyzed.*" (page 27)

24. Too many sentences simply aren't grammatically complete: line 550: Our results in mdx mice show that ATP hydrolysis EPI is sufficient to partially restore muscle force.

The sentence now reads *"Our results in mdx mice show that ATP hydrolysis EPI is sufficient to partially restore muscle force"* (page 28)

Overall there are a lot of awkward language and a lack of clarity especially in the introduction and discussion that tends to undermine the message a little. I suspect this is the result of heavy editing where it is easy to leave legacy text behind. A careful read and careful editing could much improve the paper.

We thank the reviewer for this remark. We have done a careful read and editing now.

Referee #2:

This is a revised version of the same m/s submitted in June, 2022. The authors addressed a large number of concerns identified by 3 reviewers.

Summary - from before. The studies reported in this manuscript detail the effect of epicatechin on the activity of the ATP synthase. Specifically, the authors conclude that epicatechin inhibits ATP hydrolysis, but not ATP synthesis, of the ATP synthase by binding to the same site as the natural inhibitor protein, IF1. Furthermore, the studies suggest that epicatechin can help in the treatment of diseases that affect the electron transport chain and the ATP synthase, including Duchene Muscular dystrophy. In this context, epicatechin, is shown to increase the ATP content in the mitochondria and improve muscle force without an increase in mitochondria content.

Review: Overall, this manuscript has the potential to be an important finding. However, the

number of issues throughout the manuscript places some doubt on the entire findings. I think that the manuscript still needs to be cleaned up as to not drag down the significance of the findings.

The m/s is divided into to 2 sections. The first part tries to identify the mode of action of epicatechin and proposes that it inhibits the mitochondrial ATP synthase in a manner and at the same site, as the natural inhibitor protein. The studies demonstrating this conclusion are not straightforward and not direct. I believe that if this is the hypothesis, that direct studies on the purified F1 ATPase is the best and most direct approach as suggested earlier.

There are some issues that I have with the m/s and the author responses. I'll start with the author responses.

For Ref.1., and concerning Fig. 1A, it is proposed that the decrease in the oxygen concentration it the reason for the changes in the OCR. The arguments start with a number of indisputable facts, but not really related to the issue. The added figure in the response suggests that "excessive mitochondrial activity" which results in a lower oxygen concentration is responsible for the decrease in OCR.

But this cannot be correct. The affinity for oxygen by cytochrome oxidase is very high and the reaction is zero order until the very end - nearly anaerobic conditions. Since mixing by the Seahorse apparently reverses this effect, the mixing must bring some other substrate into less limiting conditions.

The second reason why this is not a correct conclusion is based on their data from the first version of the m/s. In Figure 1A, the OCR increases back to normal with the addition of FCCP. Thus, it is not an issue with the oxygen concentration.

We thank the reviewer for this comment. The reviewer's points are valid and we are in agreement with the reviewer. We would like to outline here, why we agree with the reviewer and yet, the issue the reviewer raises are not affecting the conclusions made in this study, while these points are important issues to address for the field, in general.

A. In Figure 1A &B we develop an approach to measure ATP hydrolysis in coupled mitochondria. Our goal is to reduce respiration to minimum in coupled mitochondria. The reason: high level of respiration produces high levels of CO_2 which may result in noise. Remember that we are trying to measure a phenomenon not measured before, so we try to reduce the risk of an artefact. We start with state 3 respiration in the presence of ADP. As with state 3 respiration, there is a gradual decline in ADP until respiration is limited to the rate of uncoupling. When ADP is depleted, we are at state 4 respiration. Achieving state 4 respiration assures that we are studying coupled mitochondria. None of above are new. These are standard state 3 measurements as they are carried on using the Seahorse instrument. With the injection of ATP, we are measuring ATP hydrolysis. We did not add FCCP at this point because our goal was

to study ATP hydrolysis in coupled mitochondria. At this point our experiment starts with the addition of ATP and the measurement of hydrolysis.

B. To the reviewer point about state 3 respiration: The rate at which OCR is declining on the transition from state 3 respiration to state 4 respiration is dependent on a variety of factors, which the reviewer outlined in the critique. However, for the purpose of our study, our goal was not to study state 3 respiration but to reduce respiration to minimum by reaching state 4, during which respiration is limited to uncoupling, as long as fuel is provided. In the next point we discuss the rate of reduction of state 3 to provide a proper answer to the reviewer on the theoretical aspects of respiration, although the mechanisms leading to reduction in OCR during the transition from state 3 to 4 are not the subject of this study and no conclusions are drawn about state 3 respiration from this study.

C. The reviewer made valid and interesting points about state 3 respiration which we are interested to discuss here. First, given the mixing protocol in the Seahorse instrument, we agree with the reviewer that the most likely explanation for the OCR decline that we have measured is a decrease in [ADP], as expected in state 3 respiration. We also agree with the reviewer point that cytochrome c oxidase activity measured in isolation constitutes a zero-order reaction even at low oxygen concentrations. However, empirical evidence shows that oxygen consumption measured in intact/coupled mitochondria has a higher sensitivity to changes in oxygen tension (Wilson, Rumsey et al., 1988). This explanation on limited oxygen availability and its effects decreasing oxygen consumption in the Seahorse instrument has been expanded recently in this review (Divakaruni & Jastroch, 2022). However, given the mixing protocol in the Seahorse instrument, we agree with the reviewer that the most likely explanation for the decline that we have measured is a decrease in ADP, as expected in state 3 respiration.

D. We believe that the same confusion that we have generated for the reviewer will also be shared by the readers. To address this point, we have added explanation in the results section where we describe the events that occurs during the first phase of this experiment and the purpose of running state 3 respiration.

I also do not like how the issue was solved by simply blocking out the data in the figures. The data is pretty important for the reader - if they are to try to interpret the results.

We apologize for the misunderstanding that our response has generated. This set of data that is blocked is not data that is used for any of the calculations or conclusions of this study. And given that the strongest criticism by all reviewers was that we had included too much data that is not directly relevant, we have put as much effort into removing data that is not being used to draw any conclusions.

We would like to explain why this state 3 respiration data is not being used for the study. To be able to measure ATP hydrolysis, our goal in the first minutes of this experiment is to remove a potential source of noise by bringing coupled mitochondria to state 4 respiration. At state 4 respiration is lower and the possibility for noise produced by CO_2 is reduced. Our way to achieve

that goal is to let mitochondria respire on state 3 respiration until they start depleting ADP. The actual dynamic of state 3 respiration and the values of state 3 respiration were not used to draw any conclusion in this experiment. Of course, if the reviewer suggests that these values should be put back into the manuscript, we will be happy to do so.

For reviewer 3, comment 2, in regard to oligomycin and frozen mitochondria. The authors respond that "no proton potential exists". But in fact, they frequently look at state 3 and 4. They also state that "both in intact mitochondria and previously frozen mitochondria". This is an example of a confusing statement that is frequent in the m/s. As I understand it, the "previously frozen mitochondria" are in fact, intact. This is an issue in many of the studies where I am not exactly sure what is being assayed.

We thank the reviewer for alerting us to this point of potential confusion. We have now gone through every panel and clarified the experimental set up by adding titles in the panel, by adding information in the legends and by keeping to a standardized the nomenclature of the experimental setup names. We have also included a section in the Supplementary information under the title of Expanded Experimental Information that clarifies the use of different kind of samples.

In the results section we now highlight that we are using previously frozen mitochondria. We have to apologize for this common phrase. Since we published the frozen mitochondria respirometry in EMBO in 2020 (Acin-Perez et al., 2020), it has become a most common protocol in the field and we just got used to speaking this way. We thank the reviewer for alerting us to this point.

The term intact mitochondria refer to mitochondria that have been freshly isolated. When we use previously frozen samples, we are assessing maximal respiratory and maximal hydrolytic capacity. In previously frozen samples, we cannot measure state 3 or state 4 as the inner mitochondrial membranes are pierced and permeable to protons. We have now added a detailed explanation in Expanded Experimental Information.

Some of the issue in the text are:

Line 91. "confirmed" is too strong.

We have substituted confirmed for ”*identified*”.

Line 129. "The increase in acidification happened in both in state 3 or state 4 mitochondria, confirming that ATP hydrolysis occurs independently of the presence of ADP."

I still find this confusing since the addition of external ATP might cause all ADP to be depleted in the matrix, so there would not be a state 3 rate at all.

Indeed, the reviewer is correct that when ATP is injected to state 3 mitochondria, they are not in state 3 anymore, they transition to state 4 as ATP synthesis and thus state 3 respiration ends with the depletion of ADP by the injection of ATP. This is our goal in this experiment, to reduce respiration and transition to a situation where ATP hydrolysis can be measured with low noise.

We have corrected this statement, to improve accuracy and clarify this concern on page 8:

When ATP was injected to mitochondria under state 3 or state 4, acidification was increased in both states (Figure 1D and Figure EV1A). The same response of state 3 and state 4 mitochondria to ATP injection strongly supports that acidification as a result of ATP hydrolysis can even be detected in the presence of high ADP/ATP exchange activity, as the latter only can occur in state 3 mitochondria (state 3 mitochondria have an excess of ADP in the matrix prior to ATP injection).
Coupled mitochondria keep a proton motive force that is expected to prevent ATP hydrolysis by CV. However, the recent discovery that microdomains within the mitochondrial inner membrane have reduced proton motive force provided a scenario for hydrolysis to still occur in coupled mitochondria. The assay described here provides a direct measurement of this phenomenon.

Line 140. "ATP hydrolysis in mitochondria was approximately 20% of the maximal hydrolytic capacity measured in uncoupled mitochondria." I guess the point that is "remarkable" is that there was ATP hydrolysis at all. I don't find this so surprising as mitochondria are always a bit leaky. And when you add ATP, this likely gets exchanged for the ADP in the matrix. So, there is no state 3 respiration.

We thank the reviewer for bringing this up. We should have explained why we find this remarkable. Coupled mitochondria keep a proton motive force that is expected to prevent ATP hydrolysis by CV. For that reason, it has always been assumed that so long as mitochondria with intact respiratory function keep a membrane potential that can support a proton motive force, ATP hydrolysis does not take place. And as such, the only scenario during which hydrolysis has been considered is when oligomycin reversal point is achieved, which means oligomycin depolarizes rather than hyperpolarizes. For that reason, we find it remarkable that coupled mitochondria can be found to be engaged in ATP hydrolysis.

In the revised manuscript we now further explain this point and include references explaining the oligomycin reversal point. On page 4 now it can read as:

However, if membrane potential is maintained by reverse action of ATP synthase, oligomycin will induce depolarization, a phenomenon termed oligomycin reversal point (Connolly, Theurey et al., 2018). Oligomycin reversal has been demonstrated in extreme conditions such as hypoxia, complete blockade of respiratory chain activity with antimycin A, and treatment with uncouplers at very high concentrations. However, weather ATP hydrolysis occurs in coupled respiring mitochondria has not been demonstrated

Line 197. Quercetin does not inhibit ETC as such. It should be noted that it does not bind to the IF1 binding site. Neither of the 22 references support the claim.

We agree with the statement from the reviewer. This statement by the reviewer is the reason why we did not pursue testing whether quercetin was a blocker of CV-mediated ATP hydrolysis, despite that *in silico* our screening identified that quercetin could potentially bind to the IF1 site. We agree that the effects described for quercetin modulating respiration could also be a consequence of its antioxidant activity. Thus, to illustrate our agreement with the reviewer and avoid any ambiguity, on page 11, we changed the sentence to:

"*This contrasts with quercetin which has been reported to promote changes in oxidative respiration and redox status*"

Line 241. It would be good to indicate the concentration of mitochondria and IF1. Also, please distinguish between GFP-IF1 and IF1.

When referring to Figure 3, IF1 and GFP-IFI refer to purified recombinant proteins. We have now added a sentence in the methods describing the amount of protein used for this kind of experiments.

Line 258. What is F488?

F488=Fluorescence at 488 nm excitation

Line 259. Competed is a strong statement.

The way we understand competition is not by binding to exact same site. Our data shows that there is competition, although the identity of the binding site at this point is by in silico analysis.

Even if two compounds bind approximately to the same site, the evidence for competition is by the level of binding and not by the site of binding.

We changed it to "*prevented binding to*"

Line 329. Confirm is a strong statement.

We changed it to "*support*"

Line 254. Competes is a strong statement.

We changed it to "*could prevent binding to*"

Line 269. Oligomycin does not inhibit F1 as it is shown to do in this figure.

In Figure EV2H and J, it is shown how oligomycin inhibits F1 in gel ATP hydrolytic activity (left and middle panels) without affecting the assembly of F1 (right panel). We have now fixed the text to refer to only the panels of CV monomer and CV oligomer where we see F1 inhibition by oligomycin. Now, on page 14 it reads as: "*Interestingly, both EPI and oligomycin inhibited ATP hydrolysis, not only in the fully assembled CV and its oligomers, but also in the F_1 subcomplex (Figure EV2H and J and Appendix Figure 4)*"

Line 348. Is the signal linear? It also reports ADP, albeit not as strongly.

The signal is not linear, as in our hands an increase on mtATP was observed after 1h AA treatment, a result submitted in the first version of the manuscript. However, following a reviewer recommendation we decided to remove the data.

The arguments over whether ATP is imported through the reversed ANT is all confusing and adds unnecessary complication to an already complex paper.

Our argument was that the result could be caused by ANT reversal, which we could not further explore.

Line 381. ECAR is never defined and is not actually correct in this use.

ECAR (Extracellular acidification rate) was defined in the abbreviation list as suggested in the first round of revisions as well as when describing the results on page 17.

Line 391. Not statistically significant, P=0.4. In fact, lots of the reported data are not significant.

We believe that the reviewer is referring to Figure 4B in the previous submission, which showed a p=0.4098 for the displacement of IF1 from the assembled CV measured by BNGE in control cells. We tried to be careful to not conclude on results that are not significant, so different experiments were performed to draw conclusions. In this case, we kept the panel of BNGE for all the cells used in Figure EV4 and added to the text in page 15:

BNGE analysis of control cells cultured for 24 hours in the presence or absence of EPI led to a decrease in the levels of ATPIF1 bound to assembled CV in presence of EPI, this was a tendency that did not reach statistical significance (Figure EV4C-D)

We highlighted this non-statistically significant trend because the PLA assay, which is more quantitative than BNGE gels used in Figure 4B, showed a statistically significant and a clear decrease in IF1 binding to CV.

Line 414. This is a rather remarkable conclusion that IF1 inhibits ATP synthesis in vivo.

We are not the first to draw this conclusion. Other studies provided data supporting this conclusion as reviewed here (Garcia-Aguilar & Cuezva, 2018). We know that this is a controversial area and we tried our best to interpret our data in a balanced manner according to the available literature and views.

Line 822. Competitive binding has not been demonstrated

We added two components (EPI and IF1) that shared a binding site in CV, either together or sequentially, and then determined how their binding to CV was affected. We have assessed the competition by *in vitro* binding in isolated mitochondria as well as in intact cells by PLA. The behavior we observed is consistent with competition for binding to CV. However, we acknowledge that we only observed EPI bound in the IF1 binding site using *in silico* analyses. Therefore, there is a possibility that EPI blocks ATP hydrolysis in CV, by binding to other sites in CV, and prevents IF1 binding by conformational changes. Since we don't know whether they are both binding exactly to the same site in CV, we are using the term competitive binding to CV.

Overall, for all of the figure legends, I would recommend a more expansive description of the experiment, the amounts, the method, the sample, ... It is very difficult to decipher how the experiment was done.

We have tried to make the figure legends more descriptive and that is the reason why they are much longer than in the first manuscript submitted. The information describing the methods is included in the method section. The type of samples used as well as the aim of the experiment is explained in the results section when describing the figures.

Overall, this is certainly an important study and may provide a new drug that may modulate the disease phenotype of subset of mitochondrial diseases. However, the studies in the first part do it no service.

We agree with the reviewer that the manuscript is dense and that it provides two major concepts. However, we cannot eliminate the first part, as it is delineating an essential methodology to determine the mechanism of action of this new drug. The current form of our manuscript is providing evidence of the role of ATP hydrolysis and its pharmacological blockage *in silico*, *in vitro* in isolated mitochondria, in human cell culture models of mitochondrial deficiencies to finally an *in vivo* mouse disease model. Therefore, we believe that the present form of the manuscript provides a compelling report with essential data needed to support our main conclusions.

Referee #3:

The authors have improved the description of their findings in the manuscript and significantly increased its clarity. Although the authors have satisfactorily addressed most of my points, I am still not convinced that they can exclude that other proteins than the F1FO ATP synthase affect acidification in an oligomycin-dependent manner. Therefore, using acidification to monitor directionality of the ATP synthase might be an over-simplification. Regardless, I feel that the authors provide overall compelling evidence in support of their central hypothesis that the EPI treatment (and selective inhibition of the ATPase) can have beneficial effects, if the membrane potential can be maintained.

The reviewer's point about the possibility that other ATPases are contributing to acidification rates is valid. While we cannot rule out potential contributions from unknown ATP hydrolases, we believe the following points making such contributions significant is unlikely:

1. Although this is a possibility, other oligomycin-sensitive hydrolyzes have not been identified in the mitochondria
2. CV is the most abundant protein in the mitochondria. If there are unknown Oligomycin-sensitive ATP hydrolases, these will need to be expressed at very high concentration to provide a significant contribution of hydrolysis beyond CV.
3. Previous experiments by the lab of Divakaruni have shown that acidification can be completely eliminated by blocking the ANT, indicating that ATP hydrolysis is the only contributor to acidification (Divakaruni, Andreyev et al., 2018).
4. We have reduced the risk of incorrect interpretation of the data describing EPI effect on ATP hydrolysis by measuring hydrolysis in purified complexes and in native gels where other hydrolases are removed.
5. CV has higher sensitivity to oligomycin with EC50 that is approximately 50 fold lower than oligomycin effect on other ATPases such as Na/K ATPases.

Two minor points: The authors specified now also the EPI concentrations used in the various experiments. I am wondering whether they always used (+) EPI in all experiments or also natural EPI (containing only small amounts of (+) EPI) or even commercially available (-) EPI, for instance in the mouse feeding experiments. The EPI isoform used should be clarified in all experiments.

When studying the binding of the different compound to Complex V, we have used both isomers, (-) and (+) Epicatechin. The latter shows higher affinity biding to Complex V. From there on, we are only using the (+) isomer, with this (+) being referred to as EPI. We have now clarified this aspect in the abbreviation list as well as the text. In the *mdx* study, we are also using the (+) isomer. Previous studies in the literature have been using the (-) isomer.

Finally, the Western blot in Fig. 7 showing OPA1 processing is of low quality and I am wondering how specific bands were quantified. This is however only of minor relevance for the conclusions of the authors and could also be omitted.

Regarding OPA1 quantification in Figure 7, we agree with the referee that the gels do not allow to distinguish the different 5 isoforms of OPA1. However, we can distinguish the OPA1 cleavage form that has much lower molecular weight. This unique OPA1 cleaved form has been reported to be associated with apoptosis (Alaimo, Gorojod et al., 2014, Loucks, Schroeder et al., 2009). We are quantifying its abundance upon the different conditions and treatments.

REFERENCES:

Acin-Perez R, Benador IY, Petcherski A, Veliova M, Benavides GA, Lagarrigue S, Caudal A, Vergnes L, Murphy AN, Karamanlidis G, Tian R, Reue K, Wanagat J, Sacks H, Amati F, Darley-Usmar VM, Liesa M, Divakaruni AS, Stiles L, Shirihai OS (2020) A novel approach to measure mitochondrial respiration in frozen biological samples. EMBO J 39: e104073

Alaimo A, Gorojod RM, Beauquis J, Munoz MJ, Saravia F, Kotler ML (2014) Deregulation of mitochondria-shaping proteins Opa-1 and Drp-1 in manganese-induced apoptosis. PLoS One 9: e91848

Connolly NMC, Theurey P, Adam-Vizi V, Bazan NG, Bernardi P, Bolanos JP, Culmsee C, Dawson VL, Deshmukh M, Duchen MR, Dussmann H, Fiskum G, Galindo MF, Hardingham GE, Hardwick JM, Jekabsons MB, Jonas EA, Jordan J, Lipton SA, Manfredi G et al. (2018) Guidelines on experimental methods to assess mitochondrial dysfunction in cellular models of neurodegenerative diseases. Cell Death Differ 25: 542-572

D'Aurelio M, Vives-Bauza C, Davidson MM, Manfredi G (2010) Mitochondrial DNA background modifies the bioenergetics of NARP/MILS ATP6 mutant cells. Hum Mol Genet 19: 374-86

Divakaruni AS, Andreyev AY, Rogers GW, Murphy AN (2018) In situ measurements of mitochondrial matrix enzyme activities using plasma and mitochondrial membrane permeabilization agents. Anal Biochem 552: 60-65

Divakaruni AS, Jastroch M (2022) A practical guide for the analysis, standardization and interpretation of oxygen consumption measurements. Nat Metab 4: 978-994

Fernandez-Vizarra E, Bugiani M, Goffrini P, Carrara F, Farina L, Procopio E, Donati A, Uziel G, Ferrero I, Zeviani M (2007) Impaired complex III assembly associated with BCS1L gene mutations in isolated mitochondrial encephalopathy. Hum Mol Genet 16: 1241-52

Garcia-Aguilar A, Cuezva JM (2018) A Review of the Inhibition of the Mitochondrial ATP Synthase by IF1 in vivo: Reprogramming Energy Metabolism and Inducing Mitohormesis. Front Physiol 9: 1322

Loucks FA, Schroeder EK, Zommer AE, Hilger S, Kelsey NA, Bouchard RJ, Blackstone C, Brewster JL, Linseman DA (2009) Caspases indirectly regulate cleavage of the mitochondrial fusion GTPase OPA1 in neurons undergoing apoptosis. Brain Res 1250: 63-74

Pastores GM, Santorelli FM, Shanske S, Gelb BD, Fyfe B, Wolfe D, Willner JP (1994) Leigh syndrome and hypertrophic cardiomyopathy in an infant with a mitochondrial DNA point mutation (T8993G). Am J Med Genet 50: 265-71

Wang L, Yuan L, Zeng X, Peng J, Ni Y, Er JC, Xu W, Agrawalla BK, Su D, Kim B, Chang YT (2016) A Multisite-Binding Switchable Fluorescent Probe for Monitoring Mitochondrial ATP Level Fluctuation in Live Cells. Angew Chem Int Ed Engl 55: 1773-6

Wilson DF, Rumsey WL, Green TJ, Vanderkooi JM (1988) The oxygen dependence of mitochondrial oxidative phosphorylation measured by a new optical method for measuring oxygen concentration. J Biol Chem 263: 2712-8

2nd Revision - Editorial Decision 11th Jan 2023

Dear Orian,

The second revision of your manuscript has now been seen by three referees whose comments are enclosed. Please address the remaining questions and comments of referee 2 in the text wherever appropriate, marking your alterations in Track Changes mode. Please also recheck the text for typos as recommended by referee 1.

In addition, I have some minor editorial issues for correction:

Please remove the Highlights section form the manuscript text and include it as a separate file. This will be included on our website (see http://emboj.embopress.org/). Please also send a half-page sized summary figure for the electronic table of contents on the website.

Please complete the Author Checklist, specifically the "Animal observed in or captured from the field" response

EV figure legends from the file "Supplementary Information" should be included in the manuscript file. The legend for Table EV1 should be included in Excel file as a separate tab, and Table EV1 should be renamed to Dataset EV1 with the corresponding callout

The file "Supplementary Information" should be removed

Nomenclature in the Appendix file should be corrected to Appendix Figure S1-S6 in Table of Contents. Please also update figure legends and callouts in the manuscript text as a callout for Appendix Figure S6 is missing.

Best wishes and happy new year,

William

William Teale, PhD
Editor
The EMBO Journal
w.teale@embojournal.org

Use the link below to submit your revision:

https://emboj.msubmit.net/cgi-bin/main.plex

Referee #1:

The paper is greatly improved from the original submission and is now more accessible and the message comes across more clearly. There are a surprising number of linguistic and typographical errors in the edited version which will need to be dealt with in proofs - I'm not able to give the time to identify all of these here. This paper makes an interesting and useful contribution to the literature both as a conceptual development and with the identification of an interesting test compound and one hopes that this will inspire experiments from others to explore these issues.

Referee #2:

This is a second revision of the manuscript. My thoughts are below.

I still do not like the blocked-out data in Fig. 1. It may be helpful to others to understand what is happening.

I don't understand why they just show directly with their purified F1Fo, using in solution assays, that EPI directly inhibits ATPase activity? Why rely on in gel activity?

"First, given the mixing protocol in the Seahorse instrument, we agree with the reviewer that the most likely explanation for the OCR decline that we have measured is a decrease in [ADP], as expected in state 3 respiration."

If ADP is limiting, then ATP will certainly be hydrolyzed as the membrane is always leaky for protons.

Line 88. Oligomycin reversal has been demonstrated in extreme conditions such as hypoxia, complete blockade of respiratory chain activity with antimycin A, and treatment with uncouplers at very high concentrations. However, whether ATP hydrolysis occurs in coupled respiring mitochondria has not been demonstrated.

This needs a reference. Also, I don't think oligomycin will have any effect in the presence of uncoupler - at least not "oligomycin reversal".

Line 146. Surprisingly, addition of ATP resulted in a sharp increase in proton release that was oligomycin sensitive, confirming its origin from ATP hydrolysis and indicating ATP hydrolysis occurs in coupled mitochondria.

Why can't this be confirmed using another method? Also, why is this surprising? Isn't this just a matter of the potential across the membrane vs the ΔG of ATP hydrolysis?

Fig. 2A. I still do not understand why EPI inhibits under these conditions. EPI does not seem to bind tightly as 1μM is needed, yet it stays bound during electrophoresis.

Fig. 3A. Why is there ATPase activity in the presence of Oligomycin?

Fig. 3E. Why is the ATPase activity not effected by IF1 or EPI?

Line 796. Does mitotracker respond to the membrane potential? If so, then it does really measure mito mass unless the potential is the same in all the samples.

Line 826. "measured" is not correct unless an absolute number can be provided. This would require a standard curve for ΔF and ATP concentration. I don't think you can state, for instance, that a 50% decrease in fluorescence corresponds to a 50% decrease in ATP. "Assessed" would be better.

Referee #3:

The authors have satisfactorily responded to my concerns on the revised manuscript.

Referee #2:

This is a second revision of the manuscript. My thoughts are below.

I still do not like the blocked-out data in Fig. 1. It may be helpful to others to understand what is happening.

We thank the referee for the suggestion which we accept. In the revised version we have removed the blocking from that part of the graph. Since the data behind the originally blocked area is not directly relevant to the explanation, and its existence may confuse readers unfamiliar with bioenergetics studies, we have made sure that the relevant part stands out by making the rest of it less opaque.

I don't understand why they just show directly with their purified F1Fo, using in solution assays, that EPI directly inhibits ATPase activity? Why rely on in gel activity?

We thank the reviewer for bringing up this valuable point. We have chosen this approach for a number of reasons and we have now highlighted the advantage can the logic behind he choices in the Results and in the Discussion sections.

A) We are using in gel activity because it allows us to specifically determine that ATP hydrolytic activity we measure is arising from fully assembled complex V. A solution assay would not allow to directly assess whether inhibition occurs in fully assembled complex V.
B) By using this approach, we are eliminating the risk that any of the measured hydrolase activities are elicited by contaminating proteins, other than CV.
C) Technically, the XF technology of Seahorse requires that the signal producing elements be immobilized at the bottom of the plate. Purified F1Fo preparation involve solubilized protein that cannot be pelleted down and “immobilized” in a Seahorse well to allow the measurement of acidification rate as an alternative assay to asses ATP hydrolysis in those samples. We have considered treatments that will immobilize the complexes to the plastic, but we preferred avoiding the risk that such intervention may change the complex function.

"First, given the mixing protocol in the Seahorse instrument, we agree with the reviewer that the most likely explanation for the OCR decline that we have measured is a decrease in [ADP], as expected in state 3 respiration."

If ADP is limiting, then ATP will certainly be hydrolyzed as the membrane is always leaky for protons.

We thank the referee for this important comment, which we address in the Results section and in the Discussion. The question is whether we can rule out the possibility that hydrolysis in this experiment is induced by proton leak. The answer is yes and the specific points are:

Proton leak is not sufficient to induce ATP hydrolysis. It is the proton motive force that has to reverse itself due to depolarization. Depolarization will stimulate respiration. Our results show that the addition of ATP did not result in an increase in respiration. To the contrary, it resulted in further inhibition of respiration.

This is the reason we provide data on OCR, to assure that these mitochondria are indeed coupled. If proton leak was induced, we expected to see them respond by increasing OCR, which was not the case.

We have added a paragraph in Extended Experimental Information, under the section *2c. Studying ATP hydrolysis in intact mitochondria,* under the bullet point D: How we address the contribution of proton leak to hydrolysis?

Line 88. Oligomycin reversal has been demonstrated in extreme conditions such as hypoxia, complete blockade of respiratory chain activity with antimycin A, and treatment with uncouplers at very high concentrations. However, whether ATP hydrolysis occurs in coupled respiring mitochondria has not been demonstrated.

This needs a reference. Also, I don't think oligomycin will have any effect in the presence of uncoupler - at least not "oligomycin reversal".

We thank the reviewer for this valuable comment. We have included references for this point. We also thank the referee for spotting this mistake, as indeed oligomycin reversal requires intact membrane resistance (low conductance) to protons, and as such in the presence of a large proton leak, ATP hydrolysis is not building any proton gradient, and therefore, oligomycin cannot be used to demonstrate oligomycin reversal.
In the revised version we removed that mention of uncouplers in this sentence and added the references. Moreover, we have changed the term of oligomycin reversal for “oligomycin null point”.

Line 146. Surprisingly, addition of ATP resulted in a sharp increase in proton release that was oligomycin sensitive, confirming its origin from ATP hydrolysis and indicating ATP hydrolysis occurs in coupled mitochondria.

Why can't this be confirmed using another method?

We thank the referee for highlighting this point, which we believe is key to the novelty of this study. It is in fact the reason why measurements of ATP hydrolytic activity in intact, coupled mitochondria were never performed, until now. The challenge is to use an approach that is compatible with respiring, living mitochondria.

In the literature, methods that describe the measurements of Complex V activity refer to mostly ATP hydrolysis and they use frozen samples and involve indirect measurements of coupled reactions. These include reactions that link ATP hydrolysis to the production of phosphorus or NADH (Galber et al., 2021, Gomez-Puyou et al., 1983, Pullman et al., 1960, Sumner, 1944). This is the case in the ATP- regenerating system that uses NADH absorbance as a readout, where the ATP added reacts with the supplemented pyruvate kinase, lactate dehydrogenase and phosphoenolpyruvate in the assay buffer (Galber et al., 2021). The approach including the measurement of phosphorus is a colorimetric method that uses the phosphorus generated from ATP hydrolysis in a chemical reaction involving ammonium molybdate, sulfuric acid and ferrous sulphate (Sumner, 1944). A common issue of the above methods is that they use reagents that may interfere or modify respiration and therefore add an undesired risk of artefacts.
These different methodologies have been described and discussed in details in our manuscript dedicated to describing the method, accepted last month in *Life Science Alliance*.

Also, why is this surprising? Isn't this just a matter of the potential across the membrane vs the ΔG of ATP hydrolysis?

We agree with the reviewer. The choice of the word "surprisingly" was not ours. It was a request of a referee in the previous round of revisions. We have now changed surprisingly to remarkably. It is remarkable because in coupled, ATP synthesizing mitochondria, where respiration is controlled by ADP, complex V cannot be working in reverse. Therefore, ATP synthesis cannot occur simultaneously with ATP hydrolysis and this binary switch is controlled by the membrane potential. However, the discovery that an individual mitochondrion can have different membrane potential in different domains, raise the possibility that synthesis and hydrolysis will co-occur. Isn't that remarkable?

Fig. 2A. I still do not understand why EPI inhibits under these conditions. EPI does not seem to bind tightly as 1μM is needed, yet it stays bound during electrophoresis.

We thank the referee for pointing this out. Figure 2A-B shows how EPI inhibits ATP hydrolysis in a dose dependent manner (described in the results section). Inhibition is already significant at a rate of 10nM.

Fig. 3A. Why is there ATPase activity in the presence of Oligomycin?

We thank the referee for bringing this point up. In this manuscript, we present 3 configurations of this experiment, which vary in the step where oligomycin or EPI are added. While in Figure 2A oligomycin is added to intact mitochondria, in Figure 2D and Figure 3A, it is added to mitochondria that were permeabilized by a freeze and thaw cycle. Mitochondria are then run on the gel without any additional oligomycin added and only then hydrolytic activity is measured. We find that oligomycin retains its inhibitory effect through the electrophoresis step better if added to intact mitochondria. This difference may be due to increased binding efficiency in intact mitochondria or due to changes in CV structure that occur when mitochondria are permeabilized. In any case, in each experiment where we analyzed in gel ATP hydrolytic activity we are calculating and presenting only the oligomycin-sensitive portion of the signal.
To further assure we address any artifacts, we confirmed the inhibitory effects of EPI on ATP hydrolysis by CV in 6 different types of experiments, were we used 2 different type of analyses and 3 different experimental configurations.

Fig. 3E. Why is the ATPase activity not effected by IF1 or EPI?

We thank the referee for this comment. ATPase activity is indeed affected by IF1 and EPI. Figure 3G shows the quantification of the in-gel activity where all the active bands (CVd and CVm) are considered for quantification. ATPase activity is calculated as activity per loading. For the dimer, note that in each of the samples containing EPI, IF1 or both, ATP hydrolysis activity of CVd (dimer) is reduced to nearly undetectable level. For the monomer, note that the intensity of the band in CVm is reduced in IF1, EPI and EPI +IF1 samples. For the experimental group "EPI + IF1", note in the Coomassie loading control that there is more sample loaded in the gel (Coomassie loading control, bottom panel in Figure 3E), which, when normalized reduces the calculated hydrolysis rate.

Line 796. Does mitotracker respond to the membrane potential? If so, then it does really measure mito mass unless the potential is the same in all the samples

MitoTracker Deep Red (MTDR) does not respond to differences in membrane potential (Acin-Perez *et al.*, 2020a). It binds irreversibly to mitochondrial proteins, which enables the use of Mitotracker to quantify mitochondrial mass.
To address the comment of the referee, we verified that in all instances, we explicitly indicate when MTDR is used as indicator of mitochondrial mass. We also further clarified our choice of MTDR as a membrane potential independent dye in the methods section.
Although conventional fluorescent stains for mitochondria, such as TMRE and rhodamine 123, are sequestered by functioning mitochondria, and sensitive to loss in membrane potential, the MitoTracker probes are mitochondrion-selective stains that are retained during cell fixation.

Line 826. "measured" is not correct unless an absolute number can be provided. This would require a standard curve for ΔF and ATP concentration. I don't think you can state, for instance,

that a 50% decrease in fluorescence corresponds to a 50% decrease in ATP. "Assessed" would be better.

We thank the reviewer for his/her suggestion. We changed “measured” by “assessed”

REFERENCES

Acin-Perez R, Benador IY, Petcherski A, Veliova M, Benavides GA, Lagarrigue S, Caudal A, Vergnes L, Murphy AN, Karamanlidis G, Tian R, Reue K, Wanagat J, Sacks H, Amati F, Darley-Usmar VM, Liesa M, Divakaruni AS, Stiles L, Shirihai OS (2020) A novel approach to measure mitochondrial respiration in frozen biological samples. EMBO J 39: e104073

Galber, C., G. Minervini, G. Cannino, F. Boldrin, V. Petronilli, S. Tosatto, G. Lippe and V. Giorgio (2021). "The f subunit of human ATP synthase is essential for normal mitochondrial morphology and permeability transition." Cell Rep **35**(6): 109111

Gomez-Puyou, A., G. Ayala, U. Muller and M. Tuena de Gomez-Puyou (1983). "Regulation of the synthesis and hydrolysis of ATP by mitochondrial ATPase. Role of Mg2+." J Biol Chem **258**(22): 13673-13679.

Pullman, M. E., H. S. Penefsky, A. Datta and E. Racker (1960). "Partial resolution of the enzymes catalyzing oxidative phosphorylation. I. Purification and properties of soluble dinitrophenol-stimulated adenosine triphosphatase." J Biol Chem **235**: 3322-3329.

Sumner, J. B. (1944). "A Method for the Colorimetric Determination of Phosphorus." Science **100**(2601): 413-414.

3rd Revision - Editorial Decision **24th Jan 2023**

Dear Orian,

We are almost there. Thank you very much for the detailed responses to reviewer 2; they will provide an important coda to the academic record. Before I can finally click 'accept', could you please

i) send me a two sentence summary for the website (along with a re-sized picture [550x400]), and
ii) add page numbers to the Table of Contents file?

Many thanks and best wishes,

William

William Teale, PhD
Editor
The EMBO Journal
w.teale@embojournal.org

Use the link below to submit your revision:

https://emboj.msubmit.net/cgi-bin/main.plex

All editorial and formatting issues were resolved by the authors.

Dear Orian,

I am pleased to inform you that your manuscript has been accepted for publication in the EMBO Journal.

It was really rewarding to have been involved in this manuscript. Many thanks for the opportunity, and congratulations!

Please note that it is EMBO Journal policy for the transcript of the editorial process (containing referee reports and your response letter) to be published as an online supplement to each paper. If you do NOT want this, you will need to inform the Editorial Office via email immediately. More information is available here:
https://www.embopress.org/page/journal/14602075/authorguide#transparentprocess

Your manuscript will be processed for publication in the journal by EMBO Press. Manuscripts in the PDF and electronic editions of The EMBO Journal will be copy edited, and you will be provided with page proofs prior to publication. Please note that supplementary information is not included in the proofs.

You will be contacted by Wiley Author Services to complete licensing and payment information. The required 'Page Charges Authorization Form' is available here: https://www.embopress.org/pb-assets/embo-site/tej_apc.pdf - please download and complete the form and return to embopressproduction@wiley.com

EMBO Press participates in many Publish and Read agreements that allow authors to publish Open Access with reduced/no publication charges. Check your eligibility: https://authorservices.wiley.com/author-resources/Journal-Authors/open-access/affiliation-policies-payments/index.html

Should you be planning a Press Release on your article, please get in contact with embojournal@wiley.com as early as possible, in order to coordinate publication and release dates.

If you have any questions, please do not hesitate to call or email the Editorial Office. Thank you for your contribution to The EMBO Journal.

Best wishes,

William

William Teale, PhD
Editor
The EMBO Journal
w.teale@embojournal.org

** Click here to be directed to your login page: https://emboj.msubmit.net

EMBO Press Author Checklist

Corresponding Author Name: Orian S Shirihai	
Journal Submitted to: EMBO J	
Manuscript Number: EMBOJ-2022-111699R	

USEFUL LINKS FOR COMPLETING THIS FORM
[The EMBO Journal - Author Guidelines]
[EMBO Reports - Author Guidelines]
[Molecular Systems Biology - Author Guidelines]
[EMBO Molecular Medicine - Author Guidelines]

Reporting Checklist for Life Science Articles (updated January
This checklist is adapted from Materials Design Analysis Reporting (MDAR) Checklist for Authors. MDAR establishes a minimum set of requirements in transparent reporting in the life sciences (see Statement of Task: 10.31222/osf.io/9sm4x). Please follow the journal's guidelines in preparing your
Please note that a copy of this checklist will be published alongside your article.

Abridged guidelines for figures
1. Data
The data shown in figures should satisfy the following conditions:
- the data were obtained and processed according to the field's best practice and are presented to reflect the results of the experiments in an accurate and unbiased manner.
- ideally, figure panels should include only measurements that are directly comparable to each other and obtained with the same assay.
- plots include clearly labeled error bars for independent experiments and sample sizes. Unless justified, error bars should not be shown for technical
- if n<5, the individual data points from each experiment should be plotted. Any statistical test employed should be justified.
- Source Data should be included to report the data underlying figures according to the guidelines set out in the authorship guidelines on Data

2. Captions
Each figure caption should contain the following information, for each panel where they are relevant:
- a specification of the experimental system investigated (eg cell line, species name).
- the assay(s) and method(s) used to carry out the reported observations and measurements.
- an explicit mention of the biological and chemical entity(ies) that are being measured.
- an explicit mention of the biological and chemical entity(ies) that are altered/varied/perturbed in a controlled manner.
- the exact sample size (n) for each experimental group/condition, given as a number, not a range;
- a description of the sample collection allowing the reader to understand whether the samples represent technical or biological replicates (including how many animals, litters, cultures, etc.).
- a statement of how many times the experiment shown was independently replicated in the laboratory.
- definitions of statistical methods and measures:
  - common tests, such as t-test (please specify whether paired vs. unpaired), simple χ2 tests, Wilcoxon and Mann-Whitney tests, can be unambiguously identified by name only, but more complex techniques should be described in the methods section;
  - are tests one-sided or two-sided?
  - are there adjustments for multiple comparisons?
  - exact statistical test results, e.g., P values = x but not P values < x;
  - definition of 'center values' as median or average;
  - definition of error bars as s.d. or s.e.m.

Please complete ALL of the questions below.
Select "Not Applicable" only when the requested information is not relevant for your study.

Materials

Newly Created Materials	Information included in the manuscript?	In which section is the information available? (Reagents and Tools Table, Materials and Methods, Figures, Data Availability Section)
New materials and reagents need to be available; do any restrictions apply?	Not Applicable	

Antibodies	Information included in the manuscript?	In which section is the information available? (Reagents and Tools Table, Materials and Methods, Figures, Data Availability Section)
For **antibodies** provide the following information:<br>- Commercial antibodies: RRID (if possible) or supplier name, catalogue number and or/clone number<br>- Non-commercial: RRID or citation	Yes	Informaytion provided in the Material and Methods section

DNA and RNA sequences	Information included in the manuscript?	In which section is the information available? (Reagents and Tools Table, Materials and Methods, Figures, Data Availability Section)
**Short novel DNA or RNA including primers, probes**: provide the sequences.	Not Applicable	

Cell materials	Information included in the manuscript?	In which section is the information available? (Reagents and Tools Table, Materials and Methods, Figures, Data Availability Section)
**Cell lines:** Provide species information, strain. Provide accession number in repository **OR** supplier name, catalog number, clone number, and/**OR** RRID.	Yes	Cell lines are described in the text as well and the Material and Methods section
**Primary cultures:** Provide species, strain, sex of origin, genetic modification status.	Not Applicable	
Report if the cell lines were recently **authenticated** (e.g., by STR profiling) and tested for mycoplasma contamination.	Yes	Cells were tested for mycoplasma contamination periodically. Only mycoplasma negatove cells were used in this study.

Experimental animals	Information included in the manuscript?	In which section is the information available? (Reagents and Tools Table, Materials and Methods, Figures, Data Availability Section)
**Laboratory animals or Model organisms:** Provide species, strain, sex, age, genetic modification status. Provide accession number in repository **OR** supplier name, catalog number, clone number, **OR** RRID.	Yes	Information available in Material and Methods section
**Animal observed in or captured from the field:** Provide species, sex, and age where possible**.**	Yes	Information available in Material and Methods section
Please detail **housing and husbandry conditions**.	Yes	Information available in Material and Methods section

Plants and microbes	Information included in the manuscript?	In which section is the information available? (Reagents and Tools Table, Materials and Methods, Figures, Data Availability Section)
**Plants:** provide species and strain, ecotype and cultivar where relevant, unique accession number if available, and source (including location for collected wild specimens).	Not Applicable	
**Microbes:** provide species and strain, unique accession number if available, and source.	Not Applicable	

Human research participants	Information included in the manuscript?	In which section is the information available? (Reagents and Tools Table, Materials and Methods, Figures, Data Availability Section)
If collected and within the bounds of privacy constraints report on age, sex and gender or ethnicity for all study participants.	Not Applicable	

Core facilities	Information included in the manuscript?	In which section is the information available? (Reagents and Tools Table, Materials and Methods, Figures, Data Availability Section)
If your work benefited from core facilities, was their service mentioned in the acknowledgments section?	Not Applicable	

Design

Study protocol	Information included in the manuscript?	In which section is the information available? (Reagents and Tools Table, Materials and Methods, Figures, Data Availability Section)
If study protocol has been **pre-registered, provide DOI in the manuscript**. For clinical trials, provide the trial registration number **OR** cite DOI.	Not Applicable	
Report the **clinical trial registration number** (at ClinicalTrials.gov or equivalent), where applicable.	Not Applicable	

Laboratory protocol	Information included in the manuscript?	In which section is the information available? (Reagents and Tools Table, Materials and Methods, Figures, Data Availability Section)
Provide DOI OR other citation details if **external detailed step-by-step protocols** are available.	Not Applicable	

Experimental study design and statistics	Information included in the manuscript?	In which section is the information available? (Reagents and Tools Table, Materials and Methods, Figures, Data Availability Section)
Include a statement about **sample size** estimate even if no statistical methods were used.	Not Applicable	
Were any steps taken to minimize the effects of subjective bias when allocating animals/samples to treatment (e.g. **randomization procedure**)? If yes, have they been described?	Not Applicable	
Include a statement about **blinding** even if no blinding was done.	Yes	Information available in Material and Methods section
Describe **inclusion/exclusion criteria** if samples or animals were excluded from the analysis. Were the criteria pre-established?<br><br>If sample or data points were omitted from analysis, report if this was due to attrition or intentional exclusion and provide justification.	Not Applicable	
For every figure, are **statistical tests** justified as appropriate? Do the data meet the assumptions of the tests (e.g., normal distribution)? Describe any methods used to assess it. Is there an estimate of variation within each group of data? Is the variance similar between the groups that are being statistically compared?	Yes	Information available in Material and Methods section and Figure legends

Sample definition and in-laboratory replication	Information included in the manuscript?	In which section is the information available? (Reagents and Tools Table, Materials and Methods, Figures, Data Availability Section)
In the figure legends: state number of times the experiment was **replicated** in laboratory.	Yes	
In the figure legends: define whether data describe **technical or biological replicates**.	Yes	

Ethics

Ethics	Information included in the manuscript?	In which section is the information available? (Reagents and Tools Table, Materials and Methods, Figures, Data Availability Section)
Studies involving **human participants**: State details of **authority granting ethics approval** (IRB or equivalent committee(s), provide reference number for approval.	Not Applicable	
Studies involving **human participants**: Include a statement confirming that **informed consent** was obtained from all subjects and that the experiments conformed to the principles set out in the WMA Declaration of Helsinki and the Department of Health and Human Services Belmont Report.	Not Applicable	
Studies involving **human participants:** For publication of **patient photos**, include a statement confirming that consent to publish was obtained.	Not Applicable	
Studies involving experimental **animals**: State details of **authority granting ethics approva**l (IRB or equivalent committee(s), provide reference number for approval. Include a statement of compliance with ethical regulations.	Yes	Information available in Material and Methods section
Studies involving **specimen and field samples:** State if relevant **permits** obtained, provide details of authority approving study; if none were required, explain why.	Not Applicable	

Dual Use Research of Concern (DURC)	Information included in the manuscript?	In which section is the information available? (Reagents and Tools Table, Materials and Methods, Figures, Data Availability Section)
Could your study fall under dual use research restrictions? Please check biosecurity documents and list of **select agents and toxins** (CDC): https://www.selectagents.gov/sat/list.htm	Not Applicable	
If you used a select agent, is the security level of the lab appropriate and reported in the manuscript?	Not Applicable	
If a study is subject to dual use research of concern regulations, is the name of the **authority granting approval and reference number** for the regulatory approval provided in the manuscript?	Not Applicable	

Reporting

The MDAR framework recommends adoption of discipline-specific guidelines, established and endorsed through community initiatives. Journals have their own policy about requiring specific guidelines and recommendations to complement MDAR.

Adherence to community standards	Information included in the manuscript?	In which section is the information available? (Reagents and Tools Table, Materials and Methods, Figures, Data Availability Section)
State if relevant guidelines or checklists (e.g., **ICMJE, MIBBI, ARRIVE, PRISMA**) have been followed or provided.	Not Applicable	
For **tumor marker prognostic studies,** we recommend that you follow the **REMARK** reporting guidelines (see link list at top right). See author guidelines, under 'Reporting Guidelines'. Please confirm you have followed these guidelines.	Not Applicable	
For **phase II and III randomized controlled trials**, please refer to the **CONSORT** flow diagram (see link list at top right) and submit the CONSORT checklist (see link list at top right) with your submission. See author guidelines, under 'Reporting Guidelines'. Please confirm you have submitted this list.	Not Applicable	

Data Availability

Data availability	Information included in the manuscript?	In which section is the information available? (Reagents and Tools Table, Materials and Methods, Figures, Data Availability Section)
Have **primary datasets** been deposited according to the journal's guidelines (see 'Data Deposition' section) and the respective accession numbers provided in the Data Availability Section?	Not Applicable	
Were **human clinical and genomic datasets** deposited in a public access-controlled repository in accordance to ethical obligations to the patients and to the applicable consent agreement?	Not Applicable	
Are **computational models** that are central and integral to a study available without restrictions in a machine-readable form? Were the relevant accession numbers or links provided?	Yes	Information available in Material and Methods section
If publicly available data were reused, provide the respective **data citations in the reference list**.	Not Applicable	
